# Supplementary material for: Multi-omics phenotyping of the gut-liver axis reveals metabolic perturbations from a low-dose pesticide mixture in rats
Source: Commun Biol. 2021 Apr 14;4:471. doi: 10.1038/s42003-021-01990-w (PMC8046807; doi:10.1038/s42003-021-01990-w)
Supplement: Supplementary file 1 — Supplementary Information [file 42003_2021_1990_MOESM1_ESM.pdf]

Supplemental Material

Multi-omics phenotyping of the gut-liver axis reveals metabolic perturbations from a low-dose pesticide mixture in rats

Robin Mesnage, et al.,

Table of Contents

Table S1. Bacterial strains used in this study……………………………………………. Page 2

Table S2. Analysis of transcriptome with the drugMatrix toxicogenomics database…Page 3

Figure S1. Effects of on the *in vitro* bacterial growth …………………….…………….. Page 4

Figure S2. Effect of DMSO on bacterial growth *E. coli*…………………………………. Page 5

Figure S3. Correlation between gene expression and methylation changes……..….. Page 6

Figure S4. No oxidative damages to DNA were detected……………………………… Page 7

Table S1. Bacterial strains used in this study.

Collection ID	Other ID	Code	Species	Origin
UCMA n°2933	CIP 103888	LB5	*Lactobacillus rhamnosus*	Human, liver abscess
UCMA n°2934		LB6	*Lactobacillus rhamnosus* GG	Human, faeces
UCMA n°5164		LB7	*Lactobacillus rhamnosus*	Milk
UCMA n°2935	ATCC 7469	LB8	*Lactobacillus rhamnosus*	Human
UCMA n°20972	CIP 104456	LB9	*Lactobacillus rhamnosus*	Human, faeces
UCMA n°20973	CIP 102102	LB10	*Lactobacillus rhamnosus*	Human, faeces
UCMA n°6835	CIP 53.126	EC1	*Escherichia coli*	Human, faeces
UCMA n°7218		EC2	*Escherichia coli* O157:H7	Heifer, faeces
UCMA n°7733	ATCC 10798	EC3	*Escherichia coli* K-12	Human
UCMA n°9748	DSM 6601	EC4	*Escherichia coli* nissle 1917	Human
UCMA n°20974	CIP 105182	EC5	*Escherichia coli* O159:H34	Human, faeces
UCMA n°7088		EC6	*Escherichia coli*	Rotten milk
UCMA n°7105		EC7	*Escherichia coli*	Camembert cheese
UCMA n°7217		EC8	*Escherichia coli*	Milk
UCMA n°10529		EC9	*Escherichia coli*	Raw milk

Table S2. Comparison of our transcriptome findings to a list of gene expression signatures collected from various rat tissues after treatments with various drugs from the drugMatrix toxicogenomics database.

Term	Overlap	P-value	Adjusted P-value	Genes
Niacinamide-750 mg/kg in Water-Rat-Liver-3d-up	22/309	1.96E-11	1.54E-07	SRRM2;GDF15;PLK2;TAT;MIF;OPLAH;CLU;AGT;PNRC1;VTN;PSMC5;CYP2C22;CPT2;PSMC4;BAG3;CBS;HSD17B2;PSMC2;CFL1;PMVK;PGK1;JUNB
Ipriflavone-1500 mg/kg in CMC-Rat-Liver-3d-up	20/318	1.47E-09	5.79E-06	CDKN1A;GDF15;PLK2;TAT;MIF;NR0B2;AGT;PNRC1;HEBP1;PDLIM1;CYP2C22;CPT2;PSMC4;BAG3;HSD17B2;PSMC2;CFL1;PGK1;MLYCD;JUNB
Letrozole-250 mg/kg in Corn Oil-Rat-Liver-5d-up	20/319	1.55E-09	4.08E-06	CDKN1A;TSC22D3;TAT;MIF;CLU;AGT;PNRC1;PDLIM1;RGD1309534;CYP2C22;PSMC4;BAG3;CBS;PSMC2;CFL1;ESD;PGK1;ENPP3;JUNB;UOX
Marimastat-1000 uM in DMSO-Rat-Primary rat hepatocytes-0.67d-up	18/276	5.69E-09	1.12E-05	CDKN1A;SLC20A1;GDF15;PLK2;TAT;MRPS18B;LITAF;ZFP36L2;PNRC1;VEGFA;RBM3;PDLIM1;PRPF39;RABEP1;PSMC4;PSMC2;PGK1;JUNB
Valproic Acid-1500 mg/kg in Water-Rat-Liver-3d-up	18/293	1.44E-08	2.27E-05	SRRM2;ACY1;GDF15;TAT;MRPS18B;MIF;NR0B2;CLU;ZFP36L2;PDLIM1;CYP2C22;CPT2;PSMC4;HSD17B2;PSMC2;PMVK;ESD;MLYCD
Phenylhydrazine-78 mg/kg in Water-Rat-Liver-3d-up	19/335	2.06E-08	2.70E-05	CDKN1A;CEBPD;SLC20A1;GDF15;TAT;MIF;LITAF;AGT;ZFP36L2;PNRC1;VEGFA;PDLIM1;VTN;PSMC4;HSD17B2;PGK1;DNAJB9;JUNB;UOX
Sulfaphenazole-1695 mg/kg in Water-Rat-Liver-1d-up	17/282	4.85E-08	5.46E-05	ACY1;TAT;MIF;NR0B2;CLU;AGT;DPP4;PDLIM1;PSMC5;CYP2C22;CPT2;PSMC4;CBS;HSD17B2;PSMC2;PGK1;ENPP3
Mifepristone-3 mg/kg in Corn Oil-Rat-Liver-3d-dn	18/319	5.31E-08	5.23E-05	SRRM2;PLK2;TAT;MIF;NR0B2;CLU;PNRC1;VEGFA;PDLIM1;VTN;CYP2C22;CPT2;PSMC4;CBS;HSD17B2;PSMC2;ESD;PGK1
Olanzapine-23 mg/kg in CMC-Rat-Liver-3d-up	18/335	1.11E-07	9.71E-05	ACY1;GDF15;PLK2;TAT;MIF;NR0B2;AGT;PNRC1;HEBP1;VTN;RGD1309534;CYP2C22;PSMC4;BAG3;PSMC2;ENPP3;JUNB;UOX
Aminoglutethimide-350 mg/kg in CMC-Rat-Liver-1d-up	17/307	1.65E-07	1.30E-04	ACY1;TAT;MRPS18B;MIF;CLU;PDLIM1;PSMC5;RGD1309534;CYP2C22;CPT2;PSMC4;CBS;HSD17B2;PSMC2;CFL1;ESD;PGK1

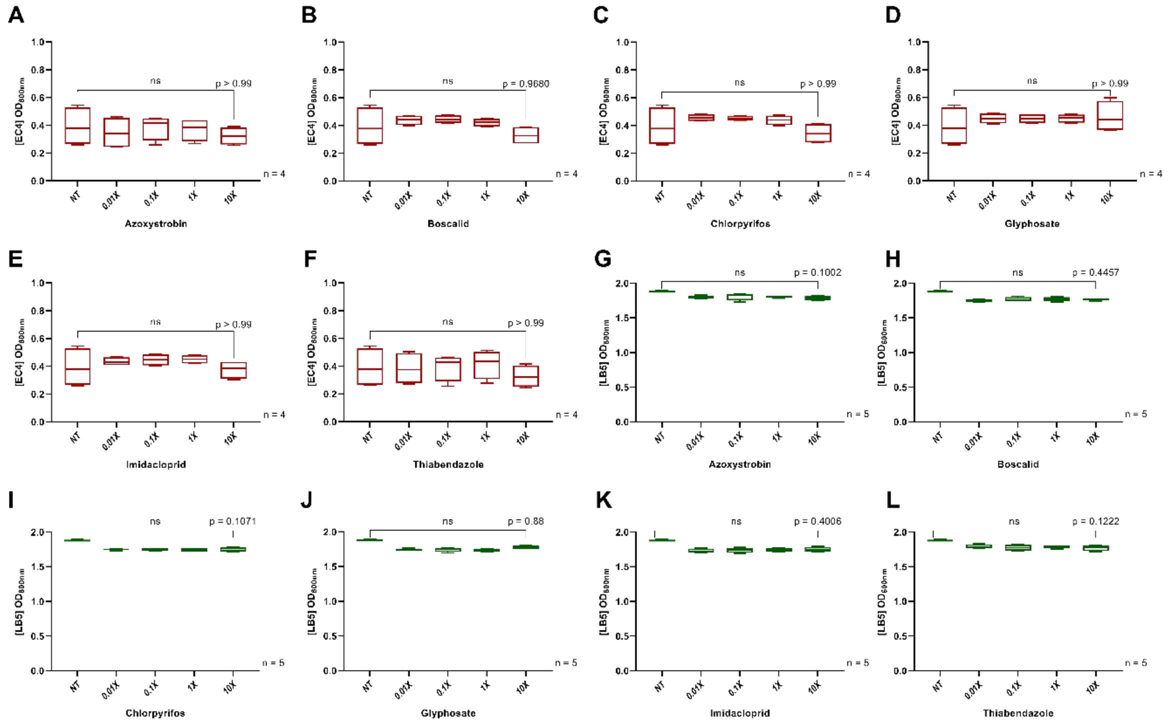

Figure S1. Effects of all the actives principles alone on the *in vitro* bacterial growth of two different species: EC4 and LB5. The bacterial growth of the species *E. coli* (EC4) **(A-F)** is not impacted by any active principles of pesticides tested azoxystrobin **(A)**, boscalid **(B)**, chlorpyrifos **(C)**, glyphosate **(D)**, imidacloprid **(E)** and thiabendazole **(F)**. In the same way, the bacterial growth of the species *L. rhamnosus* (LB5) is not significative impacted by one of the active principles **(G-L)**.

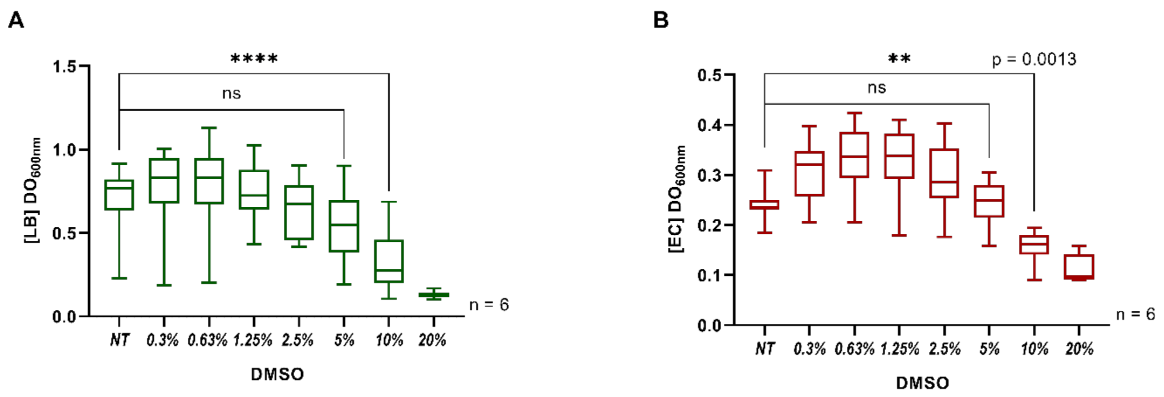

Figure S2. Effect of DMSO on bacterial growth of *L. rhamnosus* and of *E. coli*. The bacterial growth of *L. rhamnosus* **(A)** and *E. coli* **(B)** is not significantly impacted under a concentration of 5% DMSO. Under 1% DMSO, all bacterial strains have growth equivalent to the control (NT).

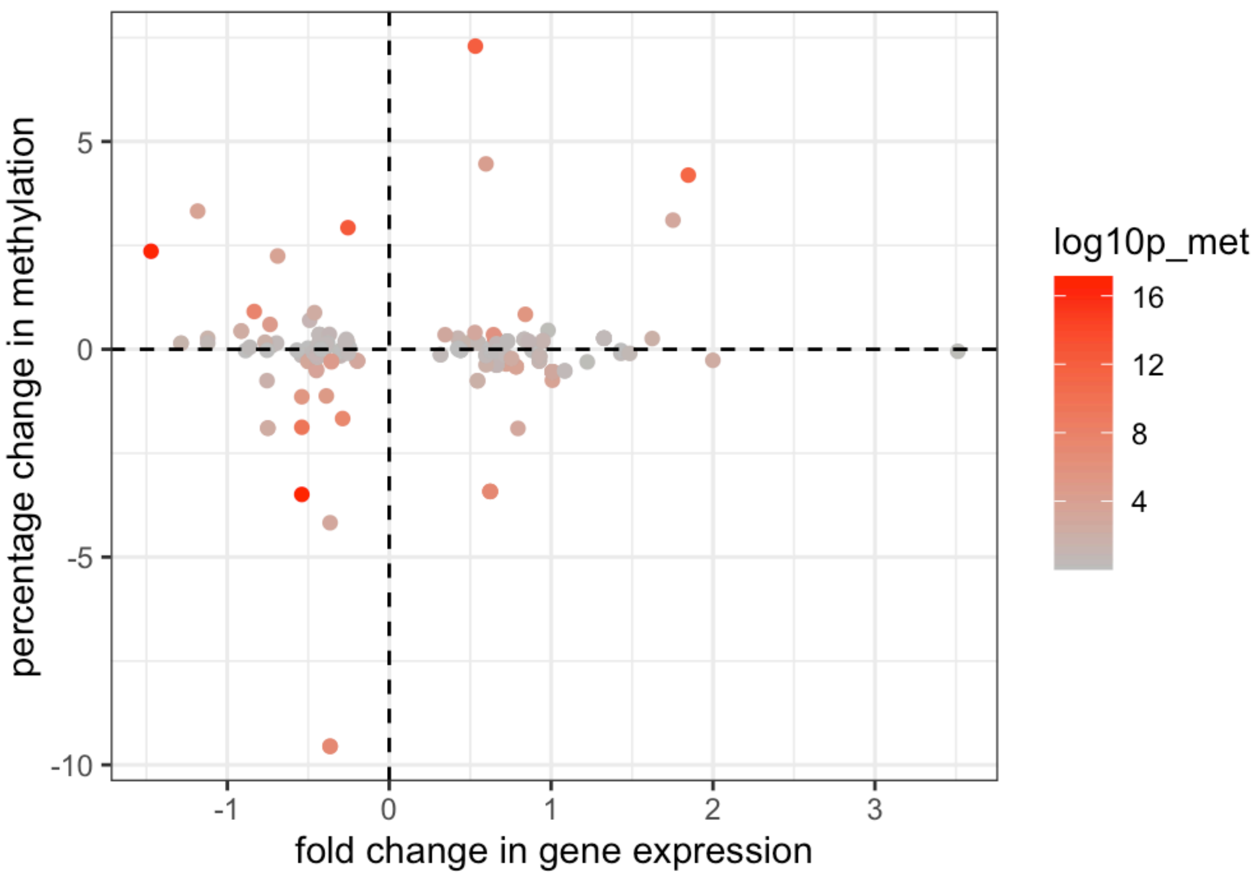

Figure S3. No correlation between the fold changes in gene expression and percentage methylation changes. RRBS of the liver samples was performed to assess if alterations in epigenetic (DNA methylation) status may be responsible at least in part for the treatment-related changes in gene expression patterns. The fold changes in gene expression (RNA-seq) are compared to the percentage of change in methylation (RRBS) for the differentially expressed genes.

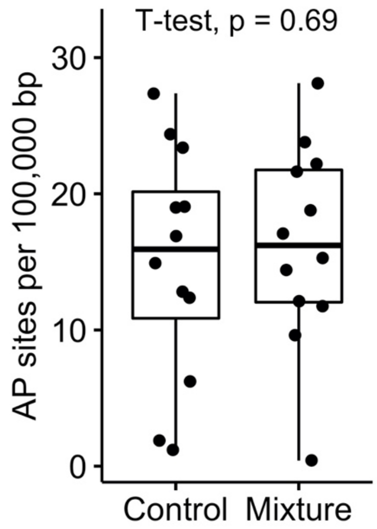

Figure S4. No oxidative damages to DNA were detected. The rate of apurinic/apyrimidinic sites in the liver of rats exposed to the mixture of pesticides was unchanged.
