# Supplementary material for: Multi-omics phenotyping of the gut-liver axis reveals metabolic perturbations from a low-dose pesticide mixture in rats
Source: Commun Biol. 2021 Apr 14;4:471. doi: 10.1038/s42003-021-01990-w (PMC8046807; doi:10.1038/s42003-021-01990-w)
Supplement: Supplementary file 7 — Description of Supplementary Files [file 42003_2021_1990_MOESM7_ESM.pdf]

Description of Additional Supplementary Files

File name: Supplementary Data 1

Description: Tables containing the data used in the statistical analysis.

File name: Supplementary Data 2

Description: Tables containing the results of the transcriptome analysis.

File name: Supplementary Data 3

Description: Table listing differentially methylated CpG.

File name: Supplementary Data 4

Description: Feed analysis to identify possible contaminants.

File name: Supplementary Data 5

Description: R Markdown document containing the code use to perform the analysis, the results of this analysis, as well as the figures as printed by the software.
