# Supplementary material for: Multi-omics phenotyping of the gut-liver axis reveals metabolic perturbations from a low-dose pesticide mixture in rats
Source: Commun Biol. 2021 Apr 14;4:471. doi: 10.1038/s42003-021-01990-w (PMC8046807; doi:10.1038/s42003-021-01990-w)
Supplement: Supplementary file 8 — Reporting Summary [file 42003_2021_1990_MOESM8_ESM.pdf]

nature research

Corresponding author(s): Robin Mesnage and Michael Antoniou

Last updated by author(s): Mar 9, 2021

Reporting Summary

Nature Research wishes to improve the reproducibility of the work that we publish. This form provides structure for consistency and transparency in reporting. For further information on Nature Research policies, see our Editorial Policies and the Editorial Policy Checklist.

Statistics

For all statistical analyses, confirm that the following items are present in the figure legend, table legend, main text, or Methods section.

n/a	Confirmed	
☐	☒	The exact sample size (*n*) for each experimental group/condition, given as a discrete number and unit of measurement
☐	☒	A statement on whether measurements were taken from distinct samples or whether the same sample was measured repeatedly
☐	☒	The statistical test(s) used AND whether they are one- or two-sided<br>*Only common tests should be described solely by name; describe more complex techniques in the Methods section.*
☐	☒	A description of all covariates tested
☐	☒	A description of any assumptions or corrections, such as tests of normality and adjustment for multiple comparisons
☐	☒	A full description of the statistical parameters including central tendency (e.g. means) or other basic estimates (e.g. regression coefficient) AND variation (e.g. standard deviation) or associated estimates of uncertainty (e.g. confidence intervals)
☐	☒	For null hypothesis testing, the test statistic (e.g. *F*, *t*, *r*) with confidence intervals, effect sizes, degrees of freedom and *P* value noted<br>*Give P values as exact values whenever suitable.*
☒	☐	For Bayesian analysis, information on the choice of priors and Markov chain Monte Carlo settings
☐	☒	For hierarchical and complex designs, identification of the appropriate level for tests and full reporting of outcomes
☐	☒	Estimates of effect sizes (e.g. Cohen's *d*, Pearson's *r*), indicating how they were calculated

Our web collection on statistics for biologists contains articles on many of the points above.

Software and code

Policy information about availability of computer code

Data collection

Shotgun metagenomics was performed by GenomeScan (Leiden, The Netherlands). Clustering and DNA sequencing using the NovaSeq6000 (Illumina inc.) was performed according to manufacturer's protocols. A concentration of 1.1 nM of DNA was used. NovaSeq control software NCS v1.6 was used. The shotgun metagenomics data was pre-processed using the pre-processing package v0.2.2 (https://anaconda.org/fasnicar/preprocessing). In brief, this package concatenates reads, to remove Illumina adapters, discard low-quality (quality <20 or >2 Ns) or too short reads (< 75bp), remove phiX and rat genome sequences, and finally sorts and splits the reads into R1, R2, and UN sets of reads. Raw data is available in the SRA with accession number PRJNA609596. Cleaned shotgun metagenomics reads were then processed for taxonomic and pathway profiling. Since there is no gold standard for computational analyses of shotgun metagenomics, we used a combination of approaches. We inferred the taxonomy with the RefSeq database on the metagenomics RAST server, IGGsearch (iggdb_v1.0.0_gut database), MetaPhlAn version 2.9 and Kaiju 1.0.1 .

For the metabolomics data. The MS analysis alternated between MS and data-dependent MSn scans using dynamic exclusion. The scan range varied slightly between methods but covered 70-1000 m/z. Raw data was extracted, peak-identified and QC processed using Metabolon's hardware and software as previously described (DeHavens et al., 2010, J Cheminform 2:9–21.). Serum and caecum metabolites were identified by comparison to libraries of authenticated standards with known retention time/index, mass to charge ratio, chromatographic and MS/MS spectral data. This identification was based on their retention index, the mass match (± 10 ppm), and the (forward or reverse-search) matching between the experimental data and library standards. More than 3300 purified standard compounds were registered into the laboratory information management system. The database server is run with Oracle 10.2.0.1 Enterprise Edition. Raw data is available in Metabolights with accession number MTBLS138.

For RNA-seq transcriptomics, mRNA libraries were prepared using the NEBNext® Poly(A) mRNA Magnetic Isolation Module in combination with the NEBNext® Ultra™ II Directional RNA Library Prep Kit and indexed with NEBNext® Multiplex Oligos for Illumina® (96 Index Primers) (New England Biolabs, Ipswich, Massachusetts, USA). The final library pool was sequenced twice on the NextSeq500 at 1.1pM and 75bp paired-end reads were generated for each library using the Illumina NextSeq®500 v2.5 High-output 150 cycle kit (Illumina Inc., Cambridge, UK). A total of 319,920,579 reads (average of 13,330,024 ± 3,068,802 reads per sample) were generated for the 24 liver samples. The raw

data from the transcriptomics analysis is available at GEO accession number GSE157426.

For DNA methylation analysis, a total of 100ng of total DNA was diluted and processed using the Premium Reduced Representation Bisulfite Sequencing (RRBS) Kit (Diagenode, Denville, NJ, USA) as per the manufacturer's instructions. Pooled libraries were loaded at 1.1M with 20% standard PhiX library (Illumina, CA, USA) and sequenced to 75 base pair single end on a NextSeq 500 (Illumina, CA, USA). Data was aligned to the rat reference genome Rn6 with Bismark. A total of 407,904,185 reads (average of 16,996,008 ± 4,648,420 reads per sample) were generated for the 24 liver samples. The raw data from the RRBS analysis is available at GEO accession number GSE157551.

Data analysis

The metabolome data analysis was performed using R version 3.9. Peak area values were median scaled, log transformed, and any missing values imputed with sample set minimums, both on a per biochemical basis, and separately for each metabolome dataset. Statistical significance was determined using a Welch's two-sample t-test adjusted for multiple comparisons with FDR methods using the R package 'qvalue' version 2.17.0.

For the shotgun metagenomics, a compositional data analysis approach was used since gut metagenomics datasets are typically zero-inflated. We used ALDEx version 2 (ALDEx2) for differential (relative) abundance analysis of proportional data. Statistical analysis for taxa abundance was performed on a dataset corrected for asymmetry (uneven sequencing depths) using the inter-quartile log-ratio method, which identifies features with reproducible variance. Given the relatively small number of samples in this study, we assessed statistical significance using a Wilcoxon test, with p-values adjusted for multiple comparisons with the FDR approach. A multivariate analysis consisting in a non-metric multidimensional scaling (NMDS) plot of Bray-Curtis distances between samples. Statistical significance of the sample clustering was evaluated with a permutational ANOVA (PERMANOVA) analysis on the Bray-Curtis distances with adonis() from vegan v2.4-2.

We also used orthogonal partial least squares discriminant analysis (OPLS-DA) to evaluate the predictive ability of each omics approach. OPLS-DA is an extension of PLS methods, which includes an orthogonal component distinguishing the variability corresponding to the experimental perturbation (here the effects of the pesticide mixture) from the portion of the data that is orthogonal; that is, independent from the experimental perturbation. The R package ropls version 1.20.0 was used. This algorithm uses the nonlinear iterative partial least squares algorithm (NIPALS). Prior to analysis, experimental variables were centred and unit-variance scaled. Since PLS-DA methods are prone to overfitting, we assessed the significance of our classification using permutation tests (permuted 1,000 times).

RNA-seq data was analysed with Salmon. This tool was used to quantify transcript abundance by mapping the reads against a reference transcriptome (Ensembl Release Rattus Norvegicus 6.0 cDNA fasta). Mapping rate was 82.0 ± 4.4% on a rat transcriptome index containing 31,196 targets. The Salmon output was then imported in R version 3.9. using the Bioconductor package tximport. We created a transcript database containing transcript counts, which was used to perform a differential gene expression analysis using DESeq2. We finally used goseq to perform a gene ontology analysis accounting for transcript length biases 60. We also compared our transcriptome findings to a list of gene expression signatures collected from various rat tissues after treatments with various drugs using the drugMatrix toxicogenomics database 61 with EnrichR.

DNA methylation calls from RRBS data were extracted with Bismark. The output from Bismark was then imported in R and analysed with Methylkit. DNA methylation calls were annotated using RefSeq gene predictions for rats (rn6 release) with the package genomation. Other annotations were retrieved using the genome wide annotation for rat tool org.Rn.eg.dbR package version 3.8.2. Statistical analysis was performed with logistic regression models fitted per CpG using Methylkit functions. P-values were adjusted to Q-values using SLIM method 65.

Statistical analyses of in vitro tests on bacterial growth were performed using GraphPad Prism version 8.0.1 (GraphPad Software, Inc, CA, USA). Differences between treatment groups at different concentrations and the negative control were investigated using Kruskal-Wallis one-way ANOVA with Dunn's multiple comparison post-test.

The code used to perform the statistical analysis was compiled an R Markdown document and made available (Supplemental Material).

For manuscripts utilizing custom algorithms or software that are central to the research but not yet described in published literature, software must be made available to editors and reviewers. We strongly encourage code deposition in a community repository (e.g. GitHub). See the Nature Research guidelines for submitting code & software for further information.

Data

Policy information about availability of data

All manuscripts must include a data availability statement. This statement should provide the following information, where applicable:

- Accession codes, unique identifiers, or web links for publicly available datasets
- A list of figures that have associated raw data
- A description of any restrictions on data availability

All data are included in the dataset of the supplemental material, or online at GEO accession number GSE157426 for transcriptomics, GSE157551 for reduced representation bisulfite sequencing, Metabolights accession number MTBLS138 for caecum and serum metabolomics, and at the SRA accession number PRJNA609596 for the shotgun metagenomics. The code used to perform the statistical analysis was compiled an R Markdown document and made available (Supplemental Material).

Field-specific reporting

Please select the one below that is the best fit for your research. If you are not sure, read the appropriate sections before making your selection.

☒ Life sciences ☐ Behavioural & social sciences ☐ Ecological, evolutionary & environmental sciences

For a reference copy of the document with all sections, see nature.com/documents/nr-reporting-summary-flat.pdf

Life sciences study design

All studies must disclose on these points even when the disclosure is negative.

Sample size	Experimental groups consisted of 12 female Sprague-Dawley rats of 8 weeks of age, treated for 90 days, as described in OECD guidelines 408 for testing of the toxicity of chemicals
Data exclusions	No data were excluded
Replication	The repeatability of the findings was assessed by having experimental animals as a biological unit. Given the scale and depth of this animal study, it cannot be conducted multiple times.
Randomization	All the experimental animals were identified by ear punch according to the Jackson Laboratory system. After weaning, and before the start of the experiment, animals were randomised in order to have at most one sister per litter of each group; homogeneous body weight within the different groups was ensured.Cages were identified by a card indicating study protocol code, experimental and pedigree numbers, and dosage group. The cages were periodically rotated on their racks to minimize effects of cage positions on animals.
Blinding	All the high-throughput 'omics' data generation were blinded as only group ID were provided. The animal experiment was not blinded. The data analysis was performed first blinded using unsupervised multivariate data analysis methods and then performed unblinded by using multivariate supervised classification methods

Reporting for specific materials, systems and methods

We require information from authors about some types of materials, experimental systems and methods used in many studies. Here, indicate whether each material, system or method listed is relevant to your study. If you are not sure if a list item applies to your research, read the appropriate section before selecting a response.

Materials & experimental systems

n/a	Involved in the study	
☒	☐	Antibodies
☒	☐	Eukaryotic cell lines
☒	☐	Palaeontology and archaeology
☐	☒	Animals and other organisms
☒	☐	Human research participants
☒	☐	Clinical data
☒	☐	Dual use research of concern

Methods

n/a	Involved in the study	
☒	☐	ChIP-seq
☒	☐	Flow cytometry
☒	☐	MRI-based neuroimaging

Animals and other organisms

Policy information about studies involving animals; ARRIVE guidelines recommended for reporting animal research

Laboratory animals	The experiment was conducted on female young adult Sprague-Dawley rats (8 weeks old at the start of treatment). Female Sprague-Dawley rats from the Cesare Maltoni Cancer Research Center (CMCRC) breeding facility were used. The animals were generated in-house following an outbreed plan and were classified as conventional (minimal disease) status. All the experimental animals were identified by ear punch according to the Jackson Laboratory system. After weaning, and before the start of the experiment, animals were randomised in order to have at most one sister per litter of each group; homogeneous body weight within the different groups was ensured. Animals of 6 weeks of age were acclimatized for two weeks before the start of the experiment.
Wild animals	NA
Field-collected samples	NA
Ethics oversight	The experiment was conducted according to Italian law regulating the use and humane treatment of animals for scientific purposes (Decreto legislativo N. 26, 2014. Attuazione della direttiva n. 2010/63/UE in materia di protezione degli animali utilizzati a fini scientifici. – G.U. Serie Generale, n. 61 del 14 Marzo 2014). Before starting the experiment, the protocol was approved and formally authorized by the ad hoc commission of the Italian Ministry of Health (authorization N. 447/2018-PR)

Note that full information on the approval of the study protocol must also be provided in the manuscript.
