# Supplementary material for: Multi-omics phenotyping of the gut-liver axis reveals metabolic perturbations from a low-dose pesticide mixture in rats
Source: Commun Biol. 2021 Apr 14;4:471. doi: 10.1038/s42003-021-01990-w (PMC8046807; doi:10.1038/s42003-021-01990-w)
Supplement: Supplementary file 9 — Supplementary Data 4 [file 42003_2021_1990_MOESM9_ESM.pdf]

COMPLETE CARE COMPETENCE

Rats, Mice & Hamsters maintenance diet

SAFE A04

www.safe-diets.com

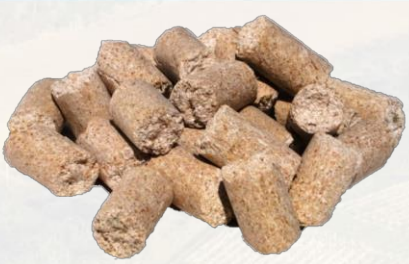

Not contractual picture

Definition

Complete maintenance diet for rats, mice & hamsters.

Product Purpose

Rodent diet for adult and maintenance animals.

To be used within the context of experimental protocols. Does not contain Alfalfa.

Distribution period: from weaning and to adult rodents.

Daily consumption: rats 18 to 25 g, mice 3 to 6 g, hamsters 8 to 12g.

Distribution method: ad libitum or rationed according to experimental protocols.

Product Presentation

15 mm diameter pellet. Can be modified on demand.

Diet	Packaging	Control sheet	Irradiation dose	Animals status	Product code
**SAFE A04**	Paper bag 10 kg	No	None	Conventionnal	U8220G10R
**SAFE A04-10**	Double paper bag 10 kg	No	>10 kiloGrays	Heteroxenic	U8221G10R
**SAFE A04 SP-10**	Paper bag in hermetic plastic pouch 10 kg	No	>10 kiloGrays	Heteroxenic	U8996G10R
**SAFE A04 SP-25**	Paper bag in hermetic plastic Pouch 10 kg	No	>25 kiloGrays	Heteroxenic	U8993G10R
**SAFE R04-10**	Paper bag vacuum packed box of 1x10 kg	No	>10 kiloGrays	Heteroxenic	U8231G10R
**SAFE R04-25**	Paper bag vacuum packed box of 1x10 kg	No	>25 kiloGrays	Heteroxenic	U8232G10R
**SAFE R04-40** 10x1 kg	Double vacuum packed box of 10x1 kg	No	>40 kiloGrays	Axenic and Gnotoxenic	U8233G10R
**SAFE A04C**	Double paper bag 10 kg	Yes	None	Conventionnal	U8224G10R
**SAFE A04C-10**	Double paper bag 10 kg	Yes	>10 kiloGrays	Heteroxenic	U8225G10R

All diets are available with custom packaging, irradiated and with a complete analysis on demand. They are available powdered on demand.

Updated October 05th 2017

SAFE
COMPLETE CARE COMPETENCE
www.safe-diets.com

Rats, Mice & Hamsters maintenance diet

SAFE A04

Nutritional Composition/kg

AMINO ACIDS		TOTAL *
Arginine	mg	9 000
Cystine	mg	2 500
Lysine	mg	7 200
Méthionine	mg	2 800
Tryptophane	mg	1 900
Glycine	mg	8 100

FATTY ACIDS		TOTAL *
Palmitic acid	mg	5 900
Plamitoleic acid	mg	150
Stearic acid	mg	600
Oleic acid	mg	4 800
Linoleic acid	mg	15 000
Linolenic acid	mg	1 200

MINERALS		TOTAL *
P	mg	5 500
Ca	mg	7 300
Na	mg	2 500
K	mg	6 000
Mg	mg	1 600
Mn	mg	70
Fe	mg	270
Cu	mg	16
Zn	mg	55
Cl	mg	4 000

VITAMINS		TOTAL *
Vitamin A	UI	7 500
Vitamin D3	UI	1 000
Vitamin B1	mg	5
Vitamin B2	mg	6,5
Vitamin B5	mg	10
Vitamin B6	mg	3
Vitamin B12	mg	0,01
Vitamin E	UI	30
Vitamin K3	mg	2,5
Niacin	mg	70
Folic Ac.	mg	0,35
Biotin	mg	0,08
Choline	mg	1 600

Pellets Technology

		Mean*
Diameter	mm	16,43
Resistance to crushing	kgf/cm²	22, 7
Resistance to abrasing	%	97,3
Specific mass	g/l	645
Average pellet weight	g	5,319
Average pellet length	mm	22,64

Composition

Barley, wheat, maize, soybean meal, wheat bran, hydrolyzed fish proteins, dicalcium phosphate, pre-mixture of minerals, calcium carbonate, pre-mixture of vitamins.

Centesimal Composition in %

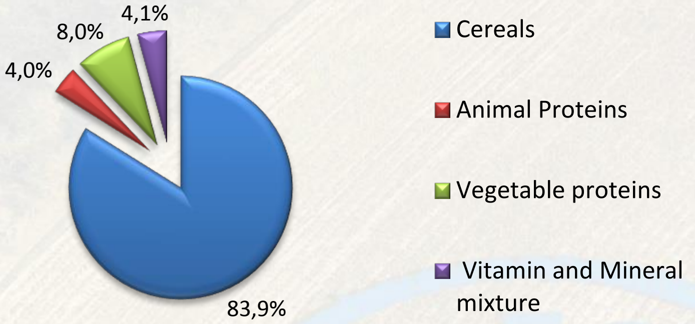

Nutritional Composition in %

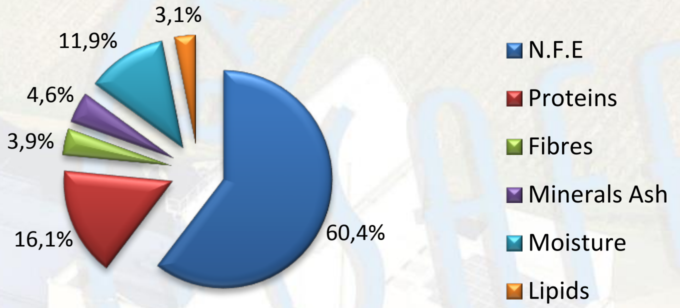

Energy value**	Kcal/kg	Mj/kg	% Proteins	% Lipids	% Carbohydrates
Atwater	3 339	13,97	19,3	8,4	72,4
ME	3 145	13,17	-	-	-

** Energy calcul information:
http://www.safe-diets.com/en/services-r-and-d/diet-energy/

Nitrogen free extract		
- of which starch	(%)	43.5
- of which total sugars	(%)	3.2

* Values are given as an indication only. They are calculated averages of product raw values. They are indicative and have no contractual value. They are subject to variations related to production conditions storage and analytical methods. An analysis is performed on request.

N.F.E.: Nitrogen-free extract, calculated value.

Updated October 05th 2017

T: +33(0)3 86 53 76 90 • Fax: +33(0)3 86 53 35 96 • info@safe-diets.com

eurofins | analytics

Rapport d'analyse

**Echantillon n°**	**370-2017-00194930**	**Date 24/08/2017**	**Page 1/3**
**Rapport d'analyse n°**	**AR-17-AA-186115-01 / 370-2017-00194930**		

SAFE

A l'attention de **Monsieur Renaud BARRAL**
Route de Saint Bris
89290 AUGY
FRANCE
Fax 03 86 53 35 96
Email rbarral@safe-diets.com

Copie à : Monsieur Martel (dmartel@safe-diets.com)

Coordinateur technique de votre dossier : Marion Greloux

**Notre référence :**	370-2017-00194930/ AR-17-AA-186115-01	**Type :**	EX
**Référence client :**	**U8220 V257, CD011344**		
**Description de l'échantillon :**	80% céréales		
**Conditionnement :**	NonCommercial : 672g		
**Votre date de commande :**	10/08/2017	**Votre référence commande :**	10/07/2017 / (EOL) 518-624026
**Date de réception :**	17/08/2017 12:40:00	**Date de mise en analyse :**	17/08/2017
**Prélèvement/Transport :**	dpd		
**Analyses demandées :**	AAU : Formule OC/OP/PYR/naled/temephos/PCB<br>SF00B : Glyphosate, glufosinate, AMPA (alimentation)		
**DLC/DLUO**	/	**N° de lot**	17207
**Marque**	envoi E8797	**Code emballeur**	RB

			Résultats (incertitude)
**SFW9Z**	**SF**	**Analytical run test GC**	
		Analytical run	FAIT

**Pesticides**			Résultats (incertitude)
**SF00B**	**SF**	**Glyphosate, glufosinate, AMPA (alimentation) Méthode : Méthode interne, LC/MS/MS**	
	(a)	Pesticides recherchés	<LOQ
**SFVNS**	**SF**	**PCB avec Screening GC/MS Méthode : § 64 LFGB L 00.00-38 1-4**	
	(a)	PCB 101	<0.005 mg/kg
	(a)	PCB 118	<0.005 mg/kg
	(a)	PCB 138	<0.005 mg/kg
	(a)	PCB 153	<0.005 mg/kg
	(a)	PCB 180	<0.005 mg/kg
	(a)	PCB 28	<0.005 mg/kg
	(a)	PCB 52	<0.005 mg/kg
**SFLA0**	**SF**	**Screening pesticides (GC/MS) Méthode : §64 LFGB L00.00-34, mod.**	
	(a)	Pesticides recherchés	<LOQ
**SFLD0**	**SF**	**Screening pesticides (LC/MS/MS) Méthode : LFGB L 00.00-113**	
	(a)	Butoxyde de Pipéronyle (PBO)	0.034 (± 0.017) mg/kg
	(a)	Autres pesticides recherchés	<LOQ
**SFCEB**	**SF**	**Naled / SF0XA Méthode : Méthode interne, GC/MS**	
	(a)	Naled	<0.05 mg/kg

CONCLUSION (Non couverte par l'accréditation)

Aux limites de quantification des méthodes mises en oeuvre, aucun des paramètres suivants n'a été observé :
somme des 6 PCBs < 0.005 mg/kg
somme de l'Heptachlore et de l'Heptachlorepoxide- cis et -trans < 0.005 mg/kg"

Liste des molécules recherchées et non détectées (* = limite de quantification)

SF00B SF Glyphosate, glufosinate, AMPA (alimentation) (LOQ* mg/kg)

(a) Acide aminométhylphosphonique (AMPA) (.01) | (a) Glufosinate (.01) | (a) Glyphosate (.01)

eurofins | analytics

Rapport d'analyse

Echantillon n° **370-2017-00194930** **Date 24/08/2017**

Rapport d'analyse n° **AR-17-AA-186115-01 / 370-2017-00194930**

SFLA0 SF Screening pesticides (GC/MS) (LOQ* mg/kg)

(a) 2,4,5-T-Méthylester (0.01)	(a) 2,4-D-Méthyl Ester (0.01)	(a) 4,4-Dibromobenzophénone (0.01)	(a) Acetochlor (0.05)	(a) Aclonifen (0.02)	(a) Acrinathrine (0.01)
(a) Alachlore (0.1)	(a) Aldrine (0.005)	(a) Alléthrine (Dépalléthrine) (0.01)	(a) Amidithion (0.01)	(a) Atrazine (0.1)	(a) Azaconazole (0.05)
(a) Azinphos-ethyl (0.01)	(a) Azinphos-methyl (0.01)	(a) Azoxystrobine (0.01)	(a) Benfluraline (0.005)	(a) Benoxacor (0.01)	(a) Benzoylprop-ethyl (0.05)
(a) Béta-endosulfan (0.005)	(a) Bifénox (0.01)	(a) Bifenthrine (0.005)	(a) Binapacryl (0.02)	(a) Bitertanol (0.1)	(a) Boscalide (0.05)
(a) Bromocyclen (0.01)	(a) Bromofenvinphos (0.01)	(a) Bromophos-ethyl (0.005)	(a) Bromophos-méthyl (0.005)	(a) Bromopropylate (0.01)	(a) Buprofezine (0.05)
(a) Butachlore (0.01)	(a) Butamifos (0.005)	(a) Butraline (0.01)	(a) Cadusaphos (0.01)	(a) Captafol (0.01)	(a) Captane (0.01)
(a) Carbophenothion (0.005)	(a) Carbophénothion-méthyl (0.01)	(a) Carfentrazone-ethyl (0.01)	(a) Chinomethionate (0.01)	(a) Chlorbenside (0.005)	(a) Chlordane-cis (0.005)
(a) Chlordane-gamma (=bêta=trans) (0.005)	(a) Chlordécone (0.01)	(a) Chlorethoxyfos (0.005)	(a) Chlorfenapyr (0.005)	(a) Chlorfenprop-méthyl (0.01)	(a) Chlorfenson (0.005)
(a) Chlorfenvinphos (0.01)	(a) Chloridazon (Pyrazon) (0.1)	(a) Chlorméphos (0.005)	(a) Chlorobenzilate (0.005)	(a) Chloroneb (0.1)	(a) Chloropropylate (0.005)
(a) Chlorothalonil (0.005)	(a) Chlorpyrifos (-ethyl) (0.005)	(a) Chlorpyrifos-méthyl (0.005)	(a) Chlorthal diméthyle (0.005)	(a) Chlorthion (0.01)	(a) Chlorthiophos (0.005)
(a) Chlozolinate (0.01)	(a) Cinidon-éthyle (0.025)	(a) Clodinafop-propargyl (0.03)	(a) Coumaphos (0.005)	(a) Crotoxyphos (0.01)	(a) Cyanofenphos (0.01)
(a) Cyanophos (0.01)	(a) Cyfluthrine (0.01)	(a) Cyhalothrine (0.01)	(a) Cyperméthrine (0.005)	(a) Cyphenothrine (0.01)	(a) Cyproconazole (0.05)
(a) DDD, o,p (0.005)	(a) DDE, o,p' (0.005)	(a) DDT (p,p'-DDT+o,p'-DDT+p,p'-DDE+p,p'-TDE) (0.01)	(a) Deltamethrine (0.005)	(a) Dialifos (0.01)	(a) Diallat (0.01)
(a) Diazinon (0.01)	(a) Dicapthon (0.01)	(a) Dichlobénil (0.05)	(a) Dichlofenthion (0.01)	(a) Dichlofluanide (0.005)	(a) Dichloran (0.005)
(a) Dichlorvos (0.025)	(a) Diclofop-méthyl (0.02)	(a) Dicofol (0.005)	(a) Dicofol, o,p- (0.005)	(a) Dicrotophos (0.01)	(a) Dieldrine (0.005)
(a) Dienochlor (0.01)	(a) Difénoconazole (0.02)	(a) Diflufenican (0.01)	(a) Dimefox (0.05)	(a) Dimethachlor (0.01)	(a) Dimethipine (0.01)
(a) Diméthoate (0.02)	(a) Dimethomorphe (0.05)	(a) Diniconazole (0.01)	(a) Dinitramine (0.01)	(a) Dinobuton (0.05)	(a) Disulfoton (0.05)
(a) Disulfoton sulfone (0.02)	(a) Ditalimphos (0.005)	(a) Edifenphos (0.01)	(a) Endosulfan alpha (0.005)	(a) Endosulfan sulfate (0.005)	(a) Endrine (0.005)
(a) EPN (0.01)	(a) Epoxyconazole (0.04)	(a) Etaconazole (0.02)	(a) Ethalfluraline (0.01)	(a) Ethion (0.005)	(a) Ethiprol (0.02)
(a) Ethofumesate (0.2)	(a) Ethoprophos (0.005)	(a) Ethyl parathion (0.005)	(a) Etridiazole (0.005)	(a) Etrimphos (0.005)	(a) Famophos (0.01)
(a) Famoxadone (0.01)	(a) Fenamidone (0.02)	(a) Fenamiphos (0.02)	(a) Fénarimol (0.05)	(a) Fenbuconazole (0.1)	(a) Fenchlorazol (0.01)
(a) Fenchlorphos (0.005)	(a) Fenfluthrine (0.01)	(a) Fenhexamid (0.05)	(a) Fénitrothion (0.005)	(a) Fenoxaprop-éthyle (0.05)	(a) Fenpiclonil (0.05)
(a) Fenpropathrine (0.005)	(a) Fenpropimorphe (0.1)	(a) Fenson (0.01)	(a) Fensulfothion (0.01)	(a) Fenvalerate (RR-/SS-Isomere) (0.005)	(a) Fenvalerate (RS-/SR-Isomere) (0.005)
(a) Fipronil (0.004)	(a) Fipronil desulfinyl (0.004)	(a) Fipronil sulfite (0.005)	(a) Fipronil sulfon (0.005)	(a) Flamprop-isopropyle (0.01)	(a) Flamprop-méthyl (0.01)
(a) Flonicamide (0.05)	(a) Fluazifop-butyl (0.05)	(a) Fluaziname (0.01)	(a) Fluchloraline (0.005)	(a) Flucythrinate (0.01)	(a) Flufenoxuron (0.01)
(a) Fluméthrine (0.01)	(a) Flumetraline (0.005)	(a) Fluopicolid (0.01)	(a) Fluorodifen (0.005)	(a) Fluotrimazole (0.01)	(a) Fluquinconazole (0.01)
(a) Flurenol-Butyl (0.02)	(a) Flurochloridone (0.05)	(a) Flurtamone (0.01)	(a) Flusilazole (0.1)	(a) Folpel (Folpet) (0.01)	(a) Fonofos (0.005)
(a) Formothion (0.01)	(a) Genite (0.01)	(a) Halfenprox (0.01)	(a) Haloxyfop-Ethoxyéthylé (0.01)	(a) Haloxyfop-methyl (0.05)	(a) HCH Alpha (0.005)
(a) HCH Béta (0.005)	(a) HCH Delta (0.005)	(a) HCH, gamma - Lindane (0.005)	(a) HCH-epsilon (0.005)	(a) Heptachlore (0.005)	(a) Heptachlore époxide cis (0.005)
(a) Heptachlore époxide trans (0.005)	(a) Hepténophos (0.01)	(a) Hexachlorobenzène (HCB) (0.005)	(a) Hexaconazole (0.015)	(a) IBP (Iprobenfos) (0.01)	(a) Indanofan (0.02)
(a) Indoxacarbe (0.005)	(a) Iodofenphos (0.005)	(a) Ioxynil-Octanoate (0.01)	(a) Iprodione (0.01)	(a) Isazophos (0.01)	(a) Isobenzane (0.005)
(a) Isocarbofos (0.01)	(a) Isodrine (0.005)	(a) Isofenphos (0.005)	(a) Isofenphos-Méthyl (0.005)	(a) Isomethiozin (0.01)	(a) Isopropalin (0.005)
(a) Isoxadifen-éthyle (0.01)	(a) Kétoendrin-delta (0.005)	(a) Kresoxime-méthyl (0.005)	(a) Lactofen (0.01)	(a) Lambda cyhalothrine (0.01)	(a) Leptophos (0.01)
(a) Lufénuron (0.012)	(a) Malaoxon (degradation Malathion) (0.01)	(a) Malathion (0.005)	(a) Mecarbam (0.01)	(a) Mephosfolan (0.01)	(a) Merphos (0.01)
(a) Métazachlore (0.01)	(a) Méthacrifos (0.05)	(a) Méthidathion (0.012)	(a) Méthoxychlore (0.005)	(a) Métolachlore (0.05)	(a) Metrafenone (0.05)
(a) Métribuzine (0.01)	(a) Mévinphos (0.01)	(a) Mirex (0.005)	(a) Molinate (0.1)	(a) Myclobutanile (0.005)	(a) Nitralin (0.01)
(a) Nitrapyrine (0.02)	(a) Nitrofene (0.01)	(a) Nitrothal-isopropyle (0.01)	(a) Norflurazon (0.05)	(a) Nuarimol (0.01)	(a) Ométhoate (0.1)
(a) Oxadiazon (0.005)	(a) Oxychlordane (0.01)	(a) Oxydéméton methyl (0.02)	(a) Oxyfluorfene (0.01)	(a) Paclobutrazole (0.04)	(a) Paraoxon (0.02)
(a) Paraoxon-méthyl (0.02)	(a) Parathion-méthyl (0.005)	(a) Penconazole (0.02)	(a) Pendiméthaline (0.005)	(a) Pentachloraniline (0.01)	(a) Pentachloroanisole (PCA) (0.005)
(a) Pentachlorobenzène (0.005)	(a) Pentachlorothioanisole (0.01)	(a) Perméthrine (0.02)	(a) Perthane (0.2)	(a) Phenkapton (0.01)	(a) Phénothrine (0.01)
(a) Phenthoate (0.01)	(a) Phosalone (0.01)	(a) Phosfolane (0.01)	(a) Phosmet (0.02)	(a) Picolinafene (0.01)	(a) Picoxystrobin (0.02)
(a) Piperophos (0.01)	(a) Pirimiphos-ethyl (0.01)	(a) Pirimiphos-méthyl (0.01)	(a) Pirimitate (0.05)	(a) Plifenate (0.01)	(a) Pralléthrine (0.01)
(a) Procymidone (0.015)	(a) Profenofos (0.005)	(a) Profluraline (0.005)	(a) Propachlore (0.01)	(a) Propanile (0.01)	(a) Propazine (0.05)
(a) Propétamphos (0.005)	(a) Propiconazole (0.01)	(a) Propyzamide (0.01)	(a) Prothiophos (0.005)	(a) Prothoate (0.01)	(a) Pyraclofos (0.01)
(a) Pyraflufen-éthyl (0.02)	(a) Pyrazophos (0.01)	(a) Pyréthrines (total) (0.02)	(a) Pyridabène (0.005)	(a) Pyridaphenthion (0.01)	(a) Pyrifenox (0.01)
(a) Quinalphos (0.005)	(a) Quinoxyfen (0.015)	(a) Quintozène (0.005)	(a) Quizalofop ethyle (0.02)	(a) Resmethrin (0.1)	(a) S 421 (0.01)
(a) Spiromesifene (0.01)	(a) Sulfotep (0.025)	(a) Sulprofos (0.01)	(a) Swep (0.01)	(a) Tau-fluvalinate (0.005)	(a) Tebupirimfos (0.005)
(a) Tecnazéne (0.005)	(a) Téfluthrine (0.005)	(a) Temephos (0.01)	(a) Terbufos (0.005)	(a) Tetrachlorvinphos (0.005)	(a) Tetraconazole (0.025)
(a) Tétradifon (0.005)	(a) Tétramethrine (0.01)	(a) Tétrasul (0.01)	(a) Tolclofos-méthyl (0.01)	(a) Tolylfluanide (0.01)	(a) Toxaphène Parlar N°26 (0.01)
(a) Toxaphène Parlar N°50 (0.01)	(a) Toxaphène Parlar N°62 (0.01)	(a) Transfluthrin (0.01)	(a) Triadimefone (0.01)	(a) Triadimenole (0.1)	(a) Triallate (0.01)
(a) Triamiphos (0.025)	(a) Triazophos (0.02)	(a) Tribufos (0.01)	(a) Trichloronate (0.005)	(a) Tridiphane (0.05)	(a) Trifloxystrobine (0.01)
(a) Trifluraline (0.005)	(a) Vamidothion (0.05)	(a) Vinclozoline (0.005)			

SFLD0 SF Screening pesticides (LC/MS/MS) (LOQ* mg/kg)

(a) 2,4'-Formoxylidid (métabolite de l'Amitraze) (0.01)	(a) 3-Hydroxycarbofurane (0.005)	(a) 5-Hydroxy-Thiabendazol (0.01)	(a) 6-Chloro-3-phenyl pyridazin-4-ol (0.005)	(a) Abamectine (0.01)	(a) Acéphate (0.005)
(a) Acétamipride (0.005)	(a) Acetochlor (0.02)	(a) Alachlore (0.01)	(a) Aldicarb sulfone (0.01)	(a) Aldicarb sulfoxyde (0.005)	(a) Aldicarbe (0.005)
(a) Ametoctradin (0.01)	(a) Amétryne (0.005)	(a) Amidosulfuron (0.01)	(a) Aminocarbe (0.01)	(a) Amitraze (0.01)	(a) Ancymidol (0.01)
(a) Atrazine (0.005)	(a) Azaconazole (0.01)	(a) Azaméthiphos (0.01)	(a) Aziprotryn (0.01)	(a) Azoxystrobine (0.005)	(a) Bénalaxyl (0.01)
(a) Bendiocarbe (0.005)	(a) Benfuracarbe (0.005)	(a) Benodanil (0.005)	(a) Bénomyl (0.005)	(a) Bensulfuron méthyle (0.005)	(a) Benthiavalicarb-isopropyl (0.005)
(a) Bitertanol (0.01)	(a) Boscalide (0.005)	(a) Bromacile (0.01)	(a) Bupirimate (0.01)	(a) Buprofezine (0.005)	(a) Butachlore (0.02)
(a) Butocarboxim (0.005)	(a) Butocarboxim sulfoxide (0.005)	(a) Butoxycarboxim (0.005)	(a) Buturon (0.005)	(a) Cadusaphos (0.01)	(a) Carbaryl (0.005)
(a) Carbendazime (0.005)	(a) Carbofuran (0.005)	(a) Carbosulfan (0.005)	(a) Carboxine (0.005)	(a) Chlorantraniliprole (0.01)	(a) Chlorbromuron (0.005)
(a) Chlorfluazuron (0.01)	(a) Chloridazon (Pyrazon) (0.005)	(a) Chloroxuron (0.01)	(a) Chlorprophame (0.02)	(a) Chlorsulfuron (0.005)	(a) Chlortoluron (0.005)
(a) Cinidon-éthyle (0.01)	(a) Cinosulfuron (0.005)	(a) Clethodim (0.01)	(a) Clodinafop-propargyl (0.01)	(a) Clofentézine (0.005)	(a) Clomazone (0.005)
(a) Clothianidin (0.01)	(a) Cyanazine (0.01)	(a) Cyazofamide (0.02)	(a) Cymoxanil (0.1)	(a) Cyproconazole (0.005)	(a) Cyprodinile (0.01)
(a) Cyprofuram (0.03)	(a) Cyromazine (0.02)	(a) DEET Diethyltoluamide (0.01)	(a) Demeton (0.01)	(a) Demeton-S-méthyl (0.005)	(a) Demeton-S-methyl-sulfone (0.005)
(a) Desethyl-atrazine (0.005)	(a) Desethyl-Simazine (Désisopropylatrazine) (0.01)	(a) Deséthyl-terbutylazine (0.005)	(a) Desmedipham (0.005)	(a) Desmetryne (0.005)	(a) Diazinon (0.01)
(a) Dichlorvos (0.005)	(a) Diclobutrazole (0.01)	(a) Diethofencarbe (0.005)	(a) Difénoconazole (0.005)	(a) Difénoxuron (0.01)	(a) Diflubenzuron (0.02)
(a) Diflufenican (0.01)	(a) Dimefox (0.05)	(a) Dimefuron (0.01)	(a) Diméthénamide (0.005)	(a) Diméthoate (0.005)	(a) Dimethomorphe (0.01)

Eurofins Analytics France (Nantes)
Rue Pierre Adolphe Bobierre
BP 42301
F-44323 Nantes Cedex 3
FRANCE

Tél. +33 2 51 83 43 40
Fax +33 2 51 83 21 11
ServiceClientEAF@Eurofins.com
www.eurofins.fr

SAS au capital de 3 256 700 €
RCS NANTES 423 190 891
SIRET 423 190 891 00022
APE 743 B

eurofins | analytics

Rapport d'analyse

**Echantillon n°**	**370-2017-00194930**	**Date 24/08/2017**	**Page 3/3**
**Rapport d'analyse n°**	**AR-17-AA-186115-01 / 370-2017-00194930**		

SFLD0 SF Screening pesticides (LC/MS/MS) (LOQ* mg/kg)

(a) Dimetilan (0.005)	(a) Dimoxystrobine (0.005)	(a) Dinotefuran (0.05)	(a) Disulfoton (0.005)	(a) Disulfoton sulfone (0.01)	(a) Disulfoton sulfoxyde (0.01)
(a) Diuron (0.005)	(a) Emamectine (Somme) (0.01)	(a) Epoxyconazole (0.005)	(a) Ethiofencarbe (0.005)	(a) Ethiofencarb-sulfone (0.005)	(a) Ethiofencarb-sulfoxyde (0.005)
(a) Ethiprol (0.01)	(a) Ethofumésat-2-keto (0.05)	(a) Ethofumesate (0.01)	(a) Ethoprophos (0.005)	(a) Etofenprox (0.005)	(a) Etoxazole (0.01)
(a) Famoxadone (0.01)	(a) Fenamidone (0.01)	(a) Fenamiphos (0.01)	(a) Fenamiphos-sulfone (0.01)	(a) Fenamiphos-sulfoxyde (0.01)	(a) Fénarimol (0.005)
(a) Fénazaquine (0.005)	(a) Fenbuconazole (0.005)	(a) Fenhexamid (0.005)	(a) Fenobucarb (0.01)	(a) Fenoxaprop-éthyle (0.01)	(a) Fenoxycarbe (0.005)
(a) Fenpiclonil (0.01)	(a) Fenpropidin (0.01)	(a) Fenpropimorphe (0.005)	(a) Fenpyroximate (0.01)	(a) Fensulfothion (0.01)	(a) Fensulfothione Sulfone (0.01)
(a) Fensulfothion-PO-sulfon (0.01)	(a) Fensulfothion-PO-sulfoxide (0.01)	(a) Fenthion (0.01)	(a) Fenthion-oxone (0.01)	(a) Fenthion-PO-sulfoxid (0.01)	(a) Fenthion-PS-Sulfoxid (0.01)
(a) Fention-PO-sulfon (0.01)	(a) Fention-PS-sulfon (0.01)	(a) Fenuron (0.005)	(a) Flazasulfuron (0.005)	(a) Flonicamide (0.01)	(a) Florasulam (0.005)
(a) Fluazifop-P-butyle (0.005)	(a) Fluazuron (0.02)	(a) Flucycloxuron (0.005)	(a) Fludioxonil (0.01)	(a) Flufenacet (0.01)	(a) Flufenoxuron (0.01)
(a) Fluometuron (0.01)	(a) Fluopicolid (0.01)	(a) Flurochloridone (0.01)	(a) Flurprimidol (0.01)	(a) Flusilazole (0.005)	(a) Flutriafol (0.01)
(a) FM-6-1 (metabolite du Triflumizole) (0.01)	(a) Formetanate (0.01)	(a) Fosthiazate (0.01)	(a) Fuberidazole (0.005)	(a) Furathiocarb (0.005)	(a) Halofenozide (0.005)
(a) Haloxyfop-Ethoxyéthylé (0.005)	(a) Haloxyfop-methyl (0.005)	(a) Hexaconazole (0.01)	(a) Hexaflumuron (0.05)	(a) Hexazinone (0.01)	(a) Hexythiazox (0.005)
(a) Imazalile (0.005)	(a) Imibenconazole (0.01)	(a) Imidaclopride (0.005)	(a) Indoxacarbe (0.005)	(a) Iodosulfuron méthyle (0.005)	(a) Iprovalicarbe (0.005)
(a) Isoprocarb (0.01)	(a) Isoprothiolane (0.01)	(a) Isoproturon (0.005)	(a) Isoxaben (0.01)	(a) Isoxaflutole (0.005)	(a) Isoxathion (0.01)
(a) Lénacile (0.01)	(a) Linuron (0.005)	(a) Lufénuron (0.01)	(a) Malaoxon (degradation Malathion) (0.01)	(a) Malathion (0.01)	(a) Mandipropamide (0.01)
(a) Mepanipyrim (0.005)	(a) Metalaxyl (0.005)	(a) Metamitrone (0.005)	(a) Métazachlore (0.005)	(a) Metconazole (0.01)	(a) Methabenzthiazuron (0.005)
(a) Méthacrifos (0.01)	(a) Methamidophos (0.005)	(a) Méthidathion (0.01)	(a) Methiocarb sulfone (0.01)	(a) Methiocarb Sulfoxyde (0.01)	(a) Méthiocarbe (0.005)
(a) Méthomyl (0.005)	(a) Methoprotryne (0.005)	(a) Methoxyfenozid (0.005)	(a) Metobromuron (0.005)	(a) Métolachlore (0.005)	(a) Metolcarb (0.005)
(a) Métoxuron (0.005)	(a) Metrafenone (0.005)	(a) Métribuzine (0.005)	(a) Metsulfuron méthyle (0.005)	(a) Molinate (0.01)	(a) Monocrotophos (0.005)
(a) Monolinuron (0.005)	(a) Monuron (0.005)	(a) N-2,4-dimethylphenyl-N-methylformamidine (0.05)	(a) Napropamide (0.01)	(a) Néburon (0.005)	(a) Nicosulfuron (0.01)
(a) Novaluron (0.01)	(a) Nuarimol (0.025)	(a) Ofurace (0.005)	(a) Ométhoate (0.01)	(a) Orbencarb (0.005)	(a) Oxadixyl (0.005)
(a) Oxamyl (0.005)	(a) Oxamyl-oxime (0.01)	(a) Oxydéméton methyl (0.005)	(a) Paclobutrazole (0.01)	(a) Paraoxon (0.01)	(a) Paraoxon-méthyl (0.01)
(a) Penconazole (0.005)	(a) Pencycuron (0.01)	(a) Pendiméthaline (0.005)	(a) Pentanochlor (0.01)	(a) Phenmédiphame (0.005)	(a) Phorate (0.01)
(a) Phorate sulfoxyde (0.01)	(a) Phorat-sulfon (0.01)	(a) Phosmet (0.01)	(a) Phosphamidon (0.01)	(a) Phoxime (0.01)	(a) Picoxystrobin (0.005)
(a) Pirimicarb, desmethyl-formamido- (0.005)	(a) Pirimicarbe (0.005)	(a) Pirimicarbe, Desmethyl- (0.005)	(a) Primsulfuron methyl (0.005)	(a) Prochloraz (0.005)	(a) Promecarb (0.005)
(a) Prométone (0.005)	(a) Prométryne (0.005)	(a) Propamocarbe (0.005)	(a) Propargite (0.01)	(a) Propazine (0.005)	(a) Prophame (0.03)
(a) Propiconazole (0.01)	(a) Propoxur (0.005)	(a) Propoxycarbazone (0.01)	(a) Proquinazid (0.005)	(a) Prosulfocarbe (0.01)	(a) Prosulfuron (0.005)
(a) Pymétrozine (0.005)	(a) Pyraclostrobine (0.005)	(a) Pyraflufen-éthyl (0.01)	(a) Pyréthrines (total) (0.5)	(a) Pyridate (0.005)	(a) Pyriméthanil (0.005)
(a) Pyrimidifen (0.01)	(a) Pyriproxyfen (0.005)	(a) Quizalofop ethyle (0.005)	(a) Rabenzazole (0.005)	(a) Rimsulfuron (0.02)	(a) Rotenone (0.01)
(a) Sebuthylazine (0.005)	(a) Sethoxydim (0.01)	(a) Silafluofen (0.05)	(a) Simazine (0.005)	(a) Simeconazole (0.005)	(a) Spinosad (0.005)
(a) Spirodiclofen (0.01)	(a) Spiromesifene (0.01)	(a) Spirotetramate (0.01)	(a) Spiroxamine (0.005)	(a) Tébuconazole (0.005)	(a) Tébufénozide (0.005)
(a) Tébufenpyrad (0.005)	(a) Teflubenzuron (0.05)	(a) TEPP (0.01)	(a) Terbacile (0.01)	(a) Terbufos (0.005)	(a) Terbufos-sulfon (0.005)
(a) Terbufos-sulfoxide (0.005)	(a) Terbuthylazine (0.01)	(a) Terbutryne (0.005)	(a) Tetraconazole (0.01)	(a) Thiabendazole (0.005)	(a) Thiacloprid (0.005)
(a) Thiamethoxam (0.005)	(a) Thiazafluron (0.005)	(a) Thifensulfuron méthyle (0.005)	(a) Thiocarbazil (0.01)	(a) Thiodicarbe (0.005)	(a) Thiofanox (0.01)
(a) Thiofanox-Sulfone (0.005)	(a) Thiofanox-Sulfoxid (0.005)	(a) Thiométon (0.05)	(a) Thionazin (0.01)	(a) Thiophanate-éthyl (0.005)	(a) Thiophanate-méthyl (0.005)
(a) Triadimefone (0.01)	(a) Triadimenole (0.01)	(a) Triamiphos (0.01)	(a) Triasulfuron (0.005)	(a) Triazamate (0.01)	(a) Triazophos (0.005)
(a) Tribenuron methyl (0.01)	(a) Trichlorfon (0.05)	(a) Tricyclazole (0.01)	(a) Tridemorph (0.01)	(a) Trietazine (0.01)	(a) Trifloxystrobine (0.01)
(a) Trifloxysulfuron (0.01)	(a) Triflumizol (0.005)	(a) Triflumuron (0.01)	(a) Triflusulfuron-methyl (0.005)	(a) Triforine (0.01)	(a) Trimethacarb 3.4.5- (0.01)
(a) Triticonazole (0.005)	(a) Uniconazole (0.005)	(a) Vamidothion (0.005)	(a) Vamidothion-sulfone (0.01)	(a) Vamidothion-sulfoxide (0.01)	(a) Zoxamide (0.01)

SIGNATURE

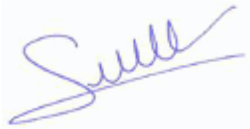

Jérôme Ginet
Business Unit Manager

Rapport validé électroniquement par Jérôme Ginet

NOTE EXPLICATIVE

Ce document ne concerne que l'objet soumis à l'essai ; sa reproduction n'est autorisée que sous sa forme intégrale.
Les essais et rapports sont réalisés conformément à nos conditions générales de vente disponibles sur demande.
Pour déclarer ou non la conformité, l'incertitude associée au résultat a été ajoutée ou retranchée de façon à obtenir sans conteste un résultat opposable aux spécifications ou à la réglementation. Elle n'a pas été prise en compte dans le cadre des référentiels qui intègrent déjà les incertitudes de mesures ou sur demande explicite du client.
Les essais sont identifiés par un code de 5 caractères dont la description précise est disponible sur demande.

Les essais identifiés par le code à 2 lettres SF ont été réalisés par le laboratoire SOFIA (Berlin). Le symbole (a) identifie les prestations couvertes par l'accréditation DIN EN ISO/IEC 17025:2005 DAKKS D-PL-19579-02-00.

Eurofins Analytics France (Nantes)
Rue Pierre Adolphe Bobierre
BP 42301
F-44323 Nantes Cedex 3
FRANCE

Tél. +33 2 51 83 43 40
Fax +33 2 51 83 21 11
ServiceClientEAF@Eurofins.com
www.eurofins.fr

SAS au capital de 3 256 700 €
RCS NANTES 423 190 891
SIRET 423 190 891 00022
APE 743 B

Accréditation(s) N° : 1-2335 N° : 1-2338 Liste des sites accrédités et portée(s) disponible(s) sur www.cofrac.fr

SAINT NOLFF - DEPARTEMENT CHIMIE

CS 40234
56011 VANNES CEDEX
FRANCE

Tél : +33 (0)1 71 25 06 06
Mail : contact@fr.upscience-labs.com

SAFE PF
Route de Saint Bris

89290 AUGY
France

RAPPORT D'ESSAI FINAL
PRODUIT FINI - U8220 V257

Date réception client :	10/08/2017	Demandeur :	M. BARRAL Renaud
Date fabrication :		N° commande :	
N° lot client :	17207	N° client :	E8793
Fournisseur :		N° optim :	
N° lot fournisseur :		N° étude :	
Tonnage :		Réf. commerciale :	
DLUO :		Tiers :	

Date réception labo : 16/08/2017

Masse brute (g): 2003.33

Observations : CD011344

Commentaires :

Le code à 2 lettres indique le site Invivo Labs sur lequel a été réalisée l'analyse : CT = site de Chierry, SN = site de Saint-Nolff.

L'accréditation du Cofrac atteste de la compétence des laboratoires pour les seuls essais couverts par l'accréditation et qui sont identifiés par le fait qu'ils sont soulignés. Les essais soulignés identifiés CT sont couverts par l'accréditation Cofrac n° 1-2338. Les essais soulignés identifiés SN sont couverts par l'accréditation Cofrac n° 1-2335. (portées disponibles sur www.cofrac.fr)

En cas de déclaration de conformité à la spécification, celle-ci ne prend pas en compte l'incertitude associée aux résultats.
Si ce rapport fait mention de résultats de mycotoxines, ils sont corrigés du taux de récupération. Ce rapport d'essai ne concerne que l'échantillon soumis à essai.
Si ce rapport fait mention de résultats de pesticides, ils ne sont pas corrigés du taux de récupération si celui-ci est compris entre 70 et 120 %
« # » : analyse faite plusieurs fois

La reproduction de ce rapport n'est autorisée que sous sa forme intégrale.

Accréditation(s)
N° : 1-2335
N° : 1-2338
Liste
des sites
accrédités
et portée(s)
disponible(s) sur
www.cofrac.fr

ANALYSES CHIMIQUES

Determination	Rés/brut	Rés/sec	Incertitude	Cible	Mini	Maxi	Conforme
CENDRES BRUTES (pour dosage minéraux)<br>Méthode interne CEND-H 13/02 adaptée du Règlement CE 152/2009 du 27-01-2009 - SN	5,2<br>g/100g		0,2<br>g/100g				
FACTEURS ANTITRYPSIQUES<br>AOCS Ba 12-75 - SN	1,0<br>UTI/mg		0,5<br>UTI/mg				
PROTEINES DUMAS (Nx6.25)<br>(#) Méthode interne DUMAS-H 14/02 adaptée de la norme NF EN ISO 16634-1 - décembre 2008 - SN	18,2<br>g/100g		0,5<br>g/100g				
CELLULOSE BRUTE<br>(#) Méthode interne CELL-H 14/01 adaptée du Règlement CE 152/2009 du 27-01-2009 - SN	4,8<br>g/100g		0,8<br>g/100g				
MATIERES GRASSES BRUTES TOTALES<br>Méthode interne - MGRA-H - Procédé B - SN	3,2<br>g/100g		0,5<br>g/100g				
CALCIUM<br>Méthode interne - MINEROL 15 - SN	7713<br>mg/kg		771<br>mg/kg				
PHOSPHORE<br>Méthode interne - MINEROL 15 - SN	6064<br>mg/kg		485<br>mg/kg				
MINERALISATION MICRO ONDES<br>Méthode interne - ELTRACES-H - CT	Réalisée						
ARSENIC<br>Méthode interne - ELTRACES-H - CT	903<br>µg/kg		181<br>µg/kg				
CADMIUM<br>Méthode interne - ELTRACES-H - CT	71<br>µg/kg		14<br>µg/kg				
MERCURE<br>Méthode interne - ELTRACES-H - CT	16<br>µg/kg		6<br>µg/kg				
PLOMB<br>Méthode interne - ELTRACES-H - CT	56<br>µg/kg		11<br>µg/kg				
NITRITES<br>Méthode interne - NIT - SN	<1,0<br>mg/kg exp en NaNO2						
NITRATES<br>Méthode interne - NIT - SN	15<br>mg/kg exp en NaNO3		3<br>mg/kg exp en NaNO3				
AMIDON ENZYMATIQUE<br>(#) NF V18-121 - mars 1997 abrogée - SN	38,8<br>g/100g		1,9<br>g/100g				
SUCRES TOTAUX (exprimés en saccharose)<br>Méthode interne SUCRES-H 14/02 adaptée du Règlement CE 152/2009 du 27-01-2009 - CT	3,7<br>% de saccharose		0,5<br>% de saccharose				
B.H.T. (HPLC)<br>Méthode interne - OXAOAC - SN	<5<br>mg/kg						
B.H.A. (HPLC)<br>Méthode interne - OXAOAC - SN	<5<br>mg/kg						
GALLATE DE PROPYLE<br>Méthode interne - OXAOAC - SN	<5<br>mg/kg						
GALLATE D'OCTYLE<br>Méthode interne - OXAOAC - SN	<5<br>mg/kg						
VITAMINE A<br>Méthode interne VIT_A_E-S - SN	4,0<br>UI/g		1,2<br>UI/g				
VITAMINE E (acétate DL-a-tocophérol)<br>Méthode interne VIT_A_E-S - SN	18,0<br>mg/kg		3,6<br>mg/kg				
POLYPHENOLS TOTAUX (exp. ac.gallique)<br>ISO 14502-1 - mars 2005 - SN	2548<br>mg/kg		255<br>mg/kg				
ACIDE PHYTIQUE<br>Méthode interne - PHYTIC 06/00 - SN	1,0<br>g/100g		0,1<br>g/100g				
PCDD ET PCDF (TEQ-OMS-avec LQ)<br>Analyse sous traitée	0,038<br>ng/kg de m. à 12% d'eau						

Le code à 2 lettres indique le site Invivo Labs sur lequel a été réalisée l'analyse : CT = site de Chierry, SN = site de Saint-Nolff.

L'accréditation du Cofrac atteste de la compétence des laboratoires pour les seuls essais couverts par l'accréditation et qui sont identifiés par le fait qu'ils sont soulignés. Les essais soulignés identifiés CT sont couverts par l'accréditation Cofrac n° 1-2338. Les essais soulignés identifiés SN sont couverts par l'accréditation Cofrac n° 1-2335. (portées disponibles sur www.cofrac.fr)

En cas de déclaration de conformité à la spécification, celle-ci ne prend pas en compte l'incertitude associée aux résultats.
Si ce rapport fait mention de résultats de mycotoxines, ils sont corrigés du taux de récupération. Ce rapport d'essai ne concerne que l'échantillon soumis à essai.
Si ce rapport fait mention de résultats de pesticides, ils ne sont pas corrigés du taux de récupération si celui-ci est compris entre 70 et 120 %
« # » : analyse faite plusieurs fois

La reproduction de ce rapport n'est autorisée que sous sa forme intégrale.

Accréditation(s)
N° : 1-2335
N° : 1-2338
Liste
des sites
accrédités
et portée(s)
disponible(s) sur
www.cofrac.fr

Determination	Rés/brut	Rés/sec	Incertitude	Cible	Mini	Maxi	Conforme
PCB DL (TEQ-OMS-avec LQ)<br>Analyse sous traitée	0,067<br>ng/kg de m. à 12% d'eau						
PCDD/F ET PCB DL (TEQ-OMS-avec LQ)<br>Analyse sous traitée	0,100<br>ng/kg de m. à 12% d'eau						
PCB NDL (6 PCBs hors CB118)<br>Analyse sous traitée	0,270<br>µg/kg de pdt. à 12% d'eau						
<u>EXTRACTION MATIERE GRASSE POUR ACIDES GRAS</u><br>Méthode interne EXTRACPG 99 - SN	FAIT						

cofrac ESSAIS ACCREDITATION
Accréditation(s)
N° : 1-2335
N° : 1-2338
Liste des sites accrédités et portée(s) disponible(s) sur www.cofrac.fr

PROFILS ANALYTIQUES

ACIDES AMINES TOTAUX (sans tyrosine)

Méthode : Règlement CE 152/2009 du 27-01-2009 - SN

Determination	Unité	Résultat	Rés. / sec	Incertitude	TX recouvrement	Cible	Maxi	Conforme
CYSTINE	g/100g	0,29		0,03				
A.ASPARTIQUE	g/100g	1,46		0,12				
PROLINE	g/100g	1,31		0,10				
METHIONINE	g/100g	0,33		0,03				
THREONINE	g/100g	0,65		0,05				
SERINE	g/100g	0,82		0,07				
A.GLUTAMIQUE	g/100g	3,56		0,28				
GLYCINE	g/100g	0,95		0,08				
ALANINE	g/100g	0,86		0,07				
VALINE	g/100g	0,82		0,07				
ISOLEUCINE	g/100g	0,69		0,06				
LEUCINE	g/100g	1,29		0,10				
PHENYLALANINE	g/100g	0,81		0,07				
LYSINE	g/100g	0,89		0,07				
HISTIDINE	g/100g	0,41		0,03				
ARGININE	g/100g	1,04		0,08				
Total des A.aminés quantifiés	g/100g	16,17						

SPECTRE DES SUCRES

Méthode : Méthode interne - SUCRES 14 - CT

Determination	Unité	Résultat	Rés. / sec	Incertitude	TX recouvrement	Cible	Maxi	Conforme
FRUCTOSE	g/100g	<0,2						
GLUCOSE	g/100g	<0,2						
SACCHAROSE	g/100g	1,1		0,2				
LACTOSE	g/100g	<0,4						
MALTOSE	g/100g	<0,4						
SOMME DES SUCRES	g/100g	1,1						

Le code à 2 lettres indique le site Invivo Labs sur lequel a été réalisée l'analyse : CT = site de Chierry, SN = site de Saint-Nolff.

L'accréditation du Cofrac atteste de la compétence des laboratoires pour les seuls essais couverts par l'accréditation et qui sont identifiés par le fait qu'ils sont soulignés.
Les essais soulignés identifiés CT sont couverts par l'accréditation Cofrac n° 1-2338. Les essais soulignés identifiés SN sont couverts par l'accréditation Cofrac n° 1-2335.
(portées disponibles sur www.cofrac.fr)

En cas de déclaration de conformité à la spécification, celle-ci ne prend pas en compte l'incertitude associée aux résultats.
Si ce rapport fait mention de résultats de mycotoxines, ils sont corrigés du taux de récupération. Ce rapport d'essai ne concerne que l'échantillon soumis à essai.
Si ce rapport fait mention de résultats de pesticides, ils ne sont pas corrigés du taux de récupération si celui-ci est compris entre 70 et 120 %
« # » : analyse faite plusieurs fois

La reproduction de ce rapport n'est autorisée que sous sa forme intégrale.

ISOFLAVONES DE SOJA

Méthode : Méthode interne - SOJISOFLAV 06/00 - SN

Determination	Unité	Résultat	Rés. / sec	Incertitude	TX recouvrement	Cible	Maxi	Conforme
DAIDZIN	mg/kg	55,9						
GLYCITIN	mg/kg	<0,5						
GENISTIN	mg/kg	68,1						
DAIDZÉIN	mg/kg	6,0						
GLYCITÉIN	mg/kg	6,5						
GENISTÉIN	mg/kg	4,3						
ISOFLAVONES totaux	mg/kg	140,8						

MYCOTOXINES

Méthode : Méthode interne - MULTIMYC3 Protocole A - CT

Determination	Unité	Résultat	Rés. / sec	Incertitude	TX recouvrement	Cible	Maxi	Conforme
AFLATOXIN B1	μg/kg	<0,5			103			
AFLATOXIN B2	μg/kg	<0,5			91			
AFLATOXINE G1	μg/kg	<0,5			86			
AFLATOXINE G2	μg/kg	<0,5			69			
SOMME DES AFLATOXINES (B1+B2+G1+G2)	μg/kg	<2,0						
OCHRATOXINE A	μg/kg	2,45		1,23	64			
DEOXYNIVALENOL	μg/kg	339		68	85			
SOMME (15A DON & 3A DON)	μg/kg	<50			91			
NIVALENOL	μg/kg	<50			67			
FUSARENONE X	μg/kg	<50			88			
ZEARALENONE	μg/kg	25		6	79			
T2 TOXINE	μg/kg	<50			109			
HT2 TOXINE	μg/kg	<50			89			
SOMME T2 + HT2	μg/kg	<100						
DIACETOXYSCIRPENOL	μg/kg	<50			77			
NEOSOLANIOL	μg/kg	<50			77			
FUMONISINE B1	μg/kg	<10			88			
FUMONISINE B2	μg/kg	<10			66			

Le code à 2 lettres indique le site Invivo Labs sur lequel a été réalisée l'analyse : CT = site de Chierry, SN = site de Saint-Nolff.

L'accréditation du Cofrac atteste de la compétence des laboratoires pour les seuls essais couverts par l'accréditation et qui sont identifiés par le fait qu'ils sont soulignés.
Les essais soulignés identifiés CT sont couverts par l'accréditation Cofrac n° 1-2338. Les essais soulignés identifiés SN sont couverts par l'accréditation Cofrac n° 1-2335.
(portées disponibles sur www.cofrac.fr)

En cas de déclaration de conformité à la spécification, celle-ci ne prend pas en compte l'incertitude associée aux résultats.
Si ce rapport fait mention de résultats de mycotoxines, ils sont corrigés du taux de récupération. Ce rapport d'essai ne concerne que l'échantillon soumis à essai.
Si ce rapport fait mention de résultats de pesticides, ils ne sont pas corrigés du taux de récupération si celui-ci est compris entre 70 et 120 %
« # » : analyse faite plusieurs fois

La reproduction de ce rapport n'est autorisée que sous sa forme intégrale.

MYCOTOXINES

Méthode : Méthode interne - MULTIMYC3 Protocole A - CT

Determination	Unité	Résultat	Rés. / sec	Incertitude	TX recouvrement	Cible	Maxi	Conforme
FUMONISINE B3	µg/kg	<10			75			
SOMME DES FUMONISINES (B1+B2)	µg/kg	<20						

Le code à 2 lettres indique le site Invivo Labs sur lequel a été réalisée l'analyse : CT = site de Chierry, SN = site de Saint-Nolff.

L'accréditation du Cofrac atteste de la compétence des laboratoires pour les seuls essais couverts par l'accréditation et qui sont identifiés par le fait qu'ils sont soulignés.
Les essais soulignés identifiés CT sont couverts par l'accréditation Cofrac n° 1-2338. Les essais soulignés identifiés SN sont couverts par l'accréditation Cofrac n° 1-2335.
(portées disponibles sur www.cofrac.fr)

En cas de déclaration de conformité à la spécification, celle-ci ne prend pas en compte l'incertitude associée aux résultats.
Si ce rapport fait mention de résultats de mycotoxines, ils sont corrigés du taux de récupération. Ce rapport d'essai ne concerne que l'échantillon soumis à essai.
Si ce rapport fait mention de résultats de pesticides, ils ne sont pas corrigés du taux de récupération si celui-ci est compris entre 70 et 120 %
« # » : analyse faite plusieurs fois

La reproduction de ce rapport n'est autorisée que sous sa forme intégrale.

Accréditation(s)
N° : 1-2335
N° : 1-2338
Liste des sites accrédités et portée(s) disponible(s) sur www.cofrac.fr

PROFIL D'ESTERS D'ACIDES GRAS BRUT AVEC TRANS

NF EN ISO 12966-2 - Juin 2011 / NF EN ISO 12966-4 - Août 2015

BILAN

	Composition en	% relatif	mg/100g	val. usuelle	Mini	Maxi
**Acides Gras Saturés**						
Total acides gras saturés	AGS	20,2	511			
Acide palmitique	C16:0	16,5	418			
Acides stéarique	C18:0	2,0	51			
**Acides Gras Insaturés**						
Total acides gras insaturés	AGI	79,8	1967			
Acide oléïque et isomères	C18:1	21,9	544			
Total acides gras mono-insaturés	AGMI	24,9	618			
Total acides gras poly-insaturés	AGPI	54,9	1349			
**Oméga 3**						
Total acides gras omega 3	n-3	6,4	155			
Acide alpha-linolénique (ALA)	ALA	4,2	103			
Acide eicosapentaènoïque (EPA)	EPA	0,6	15			
Acide docosahexaènoïque (DHA)	DHA	1,0	24			
Acide docosapentaènoïque (DPA)	DPA	0,3	<10			
**Oméga 6**						
Total acides gras omega 6	n-6	47,8	1177			
Acide linoléique (LA)	LA	47,5	1170			
**Acides Gras Trans**						
Total acides gras trans (hors CLA)	AGtr tx	1,1	28			
Total 18:1 trans		0,5	12			
**CLA**						
CLA totaux	CLA	nd	nd			

RAPPORTS ET CRITERES SPECIFIQUES

			val. usuelle	Mini	Maxi
LA / ALA	LA / ALA	11,3			
n-6 / n-3	n-6/n-3	7,5			
C18:1 / C16:0	C18:1/C16:0	1,3			
AGS / n-3	AGS/n-3	3,2			
C16:0 / AGS	C16:0/AGS	0,8			
C16:0 / ALA	C16:0/ALA	3,9			
AGPI / ALA	AGPI/ALA	13,1			
C18:1 tr11 / C18:1 tr10	C18:1tr11/C18:1tr10	1,0			
AGS/ALA	AGS/ALA	4,8			

	Composition en	% relatif	mg/100g	val. usuelle	Mini	Maxi
AGMI+C18:0-C16:0		10,4	251			
Acides Gras sur produit sec	(g d'AG/kg de MS)					
ALA sur produit sec	(g d'ALA/kg de MS)					

Le code à 2 lettres indique le site Invivo Labs sur lequel a été réalisée l'analyse : CT = site de Chierry, SN = site de Saint-Nolff.

L'accréditation du Cofrac atteste de la compétence des laboratoires pour les seuls essais couverts par l'accréditation et qui sont identifiés par le fait qu'ils sont soulignés. Les essais soulignés identifiés CT sont couverts par l'accréditation Cofrac n° 1-2338. Les essais soulignés identifiés SN sont couverts par l'accréditation Cofrac n° 1-2335. (portées disponibles sur www.cofrac.fr)

En cas de déclaration de conformité à la spécification, celle-ci ne prend pas en compte l'incertitude associée aux résultats.
Si ce rapport fait mention de résultats de mycotoxines, ils sont corrigés du taux de récupération. Ce rapport d'essai ne concerne que l'échantillon soumis à essai.
Si ce rapport fait mention de résultats de pesticides, ils ne sont pas corrigés du taux de récupération si celui-ci est compris entre 70 et 120 %
« # » : analyse faite plusieurs fois

La reproduction de ce rapport n'est autorisée que sous sa forme intégrale.

cofrac ESSAIS ACCREDITATION
Accréditation(s) N° : 1-2335 N° : 1-2338 Liste des sites accrédités et portée(s) disponible(s) sur www.cofrac.fr

Composition en		%relatif	mg/100g	val. usuelle	Mini	Maxi
Acide butyrique	C 4:0	nd	nd			
Acide valérique	C 5:0	nd	nd			
Acide caproïque	C 6:0	nd	nd			
Acide heptanoïque	C 7:0	nd	nd			
Acide caprylique	C 8:0	nd	nd			
Acide nonaïque	C 9:0	nd	nd			
Acide caprique	C10:0	nd	nd			
Acide caproléique	C10:1	nd	nd			
Acide undécanoïque	C11:0	nd	nd			
Acide undécénoïque	C11:1	nd	nd			
Acide laurique	C12:0	0,1	<10			
Acide lauroléique	C12:1	nd	nd			
Acide 11-méthyl dodécanoïque	C13:0 iso	nd	nd			
Acide 10-méthyl dodécanoïque	C13:0 anteiso	nd	nd			
	total_C13:0	nd	nd			
Acide isomyristique	C14:0 iso	nd	nd			
Acide myristique	C14:0	0,6	15			
	total_C14:0	0,6	15			
Acide myristoléique	C14:1	nd	nd			
Acide 13-méthyl tétradécanoïque	C15:0 iso	nd	nd			
Acide 12-méthyl tétradécanoïque	C15:0 anteiso	nd	nd			
Acide pentadécanoïque	C15:0	0,1	<10			
	total_C15:0	0,1	<10			
Acide cis-10-pentadécènoïque	C15:1 n-5	nd	nd			
Acide pentadécénoïque	C15:1	nd	nd			
	total_C15:1	nd	nd			
Acide isopalmitique	C16:0 iso	nd	nd			
Acide palmitique	C16:0	16,5	418			
	total_C16:0	16,5	418			
Acide hypogéïque	C16:1 n-9	0,1	<10			
Acide palmitoléïque	C16:1 n-7	0,6	16			
Acide hexadécènoïque (autres isomères)	C16:1	<0,05	<10			
	total_C16:1	0,7	19			
Acide hexadécadiènoïque	C16:2	<0,05	<10			
Acide hexadécatriènoïque	C16:3	nd	nd			
Acide hexadécatétraènoïque	C16:4	nd	nd			

Accréditation(s)
N° : 1-2335
N° : 1-2338
Liste des sites accrédités et portée(s) disponible(s) sur www.cofrac.fr

Composition en		% relatif	mg/100g	val. usuelle	Mini	Maxi
Acide isomargarique	C17:0 iso	nd	nd			
Acide 14-méthyl hexadécanoïque	C17:0 anteiso	nd	nd			
Acide margarique	C17:0	0,1	<10			
	total_C17:0	0,1	<10			
Acide 14-méthyl 8-hexadécènoïque	C17:1 anteiso	<0,05	<10			
Acide heptadécènoïque	C17:1	0,1	<10			
	total_C17:1	0,1	<10			
Acide isostéarique	C18:0 iso	nd	nd			
Acide stéarique	C18:0	2,0	51			
	total_C18:0	2,0	51			
Acide trans-4-octadécènoïque	C18:1 tr4	nd	nd			
Acide trans-5-octadécènoïque	C18:1 tr5	nd	nd			
Acide trans-(6-8)-octadécènoïque	C18:1 tr6-8	nd	nd			
Acide élaidique	C18:1 tr9	0,3	<10			
Acide trans-10-octadécènoïque	C18:1 tr10	0,1	<10			
Acide trans-vaccénique	C18:1 tr11	0,1	<10			
Acide trans-12-octadécènoïque	C18:1 tr12	nd	nd			
	total_C18:1tr	0,5	12			
Acide oléique *	C18:1 c9	20,0	496			
Acide cis-10-octadécènoïque *	C18:1 c10	nd	nd			
Acide cis-vaccénique	C18:1 c11	1,3	33			
Acide cis-12-octadécènoïque	C18:1 c12	0,1	<10			
Acide cis-13-octadécènoïque	C18:1 c13	<0,05	<10			
Acide cis-14-octadécènoïque	C18:1 c14	nd	nd			
Acide cis-15-octadécènoïque	C18:1 c15	nd	nd			
Acide cis-16-octadécènoïque	C18:1 c16	nd	nd			
	total_C18:1cis	21,4	532			
	total_C18:1	21,9	544			
Acide linolélaidique	C18:2 n-6 tr	0,1	<10			
Acide octadécadiènoïque (isomère cis-trans)	C18:2 ct	0,3	<10			
Acide octadécadiènoïque (isomère trans-cis)	C18:2 tc	0,2	<10			
	total_C18:2tr	0,6	15			
Acide linoléique (LA)	C18:2 n-6	47,5	1170			
Acide octadécadiènoïque (autres isomères cis)	C18:2 cis	nd	nd			
	total_C18:2cis	47,5	1170			

La reproduction de ce rapport n'est autorisée que sous sa forme intégrale.

Composition en		% relatif	mg/ 100g	val. usuelle	Mini	Maxi
Acide ruménique (CLA)	CLA c9tr11	nd	nd			
Acide linoléique conjugué (CLA)	CLA tr10c12	nd	nd			
Acide linoléique conjugué (CLA, isomères)	CLA	nd	nd			
	total_CLA	nd	nd			
	total_C18:2	48,1	1185			
Acide octadécatriènoïque (isomères trans)	C18:3 tr	0,1	<10			
Acide octadécatriènoïque (isomères cis)	C18:3 cis	nd	nd			
Acide gamma-linolénique (GLA)	C18:3 n-6	nd	nd			
Acide alpha-linolénique (ALA)	C18:3 n-3	4,2	103			
	total_C18:3	4,3	105			
Acide stéaridonique	C18:4 n-3	0,1	<10			
Acide octadécatétraènoïque (autres isomères)	C18:4	nd	nd			
	total_C18:4	0,1	<10			
Acide nonadécanoïque	C19:0	nd	nd			
Acide nonadécènoïque	C19:1	nd	nd			
Acide arachidique	C20:0	0,3	<10			
Acide cis-5-eicosènoïque	C20:1 n-15	nd	nd			
Acide cis-8-eicosènoïque	C20:1 n-12	nd	nd			
Acide gadoleïque	C20:1 n-9	1,3	31			
Acides gadoleïque et isomères	C20:1	nd	nd			
	total_C20:1	1,3	31			
Acide eicosadiènoïque	C20:2 n-6	0,2	<10			
Acide eicosadiènoïque (autres isomères)	C20:2	nd	nd			
	total_C20:2	0,2	<10			
Acide de Mead	C20:3 n-9	nd	nd			
Acide eicosatriènoïque (DGLA)	C20:3 n-6	nd	nd			
Acide eicosatriènoïque (DALA)	C20:3 n-3	0,1	<10			
	total_C20:3	0,1	<10			
Acide arachidonique (AA)	C20:4 n-6	0,1	<10			
Acide eicosatétraènoïque	C20:4 n-3	0,1	<10			
	total_C20:4	0,2	<10			
Acide eicosapentaènoïque (EPA)	C20:5 n-3	0,6	15			
Acide hénéiconanoïque	C21:0	nd	nd			
Acide béhénique	C22:0	0,2	<10			

Le code à 2 lettres indique le site Invivo Labs sur lequel a été réalisée l'analyse : CT = site de Chierry, SN = site de Saint-Nolff.

L'accréditation du Cofrac atteste de la compétence des laboratoires pour les seuls essais couverts par l'accréditation et qui sont identifiés par le fait qu'ils sont soulignés. Les essais soulignés identifiés CT sont couverts par l'accréditation Cofrac n° 1-2338. Les essais soulignés identifiés SN sont couverts par l'accréditation Cofrac n° 1-2335. (portées disponibles sur www.cofrac.fr)

En cas de déclaration de conformité à la spécification, celle-ci ne prend pas en compte l'incertitude associée aux résultats.
Si ce rapport fait mention de résultats de mycotoxines, ils sont corrigés du taux de récupération. Ce rapport d'essai ne concerne que l'échantillon soumis à essai.
Si ce rapport fait mention de résultats de pesticides, ils ne sont pas corrigés du taux de récupération si celui-ci est compris entre 70 et 120 %
« # » : analyse faite plusieurs fois

La reproduction de ce rapport n'est autorisée que sous sa forme intégrale.

cofrac ESSAIS ACCREDITATION
Accréditation(s) N° : 1-2335 N° : 1-2338 Liste des sites accrédités et portée(s) disponible(s) sur www.cofrac.fr

Composition en		% relatif	mg/100g	val. usuelle	Mini	Maxi
Acide cétoléique	C22:1 n-11	0,6	15			
Acide érucique	C22:1 n-9	0,1	<10			
Acide docosènoïque	C22:1 n-7	nd	nd			
	total_C22:1	0,7	18			
Acide docosadiènoïque (n-6)	C22:2 n-6	nd	nd			
Acide docosadiènoïque	C22:2	nd	nd			
	total_C22:2	nd	nd			
Acide docosatriènoïque (n-6)	C22:3 n-6	nd	nd			
Acide docosatriènoïque (n-3)	C22:3 n-3	nd	nd			
	total_C22:3	nd	nd			
Acide docosatétraènoïque (n-6)	C22:4 n-6	nd	nd			
Acide docosatétraènoïque (n-3)	C22:4 n-3	nd	nd			
	total_C22:4	nd	nd			
Acide docosapentaènoïque (n-6)	C22:5 n-6	nd	nd			
Acide docosapentaènoïque (DPA)	C22:5 n-3	0,3	<10			
	total_C22:5	0,3	<10			
Acide docosahexaènoïque (DHA)	C22:6 n-3	1,0	24			
Acide tricosanoïque	C23:0	0,1	<10			
Acide lignocérique	C24:0	0,2	<10			
Acide nervonique	C24:1 n-9	0,2	<10			

nd : pic non présent sur le chromatogramme
NR : non recherché par cette méthode
* : Coélution des isomères
La somme des esters méthyliques d'acides gras correspond à la somme des esters méthyliques d'acides gras identifiés.
La quantification des esters méthyliques d'acides gras est déterminée par étalonnage interne.
Un facteur de correction est utilisé pour le calcul des esters méthyliques d'acides gras de C4 à C10.
Les esters méthyliques d'acides gras de C4 à C7 ne sont pas dans le domaine d'application de la norme. Ils sont toutefois inclus dans la somme des Acides Gras Saturés (AGS).

Incertitude en relatif : 8 % de la teneur relative avec une valeur mini de 0,5 et maxi de 3,5.
Incertitude en absolu : 10 mg/100g pour les teneurs < 50mg/100g, pour les teneurs >= 50mg/100g : 12 % de la valeur.

Conclusion :

Validé le : 04-10-17

M. KERRAND Jérémie

Superviseur

RAPPORT D'ESSAI	ANALYSE DES PCDD ET PCDF, DES PCB "type dioxine" ET DES PCB indicateurs

Accréditation N°1-1531 PORTEE DISPONIBLE SUR WWW.COFRAC.FR

L'essai LSE17-122614-1 a été réalisé à la demande de Date : 07/09/2017

INVIVO LABS
Contact
TALHOUET ST NOLFF
CS 40234
VANNES 56011

Code essai CARSO-LSEH : LSE17-122614-1
Référence client dossier : Réf envoi 170817-027

OBJET DE L'ESSAI

L'objet de ce rapport d'essai référencé sous le code d'essai LSE17-122614 est l'analyse des PCDD et PCDF, et des PCB "type dioxine" et indicateurs.

INFORMATIONS SPECIFIQUES A L'ESSAI

Description	Information
Date de réception des échantillons	LSE1708-48971 18/08/2017
Méthode(s) d'analyse - PCDD/F	LSE1708-48971 MET009
Méthode(s) d'analyse - PCB	MET038
Instrument de mesure HRGC/HRMS<br>Volume injecté en micro-litres	Autospec ULTIMA (Waters)<br>1 à 3 microlitres
Volume final	25-50 microlitres
Observations spécifiques à l'essai :	LSE1708-48971 Rien à signaler

Les méthodes internes sont la traduction technique des référentiels suivants : normes EPA 1613, EPA 1668 et EN 16215 (échantillons agro-alimentaires).
Dans le cas des échantillons agro-alimentaires, les méthodes d'analyse sont conformes aux critères énoncés dans le règlement (UE) n° 771/2017 de la commission du 3 mai 2017 (alimentation animale) et dans le règlement (UE) n° 644/2017 de la commission du 5 avril 2017 (alimentation humaine).

Les prélèvements ont été réalisés par le client.

RESULTATS

Les résultats résumés dans les tableaux ci-dessous sont obtenus en considérant les valeurs des différents congénères au-dessous de la limite de quantification comme étant égales à la limite de quantification (résultat upperbound).

Les résultats complets sont rapportés dans la deuxième partie du rapport.

Résumé des résultats en PCDD/F-TEQ

Référence client échantillon	Référence CARSO-LSEH	PCDD/F-TEQ	Unité		Incertitude élargie (k=2) +/-15%
17SA052492 - PRODUIT FINI - U8220 V257	**LSE1708-48971**	0.038	ng/kg	de matière à 12% eau (TEF OMS 2005)	0.006

Résumé des résultats en PCB-TEQ (PCB "Dioxin-like")

Référence client échantillon	Référence CARSO-LSEH	PCB-TEQ	Unité		Incertitude élargie (k=2) +/-15%
17SA052492 - PRODUIT FINI - U8220 V257	**LSE1708-48971**	0.067	ng/kg	de matière à 12% eau (TEF OMS 2005)	0.010

Société par action simplifiée au capital de 2 283 622,30 € - RCS Lyon B 410 545 313 - SIRET 410 545 313 00042 - APE 7120B — N° TVA: FR 82 410 545 313
Siège social et laboratoire : 4, avenue Jean Moulin — CS 30228 - 69633 VENISSIEUX CEDEX - Tél : (33) 04 72 76 16 16 - Fax : (33) 04 78 72 35 03
Site web : www.groupecarso.com - e-mail : carso@groupecarso.com

Résumé des résultats en PCDD/F-PCB-TEQ (PCDD/F + PCB "Dioxin-like")

Référence client échantillon	Référence CARSO-LSEH	PCDD/F-PCB-TEQ	Unité		Incertitude élargie (k=2) +/-15%
17SA052492 - PRODUIT FINI - U8220 V257	**LSE1708-48971**	0.10	ng/kg	de matière à 12% eau (TEF OMS 2005)	0.02

Résumé des résultats en PCB (6 PCBs hors PCB118)

Référence client échantillon	Référence CARSO-LSEH	PCB NDL (6 PCBs hors PCB118)	Unité		Incertitude élargie (k=2) +/-15%
17SA052492 - PRODUIT FINI - U8220 V257	**LSE1708-48971**	0.27	µg/kg	de matière à 12% eau	0.04

Conformités aux réglementations

Référence client échantillon	Référence CARSO-LSEH	Conformité
17SA052492 - PRODUIT FINI - U8220 V257	**LSE1708-48971**	Conforme au règlement (UE) N° 277/2012 du 28 Mars 2012.

Pour déclarer ou non la conformité à la spécification, l'incertitude associée au résultat a été prise en compte.

La déclaration de conformité concerne uniquement les composés demandés par le client pour lesquels les réglementations en vigueur s'appliquent.

Dans le cas d'échantillons contenant de la matière grasse, le pourcentage est déterminé par pesée.

Dans le cas d'échantillons dont la teneur en eau est communiquée, cette dernière est déterminée par dessication puis pesée de la perte de poids de l'échantillon.

La reproduction de ce document n'est autorisée que sous la forme de fac-similé photographique intégral.
Il comporte 4 pages.

Le rapport établi ne concerne que les échantillons soumis à l'essai.
L'accréditation du COFRAC atteste de la compétence des laboratoires pour les seuls essais couverts par l'accréditation, identifiés par le symbole #.

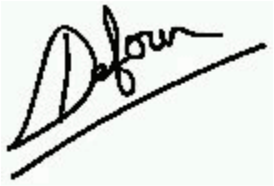

Stéphanie DEFOUR

Responsable de Laboratoire

Société par action simplifiée au capital de 2 283 622,30 € - RCS Lyon B 410 545 313 - SIRET 410 545 313 00042 - APE 7120B — N° TVA: FR 82 410 545 313
Siège social et laboratoire : 4, avenue Jean Moulin — CS 30228 - 69633 VENISSIEUX CEDEX - Tél : (33) 04 72 76 16 16 - Fax : (33) 04 78 72 35 03
Site web : www.groupecarso.com - e-mail : carso@groupecarso.com

Essai LSE17-122614 : Echantillon LSE1708-48971

Client INVIVO LABS

Date : 07/09/2017

Référence client échantillon 17SA052492 - PRODUIT FINI - U8220 V257

Teneur en eau (%) : 12.71
Teneur en matière grasse (%) : 3.05
Matière à 12% eau (g) : 19.79

Date de début d'analyse : 18/08/2017
Fichiers HRGC/HRMS-PCDD/F : 05SEPV38 - PCB: 05SEPU36 05SEPU36 05SEPV38

	ng/kg de matière à 12% eau	Taux de récupération %	Cofrac
2,3,7,8-TeCDD	<0.0051	74	#
1,2,3,7,8-PeCDD	<0.0126	86	#
1,2,3,4,7,8-HxCDD	<0.0126	71	#
1,2,3,6,7,8-HxCDD	<0.0126	71	#
1,2,3,7,8,9-HxCDD	<0.0126		#
1,2,3,4,6,7,8-HpCDD	<0.1010	81	#
OcCDD	0.2184	73	#
2,3,7,8-TeCDF	0.0487	60	#
1,2,3,7,8-PeCDF	<0.0126	70	#
2,3,4,7,8-PeCDF	0.0153	75	#
1,2,3,4,7,8-HxCDF	<0.0126	63	#
1,2,3,6,7,8-HxCDF	<0.0126	66	#
2,3,4,6,7,8-HxCDF	<0.0126	70	#
1,2,3,7,8,9-HxCDF	<0.0126	70	#
1,2,3,4,6,7,8-HpCDF	<0.0253	72	#
1,2,3,4,7,8,9-HpCDF	<0.0253	74	#
OcCDF	<0.0505	73	#
PCDD/F-TEQ lower bound (TEF OMS 2005)	0.0095		#
PCDD/F-TEQ medium bound (TEF OMS 2005)	0.024		#
PCDD/F-TEQ upper bound (TEF OMS 2005)	0.038		#
PCB 77	2.8970	49	#
PCB 81	<0.1010	46	#
PCB 105	11.6385	102	#
PCB 114	<2.0202	102	#
PCB 118	42.1377	95	#
PCB 123	<2.0202	96	#
PCB 126	0.6144	68	#
PCB 156	4.6307	93	#
PCB 157	<2.0202	93	#
PCB 167	3.4084	84	#
PCB 169	<0.1010	91	#
PCB 189	<2.0202	44	#
PCB-TEQ lower bound (TEF OMS 2005)	0.064		#
PCB-TEQ medium bound (TEF OMS 2005)	0.065		#
PCB-TEQ upper bound (TEF OMS 2005)	0.067		#
PCDD/F-PCB-TEQ lower bound (TEF OMS 2005)	0.073		#
PCDD/F-PCB-TEQ medium bound (TEF OMS 2005)	0.089		#
PCDD/F-PCB-TEQ upper bound (TEF OMS 2005)	0.10		#
PCB 28	<10.10	69	#
PCB 52	14.77	66	#
PCB 101	29.98	67	#
PCB 138	67.62	94	#
PCB 153	117.71	74	#
PCB 180	31.67	78	#
	µg/kg de matière à 12% eau		
PCB NDL (6 PCBs hors PCB118) lower bound	0.26		#
PCB NDL (6 PCBs hors PCB118) medium bound	0.27		#
PCB NDL (6 PCBs hors PCB118) upper bound	0.27		#

Lorsque la concentration en analyte est précédée de « < », le résultat communiqué correspond à la limite de quantification (LOQ).

Légende : LOQ = Limite de quantification
Lower bound : La valeur 0 est affectée aux congénères <LOQ
Medium bound : La valeur ½ LOQ est affectée aux congénères <LOQ
Upper bound : La valeur de leur LOQ est affectée aux congénères <LOQ

Dans le cas des échantillons agro-alimentaires, la limite de quantification est telle que définie dans l'annexe I du règlement (UE) n° 644/2017. Il s'agit de la concentration de l'analyte dans l'extrait qui produit une réponse instrumentale aux deux ions suivis avec un rapport S/B (signal sur bruit) de 3:1 pour le signal le moins intense et remplit les critères d'identification tels que définis dans la méthode EPA 1613, Révision B.

Société par action simplifiée au capital de 2 283 622,30 € - RCS Lyon B 410 545 313 - SIRET 410 545 313 00042 - APE 7120B — N° TVA: FR 82 410 545 313
Siège social et laboratoire : 4, avenue Jean Moulin — CS 30228 - 69633 VENISSIEUX CEDEX - Tél : (33) 04 72 76 16 16 - Fax : (33) 04 78 72 35 03
Site web : www.groupecarso.com - e-mail : carso@groupecarso.com

Concentration des 17 congénères toxiques

Référence CARSO-LSEHL : LSE1708-48971

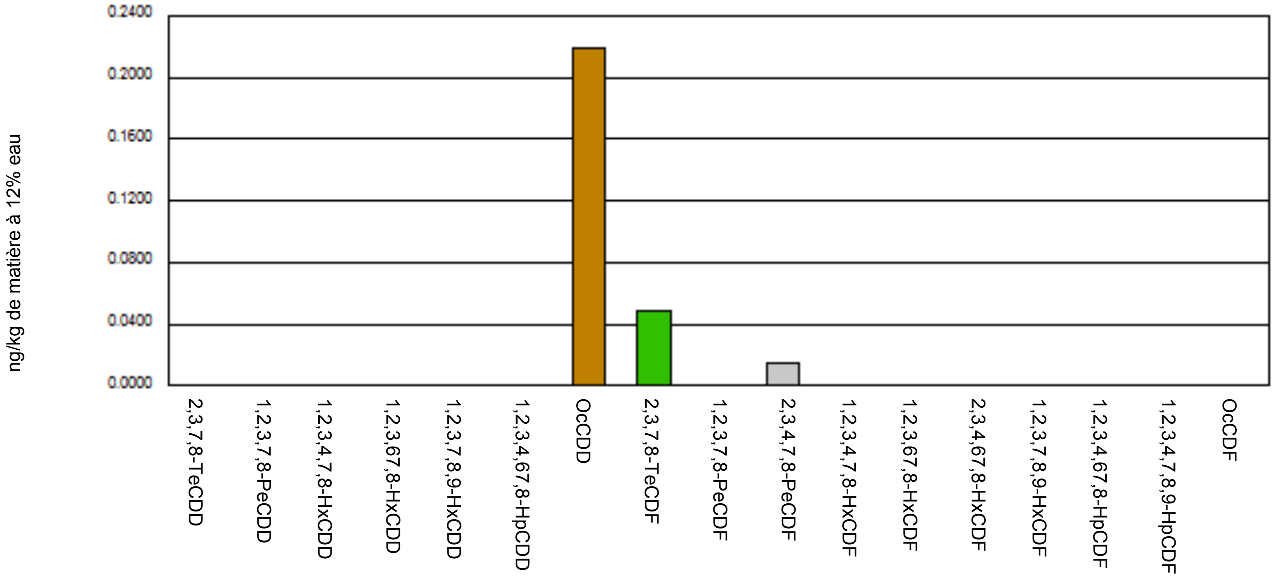

PCDD/F-TEQ des 17 congénères toxiques

Référence CARSO-LSEHL : LSE1708-48971

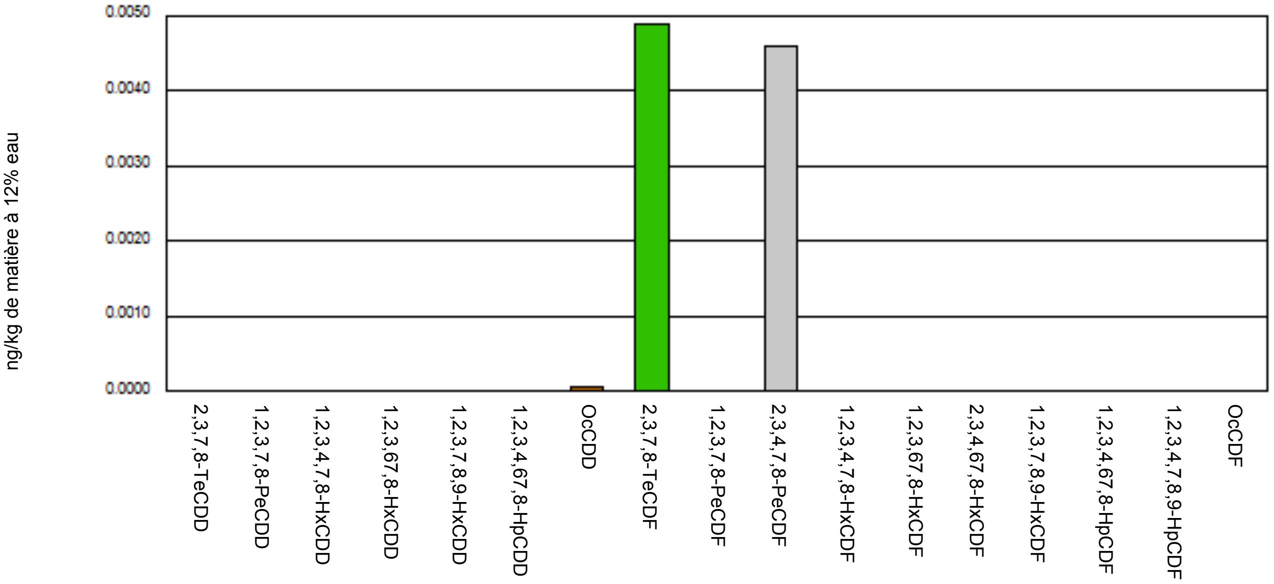

Société par action simplifiée au capital de 2 283 622,30 € - RCS Lyon B 410 545 313 - SIRET 410 545 313 00042 - APE 7120B — N° TVA: FR 82 [illegible]
Siège social et laboratoire : 4, avenue Jean Moulin — CS 30228 - 69633 VENISSIEUX CEDEX - Tél : (33) 04 72 76 16 16 - Fax : (33) 04 78 72 35 03
Site web : www.groupecarso.com - e-mail : carso@groupecarso.com

Institut Scientifique d'Hygiène et d'Analyse
S.A.S au capital de 4 100 000 euros - SIREN : 489 268 045 - APE : 7022 Z
N° TVA intracommunautaire : FR50 489 268 045 - RCS : EVRY 489 268 045
Rue du Chemin Blanc - CHAMPLAN - F-91165 LONGJUMEAU
Tél : 01 69 79 31 50 - Fax : 01 64 48 82 49

Un laboratoire du Groupe Alpa

N° Dossier : MIB.91610
Code client : 568020600
V/ref. : U8220 V257 LOT: 17207/17209
Date commande : 10/08/2017
Date réception : 16/08/2017

SAFE
Monsieur BARRAL RENAUD
ROUTE DE SAINT BRIS
89290 AUGY

RAPPORT D'ANALYSE

Champlan le 24/08/2017

Echantillon : K 9187 (MIB.91610.1)

Libellé : U8220 V257 CD011344 ENVOI E8795 - REALISER 1 ECH MOYEN

Date de prélèvement :
Lieu de prélèvement :
Température produit :
Date préparation :
Fournisseur :
Date livraison :
Date mise en analyse : 17/08/2017

Fabriquant :
Date Fabr. :
DLC :
N° de lot : 17207
N° agrément :
Grammage : ENV.250G
Code article :
Conditionnement : 2 POTS
Mode conservation :

Analyse	Méthode	Résultat	Critère	App.	Réf	H/S
* **Microorganismes aérobies 30°C**	NF EN ISO 4833-1	**< 10 / g**	100000	S	Critère demandeur	H
**Levures**	Méthode interne (selon NF V08-059)	**< 100 / g**	1000	S	Critère demandeur	H
**Moisissures**	Méthode interne (selon NF V08-059)	**< 100 / g**	1000	S	Critère demandeur	H
* **Escherichia coli ß-glucuronidase +**	NF ISO 16649-2	**< 1 / g**				
**Staphylocoques à coagulase positive**	NF EN ISO 6888-3	**ABSENCE / 1 g**				
* **Anaérobies sulfito-réducteurs 46°C (boîtes)**	NF V 08-061	**< 10 / g**	100	S	Critère demandeur	H
* **Clostridium perfringens**	NF EN ISO 7937	**< 1 / g**				
**Clostridium perfringens (spores)**	ISHA / CPPRC	**< 1 / g**				
* **Bacillus cereus présomptifs**	NF EN ISO 7932	**< 10 / g**				
**Bacillus cereus présomptifs (spores)**	ISHA / BCPRC	**< 10 / g**				
* **Salmonella (recherche)**	RBP 31/01-06/08	**ABSENCE / 25 g**	ABSENCE	S	Critère demandeur	H
* **Listeria monocytogenes (recherche)**	AFNOR AES-10/3-09/00	**ABSENCE / 25 g**				
* **Entérobactéries 30°C présumées**	NF V 08-054	**< 10 / g**				
**Pseudomonas**	ISHA / PSENA	**< 10 / g**				

Stéphanie MAHLER
Responsable d'unité microbiologie

ACCREDITATION N° 1 -1759 PORTEE DISPONIBLE SUR www.cofrac.fr
cofrac ESSAIS

Ce rapport d'analyse ne concerne que les objets soumis aux analyses. Sa reproduction n'est autorisée que sous forme de fac-similé photographique intégral.
Seuls certains essais rapportés dans ce document sont couverts par l'accréditation de la Section Laboratoire du Cofrac. Ils sont identifiés par le symbole *. Ce rapport comporte 4 pages.

Institut Scientifique d'Hygiène et d'Analyse
S.A.S au capital de 4 100 000 euros - SIREN : 489 268 045 - APE : 7022 Z
N° TVA intracommunautaire : FR50 489 268 045 - RCS : EVRY 489 268 045
Rue du Chemin Blanc - CHAMPLAN - F-91165 LONGJUMEAU
Tél : 01 69 79 31 50 - Fax : 01 64 48 82 49

ISHA
Un laboratoire du Groupe Alpa

N° Dossier : MIB.91610
Code client : 568020600
V/ref. : U8220 V257 LOT: 17207/17209
Date commande : 10/08/2017
Date réception : 16/08/2017

SAFE
Monsieur BARRAL RENAUD
ROUTE DE SAINT BRIS
89290 AUGY

RAPPORT D'ANALYSE

Champlan le 24/08/2017

Appréciation: S=Satisfaisant - A= Acceptable - I=Insatisfaisant / H/S: S=critère de sécurité - H=critère d'hygiène des procédés

Le dossier comporte 2 échantillons

* Analyse accréditée COFRAC. Lorsque tous les paramètres analytiques concernés par la déclaration de conformité sont réalisés sous accréditation, la conclusion globale de conformité est par défaut sous accréditation. La déclaration de conformité ne tient pas compte de l'incertitude de mesure.

Conclusion critères de sécurité : *Absence de critères*

Conclusion hygiène des procédés : SATISFAISANT

Stéphanie MAHLER

Responsable d’unité microbiologie

ACCREDITATION N° 1 -1759 PORTEE DISPONIBLE SUR www.cofrac.fr

cofrac ESSAIS

Ce rapport d'analyse ne concerne que les objets soumis aux analyses. Sa reproduction n'est autorisée que sous forme de fac-similé photographique intégral.
Seuls certains essais rapportés dans ce document sont couverts par l'accréditation de la Section Laboratoire du Cofrac. Ils sont identifiés par le symbole *. Ce rapport comporte 4 pages.

Institut Scientifique d'Hygiène et d'Analyse
S.A.S au capital de 4 100 000 euros - SIREN : 489 268 045 - APE : 7022 Z
N° TVA intracommunautaire : FR50 489 268 045 - RCS : EVRY 489 268 045
Rue du Chemin Blanc - CHAMPLAN - F-91165 LONGJUMEAU
Tél : 01 69 79 31 50 - Fax : 01 64 48 82 49

Un laboratoire du Groupe Alpa

N° Dossier : MIB.91610
Code client : 568020600
V/ref. : U8220 V257 LOT: 17207/17209
Date commande : 10/08/2017
Date réception : 16/08/2017

SAFE
Monsieur BARRAL RENAUD
ROUTE DE SAINT BRIS
89290 AUGY

RAPPORT D'ANALYSE

Champlan le 24/08/2017

Echantillon : K 9188 (MIB.91610.2)

Libellé : U8220 V257 MP ORGANIQUE CD011344 ENVOI E8796 - REALISER 1 ECH MOYEN

Date de prélèvement :
Lieu de prélèvement :
Température produit :
Date préparation :
Fournisseur :
Date livraison :
Date mise en analyse : 17/08/2017

Fabriquant :
Date Fabr. :
DLC :
N° de lot : 17209
N° agrément :
Grammage : ENV.250G
Code article :
Conditionnement : 2 POTS
Mode conservation :

Analyse	Méthode	Résultat	Critère	App.	Réf	H/S
* **Microorganismes aérobies 30°C**	NF EN ISO 4833-1	**< 10 / g**	100000	S	Critère demandeur	H
**Levures**	Méthode interne (selon NF V08-059)	**< 100 / g**	1000	S	Critère demandeur	H
**Moisissures**	Méthode interne (selon NF V08-059)	**< 100 / g**	1000	S	Critère demandeur	H
* **Escherichia coli ß-glucuronidase +**	NF ISO 16649-2	**< 1 / g**				
**Staphylocoques à coagulase positive**	NF EN ISO 6888-3	**ABSENCE / 1 g**				
* **Anaérobies sulfito-réducteurs 46°C (boîtes)**	NF V 08-061	**< 10 / g**	100	S	Critère demandeur	H
* **Clostridium perfringens**	NF EN ISO 7937	**< 1 / g**				
**Clostridium perfringens (spores)**	ISHA / CPPRC	**< 1 / g**				
* **Bacillus cereus présomptifs**	NF EN ISO 7932	**< 10 / g**				
**Bacillus cereus présomptifs (spores)**	ISHA / BCPRC	**< 10 / g**				
* **Salmonella (recherche)**	RBP 31/01-06/08	**ABSENCE / 25 g**	ABSENCE	S	Critère demandeur	H
* **Listeria monocytogenes (recherche)**	AFNOR AES-10/3-09/00	**ABSENCE / 25 g**				
* **Entérobactéries 30°C présumées**	NF V 08-054	**< 10 / g**				
**Pseudomonas**	ISHA / PSENA	**< 10 / g**				

Stéphanie MAHLER
Responsable d'unité microbiologie

ACCREDITATION N° 1 -1759 PORTEE DISPONIBLE SUR www.cofrac.fr
cofrac ESSAIS

Ce rapport d'analyse ne concerne que les objets soumis aux analyses. Sa reproduction n'est autorisée que sous forme de fac-similé photographique intégral.
Seuls certains essais rapportés dans ce document sont couverts par l'accréditation de la Section Laboratoire du Cofrac. Ils sont identifiés par le symbole *. Ce rapport comporte 4 pages.

Institut Scientifique d'Hygiène et d'Analyse
S.A.S au capital de 4 100 000 euros - SIREN : 489 268 045 - APE : 7022 Z
N° TVA intracommunautaire : FR50 489 268 045 - RCS : EVRY 489 268 045
Rue du Chemin Blanc - CHAMPLAN - F-91165 LONGJUMEAU
Tél : 01 69 79 31 50 - Fax : 01 64 48 82 49

ISHA
Un laboratoire du Groupe Alpa

N° Dossier : MIB.91610
Code client : 568020600
V/ref. : U8220 V257 LOT: 17207/17209
Date commande : 10/08/2017
Date réception : 16/08/2017

SAFE
Monsieur BARRAL RENAUD
ROUTE DE SAINT BRIS
89290 AUGY

RAPPORT D'ANALYSE

Champlan le 24/08/2017

Appréciation: S=Satisfaisant - A= Acceptable - I=Insatisfaisant / H/S: S=critère de sécurité - H=critère d'hygiène des procédés

Le dossier comporte 2 échantillons

* Analyse accréditée COFRAC. Lorsque tous les paramètres analytiques concernés par la déclaration de conformité sont réalisés sous accréditation, la conclusion globale de conformité est par défaut sous accréditation. La déclaration de conformité ne tient pas compte de l'incertitude de mesure.

Conclusion critères de sécurité : *Absence de critères*

Conclusion hygiène des procédés : SATISFAISANT

Stéphanie MAHLER
Responsable d'unité microbiologie

ACCREDITATION
N° 1 -1759
PORTEE
DISPONIBLE SUR
www.cofrac.fr

Ce rapport d'analyse ne concerne que les objets soumis aux analyses. Sa reproduction n'est autorisée que sous forme de fac-similé photographique intégral.
Seuls certains essais rapportés dans ce document sont couverts par l'accréditation de la Section Laboratoire du Cofrac. Ils sont identifiés par le symbole *. Ce rapport comporte 4 pages.

Essai(s) réalisé(s) à l'ISHA : 25, avenue de la république 91300 Massy

SIGMA-ALDRICH

3050 Spruce Street, Saint Louis, MO 63103 USA
Email USA: techserv@sial.com Outside USA: eurtechserv@sial.com

Certificate of Analysis

**Product Name:**	AZOXYSTROBIN<br>PESTANAL™, analytical standard
**Product Number:**	31697
**Batch Number:**	BCBT1118V
**Brand:**	Sigma-Aldrich
**CAS Number:**	131860-33-8
**Formula:**	$C_{22}H_{17}N_3O_5$
**Formula Weight:**	403.39
**Expiration Date:**	OCT 2021
**Quality Release Date:**	04 NOV 2016

TEST	SPECIFICATION	RESULT
APPEARANCE (COLOR)	WHITE TO YELLOW AND FAINT BEIGE TO LIGHT BEIGE AND FAINT BROWN TO LIGHT BROWN	YELLOW
APPEARANCE (FORM)	POWDER OR CRYSTALS	POWDER
PURITY (HPLC AREA %)	≥ 98.0 %	99.3 %
MELTING POINT	114 - 120 C	117 C
WATER	≤ 1.0 %	< 0.05%
PROTON NMR SPECTRUM	CONFORMS TO STRUCTURE	CONFORMS

Claudia Geitner

Dr. Claudia Geitner
Manager Quality Control
Buchs, Switzerland

Sigma-Aldrich warrants that at the time of the quality release or subsequent retest date this product conformed to the information contained in this publication. The current specification sheet may be available at Sigma-Aldrich.com. For further inquiries, please contact Technical Service. Purchaser must determine the suitability of the product for its particular use. See reverse side of invoice or packing slip for additional terms and conditions of sale.

31697 Azoxystrobin

Lot Number: **BCBT1118V**	Sample Name: **T38864_001**

Dionex Ultimate 3000 RSLC

Pump :	LPG-3400RS	***Injection Time:***	31.10.16 14:26
Autosampler:	WPS-3000	***Processed By:***	Mikaël Berthet
Detector:	VWD-3400	***Vial Number:***	BB4
Column:	Ascentis Express C18, 2.7 um	***Column S/N:***	USWM003002
Column Dim.:	150 x 2.1 mm	***Sample Type:***	unknown
Mobile Phase:		***Injection Volume:***	1.0 µl
%A :	Acetonitrile	***Flow:***	0.50 ml/min
%B :	Methanol	***Column Temp. (°C):***	35.0
%C :	Water	***Run Time:***	15.00 min
%D :	Phosphoric acid 0.01M		
Gradient :	see Figure 1		
Sample Prep.:	2 mg sample in 10 ml acetonitrile		

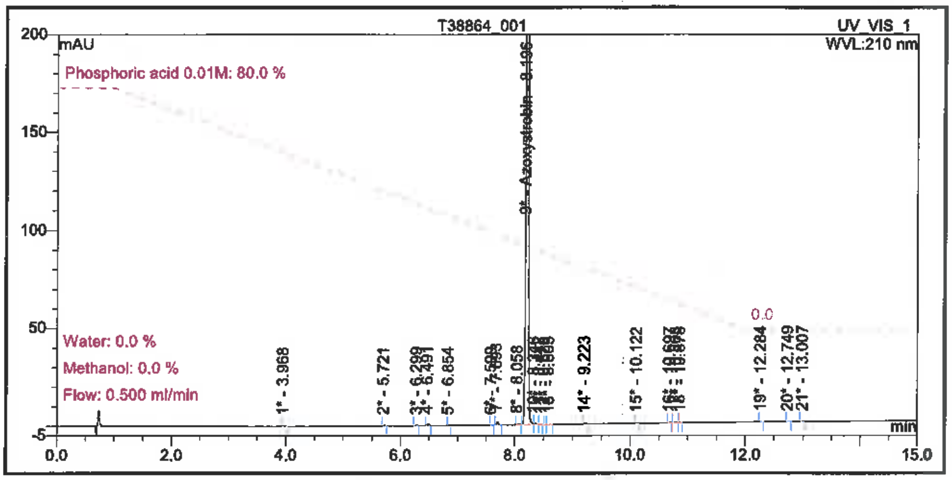

Figure 1: Zoomed Chromatogram

No.	Ret.Time min	Peak Name	Area mAU*min	Height mAU	Amount	Rel.Area %
1	3.968	n.a.	0.01720	0.33851	n.a.	0.05
2	5.721	n.a.	0.00897	0.29648	n.a.	0.03
3	6.299	n.a.	0.00575	0.16333	n.a.	0.02
4	6.491	n.a.	0.02789	0.81099	n.a.	0.08
5	6.854	n.a.	0.00260	0.07859	n.a.	0.01
6	7.599	n.a.	0.00428	0.11493	n.a.	0.01
7	7.693	n.a.	0.05994	1.74606	n.a.	0.18
8	8.058	n.a.	0.01498	0.44592	n.a.	0.04
9	8.196	Azoxystrobin	33.19362	967.34672	n.a.	99.31
10	8.346	n.a.	0.02302	0.46552	n.a.	0.07
11	8.442	n.a.	0.00627	0.18528	n.a.	0.02
12	8.528	n.a.	0.00458	0.14295	n.a.	0.01
13	8.605	n.a.	0.01373	0.31659	n.a.	0.04
14	9.223	n.a.	0.00937	0.28877	n.a.	0.03
15	10.122	n.a.	0.00806	0.22493	n.a.	0.02
16	10.697	n.a.	0.00610	0.13251	n.a.	0.02
17	10.808	n.a.	0.00529	0.10469	n.a.	0.02
18	10.878	n.a.	0.00266	0.08675	n.a.	0.01
19	12.284	n.a.	0.00456	0.12120	n.a.	0.01
20	12.749	n.a.	0.00402	0.08758	n.a.	0.01
21	13.007	n.a.	0.00240	0.07375	n.a.	0.01
Total:			33.42528	973.57205		100.00

Table 1: Integration

SIGMA-ALDRICH®

3050 Spruce Street, Saint Louis, MO 63103 USA
Email USA: techserv@sial.com Outside USA: eurtechserv@sial.com

Certificate of Analysis

**Product Name:**	BOSCALID<br>PESTANAL™, analytical standard
**Product Number:**	33875
**Batch Number:**	BCBS8868V
**Brand:**	Sigma-Aldrich
**CAS Number:**	188425-85-6
**Formula:**	$C_{18}H_{12}Cl_2N_2O$
**Formula Weight:**	343.21
**Expiration Date:**	AUG 2021
**Quality Release Date:**	16 SEP 2016

TEST	SPECIFICATION	RESULT
APPEARANCE (COLOR)	WHITE TO LIGHT YELLOW AND FAINT BEIGE TO LIGHT BEIGE	FAINT YELLOW
APPEARANCE (FORM)	POWDER OR CRYSTALS	POWDER
PURITY (HPLC AREA %)	≥ 98.0 %	99.5 %
MELTING POINT	145 - 150 C	145 C
WATER	≤ 1.0 %	0.49 %
PROTON NMR SPECTRUM	CONFORMS TO STRUCTURE	CONFORMS

Claudia Geitner

Dr. Claudia Geitner
Manager Quality Control
Buchs, Switzerland

Sigma-Aldrich warrants that at the time of the quality release or subsequent retest date this product conformed to the information contained in this publication. The current specification sheet may be available at Sigma-Aldrich.com. For further inquiries, please contact Technical Service. Purchaser must determine the suitability of the product for its particular use. See reverse side of invoice or packing slip for additional terms and conditions of sale.

33875 Boscalid

Lot Number: **BCBS8868V**	Sample Name: **00000000560714_001_LC**

Dionex Ultimate 3000 RSLC

Pump :	LPG-3400RS	***Injection Time:***	01.09.16 17:59
Autosampler:	WPS-3000	***Processed By:***	Mikaël Berthet
Detector:	DAD-3000RS	***Vial Number:***	RB6
Column:	Supelco Ascentis Express C18, 2.7 um	***Column S/N:***	-
Column Dim.:	50 x 2.1 mm	***Sample Type:***	unknown
Mobile Phase:		***Injection Volume:***	0.5 µl
%A :	Acetonitrile	***Flow:***	1.00 ml/min
%B :	Methanol	***Column Temp. (°C):***	35.0
%C :	Water	***Run Time:***	6.00 min
%D :	H3PO4 0.01 M		
Gradient :	see Figure 1		
Sample Prep.:	0.6 mg sample in 1ml **acetonitrile:water 1:1**		

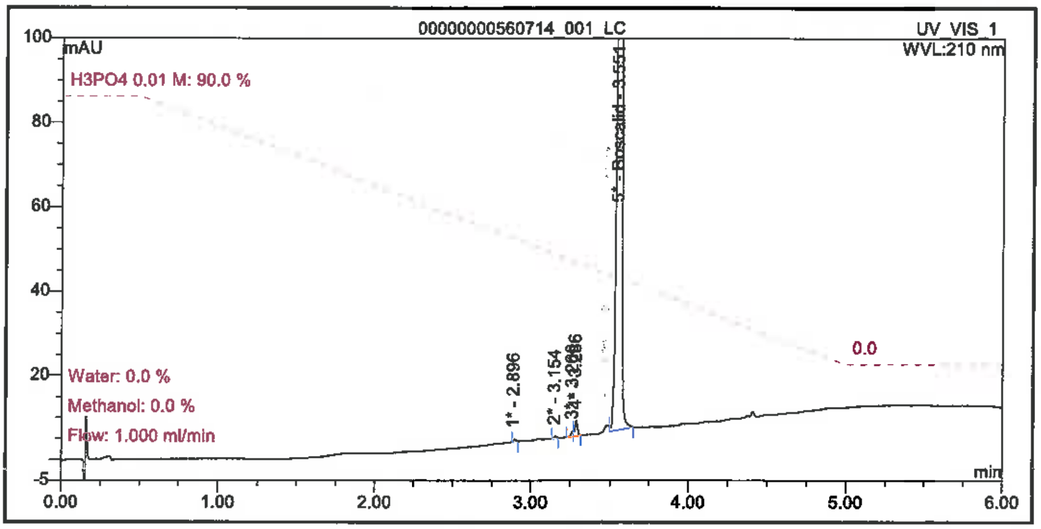

Figure 1: Zoomed Chromatogram

No.	Ret.Time min	Peak Name	Area mAU*min	Height mAU	Amount	Rel.Area %
1	2.896	n.a.	0.01173	0.59038	n.a.	0.05
2	3.154	n.a.	0.00898	0.50012	n.a.	0.04
3	3.266	n.a.	0.02053	1.22821	n.a.	0.09
4	3.286	n.a.	0.06899	3.56225	n.a.	0.31
5	3.551	Boscalid	21.99856	1091.96482	n.a.	99.50
Total:			22.10881	1097.84578		100.00

Table 1: Integration

SIGMA-ALDRICH®

3050 Spruce Street, Saint Louis, MO 63103 USA
Email USA: techserv@sial.com Outside USA: eurtechserv@sial.com

Certificate of Analysis

**Product Name:**	CHLORPYRIFOS<br>PESTANAL™, analytical standard
**Product Number:**	45395
**Batch Number:**	BCBR6591V
**Brand:**	Sigma-Aldrich
**CAS Number:**	2921-88-2
**Formula:**	$C_9H_{11}Cl_3NO_3PS$
**Formula Weight:**	350.59
**Storage Temperature:**	2-8 C
**Expiration Date:**	JAN 2021
**Quality Release Date:**	02 MAR 2016

TEST	SPECIFICATION	RESULT
APPEARANCE (COLOR)	WHITE TO LIGHT YELLOW AND FAINT BEIGE TO LIGHT BEIGE	LIGHT YELLOW
APPEARANCE (FORM)	POWDER OR CRYSTALS OR FLAKES OR CHUNKS	SOLID
PURITY (HPLC AREA %)	≥ 98.0 %	99.3%
MELTING POINT	40 - 46 C	42C
WATER	≤ 1.0 %	0.42%
PROTON NMR SPECTRUM	CONFORMS TO STRUCTURE	CONFORMS

Claudia Geitner

Dr. Claudia Geitner
Manager Quality Control
Buchs, Switzerland

Sigma-Aldrich warrants that at the time of the quality release or subsequent retest date this product conformed to the information contained in this publication. The current specification sheet may be available at Sigma-Aldrich.com. For further inquiries, please contact Technical Service. Purchaser must determine the suitability of the product for its particular use. See reverse side of invoice or packing slip for additional terms and conditions of sale.

45395 Chlorpyriphos

Lot Number: **BCBR6591V**	Sample Name: **00000000541513_001_LC**

Dionex Ultimate 3000 RSLC

Pump :	LPG-3400RS	***Injection Time:***	26.01.16 16:36
Autosampler:	WPS-3000	***Processed By:***	Mikaël Berthet
Detector:	DAD-3000RS	***Vial Number:***	GA3
Column:	Supelco Ascentis Express C18, 2.7 um	***Column S/N:***	USMD003672
Column Dim.:	50 x 2.1 mm	***Sample Type:***	unknown
Mobile Phase:		***Injection Volume:***	2.0 µl
%A :	Acetonitrile	***Flow:***	1.00 ml/min
%B :	Methanol	***Column Temp. (°C):***	35.0
%C :	Water	***Run Time:***	6.00 min
%D :	Phosphoric acid 0.01 M		
Gradient :	see Figure 1		
Sample Prep.:	1 mg sample in 1 ml acetonitrile:water 1:1		

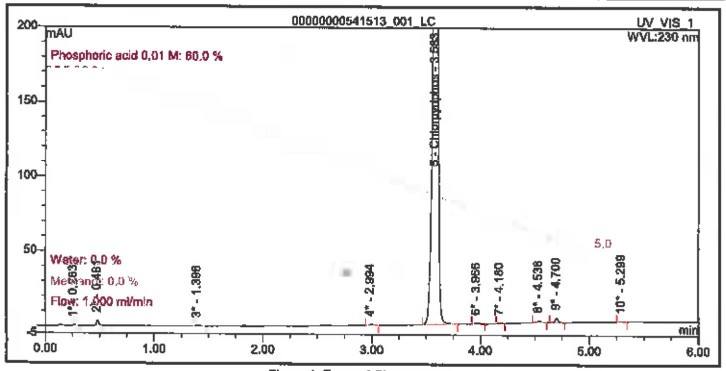

Figure 1: Zoomed Chromatogram

No.	Ret.Time min	Peak Name	Area mAU*min	Height mAU	Amount	Rel.Area %
1	0.263	n.a.	0.00400	0.25277	n.a.	0.01
2	0.481	n.a.	0.07993	3.59690	n.a.	0.19
3	1.398	n.a.	0.01017	0.25222	n.a.	0.02
4	2.994	n.a.	0.02940	0.82896	n.a.	0.07
5	3.583	Chlorpyriphos	42.78613	1276.42848	n.a.	99.31
6	3.966	n.a.	0.02356	0.61797	n.a.	0.05
7	4.180	n.a.	0.01137	0.35900	n.a.	0.03
8	4.538	n.a.	0.03659	1.27015	n.a.	0.08
9	4.700	n.a.	0.09201	3.04022	n.a.	0.21
10	5.299	n.a.	0.00815	0.27754	n.a.	0.02
Total:			43.08130	1286.92421		100.00

Table 1: Integration

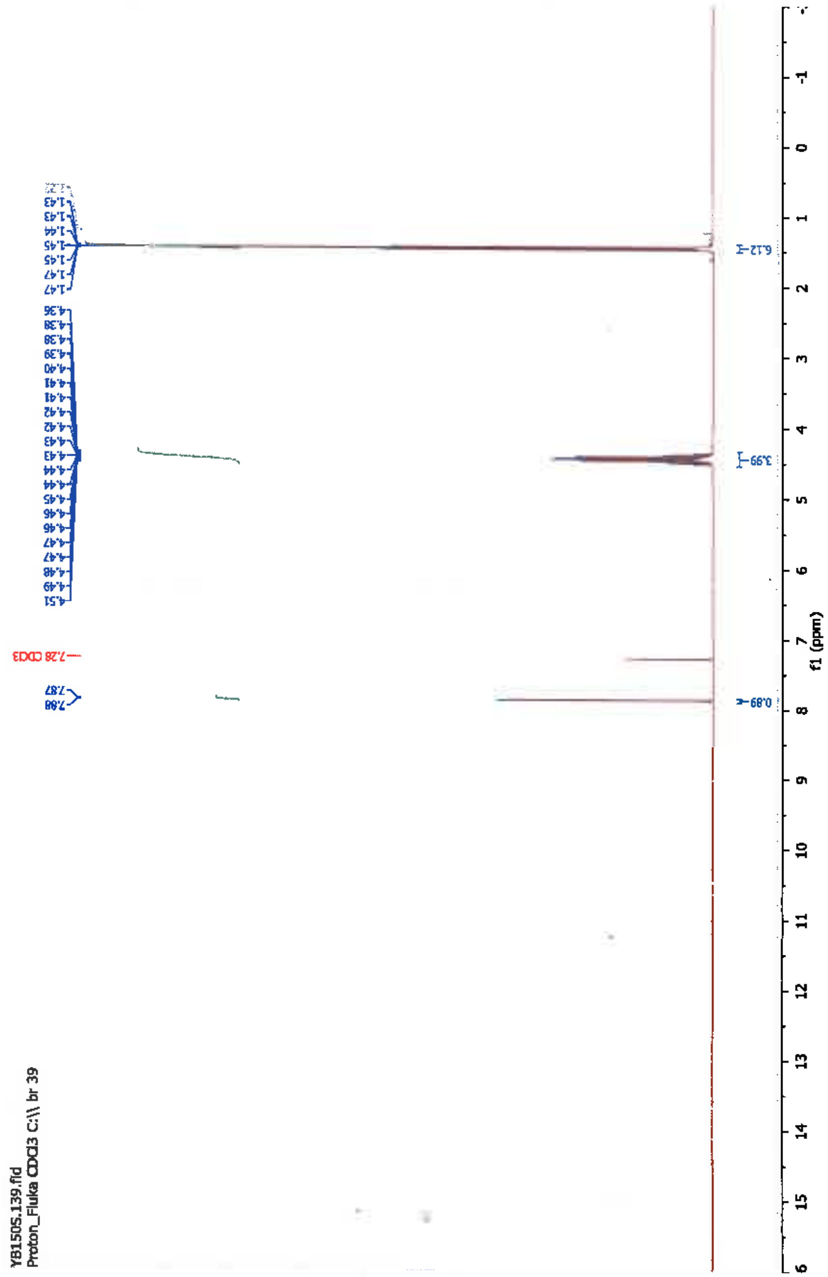

SIGMA-ALDRICH

sigma-aldrich.com

3050 Spruce Street, Saint Louis, MO 63103, USA
Website: www.sigmaaldrich.com
Email USA: techserv@sial.com
Outside USA: eurtechserv@sial.com

Certificate of Analysis

Product Name:
N-(Phosphonomethyl)glycine - 96%

**Product Number:**	**337757**
**Batch Number:**	**MKCG2949**
Brand:	ALDRICH
CAS Number:	1071-83-6
MDL Number:	MFCD00055350
Formula:	C3H8NO5P
Formula Weight:	169.07 g/mol
Quality Release Date:	24 APR 2018

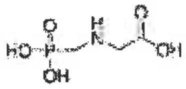

Test	Specification	Result
Appearance (Color)	White	White
Appearance (Form)	Powder	Powder
Infrared Spectrum	Conforms to Structure	Conforms
Carbon	20.3 - 22.3 %	21.3 %
Nitrogen	7.9 - 8.7 %	8.2 %
Purity (TLC)	$\geq$ 96 %	> 99 %
Solubility (Turbidity) 5% in 1N NAOH	Clear to Slightly Hazy	Clear
Solubility (Color)	Colorless to Faint Yellow	Colorless

Michael Grady, Manager
Quality Control
Milwaukee, WI US

Sigma-Aldrich warrants, that at the time of the quality release or subsequent retest date this product conformed to the information contained in this publication. The current Specification sheet may be available at Sigma-Aldrich.com. For further inquiries, please contact Technical Service. Purchaser must determine the suitability of the product for its particular use. See reverse side of invoice or packing slip for additional terms and conditions of sale.

Version Number: 1

SIGMA-ALDRICH®

3050 Spruce Street, Saint Louis, MO 63103 USA
Email USA: techserv@sial.com Outside USA: eurtechserv@sial.com

Certificate of Analysis

**Product Name:**	IMIDACLOPRID<br>PESTANAL™, analytical standard
**Product Number:**	37894
**Batch Number:**	BCBT2267
**Brand:**	Sigma-Aldrich
**CAS Number:**	138261-41-3
**Formula:**	$C_9H_{10}ClN_5O_2$
**Formula Weight:**	255.66
**Expiration Date:**	OCT 2021
**Quality Release Date:**	22 NOV 2016

TEST	SPECIFICATION	RESULT
APPEARANCE (COLOR)	WHITE TO LIGHT YELLOW AND FAINT BEIGE TO LIGHT BEIGE	WHITE
APPEARANCE (FORM)	POWDER OR CRYSTALS	POWDER
PURITY (HPLC AREA %)	≥ 98.0 %	100.0 %
MELTING POINT	141 - 146 C	145 C
WATER	≤ 1.0 %	0.54 %
PROTON NMR SPECTRUM	CONFORMS TO STRUCTURE	CONFORMS

Dr. Claudia Geitner
Manager Quality Control
Buchs, Switzerland

Sigma-Aldrich warrants that at the time of the quality release or subsequent retest date this product conformed to the information contained in this publication. The current specification sheet may be available at Sigma-Aldrich.com. For further inquiries, please contact Technical Service. Purchaser must determine the suitability of the product for its particular use. See reverse side of invoice or packing slip for additional terms and conditions of sale.

37894 Imidacloprid

Lot Number: **BCBT2267**	Sample Name: **T38933_001_LC**

Dionex Ultimate 3000 RSLC

Pump :	LPG-3400RS	***Injection Time:***	18.11.16 11:17
Autosampler:	WPS-3000	***Processed By:***	Markus Urthaler
Detector:	VWD-3400	***Vial Number:***	BA2
Column:	Supelco Ascentis Express C18, 2.7 um	***Column S/N:***	-
Column Dim.:	50 x 2.1 mm	***Sample Type:***	unknown
Mobile Phase:		***Injection Volume:***	1.0 µl
%A :	Acetonitrile	***Flow:***	1.00 ml/min
%B :	Methanol	***Column Temp. (°C):***	35.0
%C :	Water	***Run Time:***	6.00 min
%D :	Phosphoric acid 0.01M		
Gradient :	see Figure 1		
Sample Prep.:	0.5 mg sample in 1ml methanol		

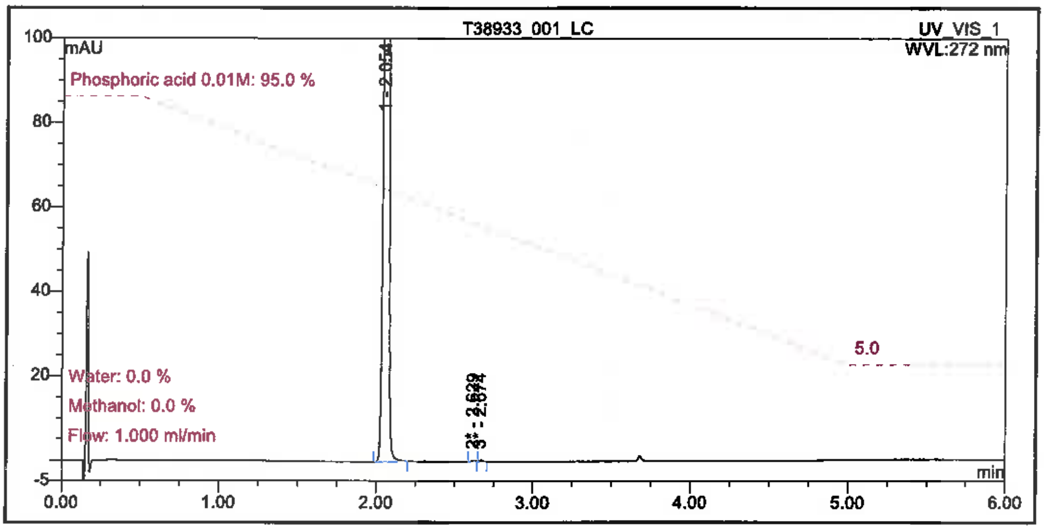

Figure 1: Zoomed Chromatogram

No.	Ret.Time min	Peak Name	Area mAU*min	Height mAU	Amount	Rel.Area %
1	2.054	n.a.	30.01747	1460.18351	n.a.	99.95
2	2.629	n.a.	0.00738	0.31070	n.a.	0.02
3	2.674	n.a.	0.00750	0.40892	n.a.	0.02
Total:			30.03234	1460.90313		100.00

Table 1: Integration

SIGMA-ALDRICH

3050 Spruce Street, Saint Louis, MO 63103 USA
Email USA: techserv@sial.com Outside USA: eurtechserv@sial.com

Certificate of Analysis

**Product Name:**	THIABENDAZOLE<br>PESTANAL™, analytical standard
**Product Number:**	45684
**Batch Number:**	BCBV5436
**Brand:**	Sigma-Aldrich
**CAS Number:**	148-79-8
**Formula:**	$C_{10}H_7N_3S$
**Formula Weight:**	201.25
**Expiration Date:**	JUL 2022
**Quality Release Date:**	08 AUG 2017

TEST	SPECIFICATION	RESULT
APPEARANCE (COLOR)	WHITE TO OFF WHITE	WHITE
APPEARANCE (FORM)	POWDER OR CRYSTALS	POWDER
PURITY (GC AREA %)	≥ 98.0 %	98.6 %
MELTING POINT	298 - 304 C	302 C
WATER	≤ 1.0 %	0.09 %
PROTON NMR SPECTRUM	CONFORMS TO STRUCTURE	CONFORMS

Dr. Claudia Geitner
Manager Quality Control
Buchs, Switzerland

Sigma-Aldrich warrants that at the time of the quality release or subsequent retest date this product conformed to the information contained in this publication. The current specification sheet may be available at Sigma-Aldrich.com. For further inquiries, please contact Technical Service. Purchaser must determine the suitability of the product for its particular use. See reverse side of invoice or packing slip for additional terms and conditions of sale.

45684 Thiabendazole

Lot Number:	BCBV5436	Sample Name:	T39427_001_GC

Shimadzu GC-2010 Plus

Injector :	250 °C	*GC Serial Number:*	C11805270206 US
Detector (FID):	320 °C	*Injection Time:*	25.07.17 17:02
Carriergas:	Helium	*Sample Type:*	unknown
Velocity:	40.0 cm/s	*Channel:*	GC_2
Temp. Control:	see Figure 1	*Sequence Creation Time:*	04.07.17 15:51
Split Ratio:	100:1	*Sequence Created By:*	Mirela Brdanovic
Range:	-	*Time Processed:*	11.08.17 09:14
Injection Volume:	1.0 ul	*Processed By:*	Markus Urthaler
Vial Number:	4		
Run Time:	20.00 min		
Column:	Equity-1, 30 m x 0.25 mm, 1.0 um		
Sample Prep.:	ca 20mg sample in 2ml methanol		

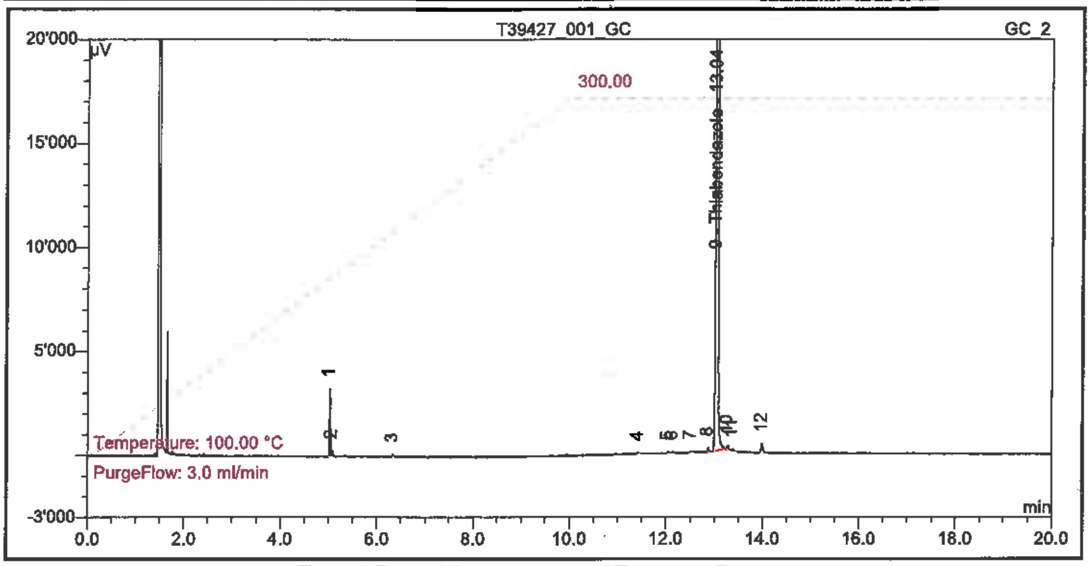

Figure 1: Zoomed Chromatogram and Temperatur Program

No.	Ret.Time min	Peak Name	Type	Height μV	Area μV*min	Amount	Rel.Area %
1	5.028	n.a.	BM	3257	59.4046	n.a.	0.714
2	5.074	n.a.	MB	269	5.5508	n.a.	0.067
3	6.325	n.a.	BMB	115	1.7783	n.a.	0.021
4	11.430	n.a.	BMB*	80	2.8125	n.a.	0.034
5	12.042	n.a.	BMB*	76	1.9471	n.a.	0.023
6	12.143	n.a.	BMB*	88	3.4967	n.a.	0.042
7	12.516	n.a.	BMB*	83	3.0967	n.a.	0.037
8	12.871	n.a.	BMB*	210	7.2075	n.a.	0.087
9	13.040	Thiabendazole	BMB	245305	8210.3329	n.a.	98.619
10	13.274	n.a.	BMB	204	6.9992	n.a.	0.084
11	13.377	n.a.	BMB*	108	6.3367	n.a.	0.076
12	13.988	n.a.	BMB	423	16.3450	n.a.	0.196
Total:				250218	8325.3079		100.000
