# Supplementary material for: Multi-omics phenotyping of the gut-liver axis reveals metabolic perturbations from a low-dose pesticide mixture in rats
Source: Commun Biol. 2021 Apr 14;4:471. doi: 10.1038/s42003-021-01990-w (PMC8046807; doi:10.1038/s42003-021-01990-w)
Supplement: Supplementary file 6 — Supplementary Data 5 [file 42003_2021_1990_MOESM6_ESM.pdf]

COMMSBIO pesticide mixture toxicity

Load the packages used to make this statistical analysis

```
library(qvalue)
library(dplyr)
```

```
##
## Attaching package: 'dplyr'
```

```
## The following objects are masked from 'package:stats':
##
##     filter, lag
```

```
## The following objects are masked from 'package:base':
##
##     intersect, setdiff, setequal, union
```

```
library(ggplot2)
library(ropls)
```

```
## Loading required package: Biobase
```

```
## Loading required package: BiocGenerics
```

```
## Loading required package: parallel
```

```
##
## Attaching package: 'BiocGenerics'
```

```
## The following objects are masked from 'package:parallel':
##
##     clusterApply, clusterApplyLB, clusterCall, clusterEvalQ,
##     clusterExport, clusterMap, parApply, parCapply, parLapply,
##     parLapplyLB, parRapply, parSapply, parSapplyLB
```

```
## The following objects are masked from 'package:dplyr':
##
##     combine, intersect, setdiff, union
```

```
## The following objects are masked from 'package:stats':
##
##     IQR, mad, sd, var, xtabs
```

```
## The following objects are masked from 'package:base':
##
##     anyDuplicated, append, as.data.frame, basename, cbind, colnames,
##     dirname, do.call, duplicated, eval, evalq, Filter, Find, get, grep,
##     grepl, intersect, is.unsorted, lapply, Map, mapply, match, mget,
##     order, paste, pmax, pmax.int, pmin, pmin.int, Position, rank,
##     rbind, Reduce, rownames, sapply, setdiff, sort, table, tapply,
##     union, unique, unsplit, which, which.max, which.min
```

```
## Welcome to Bioconductor
##
##     Vignettes contain introductory material; view with
##     'browseVignettes()'. To cite Bioconductor, see
##     'citation("Biobase")', and for packages 'citation("pkgname")'.
```

```
library(readxl)
library(ALDEx2)
```

```
## Loading required package: zCompositions
```

```
## Loading required package: MASS
```

```
##
## Attaching package: 'MASS'
```

```
## The following object is masked from 'package:dplyr':
##
##     select
```

```
## Loading required package: NADA
```

```
## Loading required package: survival
```

```
##
## Attaching package: 'NADA'
```

```
## The following object is masked from 'package:stats':
##
##     cor
```

```
## Loading required package: truncnorm
```

```
library(tximport)
library(GenomicFeatures)
```

```
## Loading required package: S4Vectors
```

```
## Loading required package: stats4
```

```
##
## Attaching package: 'S4Vectors'
```

```
## The following objects are masked from 'package:dplyr':
##
##     first, rename
```

```
## The following object is masked from 'package:base':
##
##     expand.grid
```

```
## Loading required package: IRanges
```

```
##
## Attaching package: 'IRanges'
```

```
## The following objects are masked from 'package:dplyr':
##
##     collapse, desc, slice
```

```
## Loading required package: GenomeInfoDb
```

```
## Loading required package: GenomicRanges
```

```
## Loading required package: AnnotationDbi
```

```
##
## Attaching package: 'AnnotationDbi'
```

```
## The following object is masked from 'package:MASS':
##
##     select
```

```
## The following object is masked from 'package:dplyr':
##
##     select
```

```
library(readr)
library(tidyr)
```

```
##
## Attaching package: 'tidyr'
```

```
## The following object is masked from 'package:S4Vectors':
##
##     expand
```

```
library(Biobase)
library(goseq)
```

```
## Loading required package: BiasedUrn
```

```
## Loading required package: geneLenDataBase
```

```
##
## Attaching package: 'geneLenDataBase'
```

```
## The following object is masked from 'package:S4Vectors':
##
##     unfactor
```

```
##
```

```
library(org.Hs.eg.db)
```

```
##
```

```
library(org.Rn.eg.db)
```

```
##
```

```
library(phyloseq)
```

```
##
## Attaching package: 'phyloseq'
```

```
## The following object is masked from 'package:GenomicFeatures':
##
##     distance
```

```
## The following object is masked from 'package:GenomicRanges':
##
##     distance
```

```
## The following object is masked from 'package:IRanges':
##
##     distance
```

```
## The following object is masked from 'package:Biobase':
##
##     sampleNames
```

```
library(methylKit)
```

```
##
## Attaching package: 'methylKit'
```

```
## The following object is masked from 'package:tidyr':
##
##     unite
```

```
## The following object is masked from 'package:AnnotationDbi':
##
##     select
```

```
## The following object is masked from 'package:MASS':
##
##     select
```

```
## The following object is masked from 'package:dplyr':
##
##     select
```

```
library(genomation)
```

```
## Loading required package: grid
```

```
##
## Attaching package: 'genomation'
```

```
## The following objects are masked from 'package:methylKit':
##
##     getFeatsWithTargetsStats, getFlanks, getMembers,
##     getTargetAnnotationStats, plotTargetAnnotation
```

```
library(circlize)
```

```
## ========================================
## circlize version 0.4.10
## CRAN page: https://cran.r-project.org/package=circlize
## Github page: https://github.com/jokergoo/circlize
## Documentation: https://jokergoo.github.io/circlize_book/book/
##
## If you use it in published research, please cite:
## Gu, Z. circlize implements and enhances circular visualization
##   in R. Bioinformatics 2014.
##
## This message can be suppressed by:
##   suppressPackageStartupMessages(library(circlize))
## ========================================
```

```
library(ggridges)
library(gridExtra)
```

```
##
## Attaching package: 'gridExtra'
```

```
## The following object is masked from 'package:Biobase':
##
##     combine
```

```
## The following object is masked from 'package:BiocGenerics':
##
##     combine
```

```
## The following object is masked from 'package:dplyr':
##
##     combine
```

```
library(DESeq2)
```

```
## Loading required package: SummarizedExperiment
```

```
## Loading required package: DelayedArray
```

```
## Loading required package: matrixStats
```

```
##
## Attaching package: 'matrixStats'
```

```
## The following objects are masked from 'package:Biobase':
##
##     anyMissing, rowMedians
```

```
## The following object is masked from 'package:dplyr':
##
##     count
```

```
##
## Attaching package: 'DelayedArray'
```

```
## The following objects are masked from 'package:matrixStats':
##
##     colMaxs, colMins, colRanges, rowMaxs, rowMins, rowRanges
```

```
## The following objects are masked from 'package:base':
##
##     aperm, apply, rowsum
```

```
library(kableExtra)
```

```
##
## Attaching package: 'kableExtra'
```

```
## The following object is masked from 'package:dplyr':
##
##     group_rows
```

Water consumption

The daily water consumption per cage was measured before the start of the experiment, and weekly for all the duration of the treatment (13 weeks). Before the final sacrifice and after about 16 hours in metabolic cage, water consumption, was registered for each animal. The means of individual consumptions and related standard deviation were calculated for every group and for sex.

Raw data for water consumption is available as File S2

```
# Load the metadata file containing the rat identification numbers
mixture_metadata  <- read.csv('https://raw.githubusercontent.com/mesnage/MixtureTox/main/mixture_S01.csv')

# Load the raw data for water consumption
raw_water_consumption <- read.csv('https://raw.githubusercontent.com/mesnage/MixtureTox/main/mixture_S02.csv')

# Add a column for the group labels
water <- data.frame(group = factor(c("CONT", "CONT", "CONT", "CONT", "MIX", "MIX", "MIX", "MIX")), raw_water_cons
umption[,-1])

# Calculate averages of water consumption
averages <- vector()
for (i in c(2:15)){
  average <- tapply(water[,i], water$group, FUN=mean)
  average <- as.data.frame(average)
  averages <- append(averages, average)}

# Add a raw for the number of weeks
water_averages <- dplyr::bind_cols(list(averages))
colnames(water_averages) <- colnames(water[,c(2:15)])

# Format for the figure
water_averages <- rbind(water_averages, c(1:14))
water_averages <- as.data.frame(t(water_averages))

# Plot the figure
ggplot() +
  geom_point(data = water_averages, aes(V3, V1), color = "black") +
  geom_smooth(data = water_averages, aes(V3, V1), color = "black",  size = 0.5, method = 'loess', se = TRUE, alph
a = 0.2) + ylim(10,35) +
  geom_point(data = water_averages, aes(V3, V2), color = "firebrick") +
  geom_smooth(data = water_averages, aes(V3, V2, size = 0.1), linetype = 1, color = "firebrick", fill = "firebric
k", size = 0.5, method = 'loess', alpha = 0.2) +
  xlab("Week of treatment") + ylab("Water consumption (ml)") + theme_classic()
```

```
## `geom_smooth()` using formula 'y ~ x'
## `geom_smooth()` using formula 'y ~ x'
```

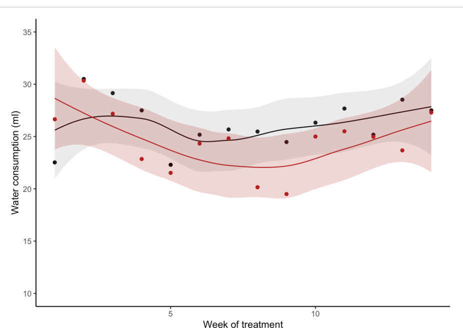

Food consumption

The daily food consumption per cage was measured before the start of the experiment, and weekly for all the duration of the treatment (13 weeks). Before the final sacrifice and after about 16 hours in metabolic cage, water consumption, was registered for each animal. The means of individual consumptions and related standard deviation were calculated for every group and for sex.

Raw data for food consumption is available as File S3.

```
# Load the metadata file containing the rat identification numbers
mixture_metadata  <- read.csv('https://raw.githubusercontent.com/mesnage/MixtureTox/main/mixture_S01.csv')

# Load the raw data for food consumption
raw_feed_consumption <- read.csv('https://raw.githubusercontent.com/mesnage/MixtureTox/main/mixture_S03.csv')

# Add a column for the group labels
feed <- data.frame(group = factor(c("CONT", "CONT", "CONT", "CONT", "MIX", "MIX", "MIX", "MIX")), raw_feed_consum
ption[,-1])

# Calculate averages of food consumption
averages <- vector()
for (i in c(2:15)){
  average <- tapply(feed[,i], feed$group, FUN=mean)
  average <- as.data.frame(average)
  averages <- append(averages, average)}

# Add a raw for the number of weeks
feed_averages <- dplyr::bind_cols(list(averages))
colnames(feed_averages) <- colnames(feed[,c(2:15)])

# Format for the figure
feed_averages <- rbind(feed_averages, c(1:14))
feed_averages <- as.data.frame(t(feed_averages))

# Plot the figure
ggplot() +
  geom_point(data = feed_averages, aes(V3, V1), color = "black") +
  geom_smooth(data = feed_averages, aes(V3, V1), color = "black",  size = 0.5, method = 'loess', se = TRUE, alpha
= 0.2) + ylim(10,30) +
  geom_point(data = feed_averages, aes(V3, V2), color = "firebrick") +
  geom_smooth(data = feed_averages, aes(V3, V2, size = 0.1), linetype = 1, color = "firebrick", fill = "firebrick
", size = 0.5, method = 'loess', alpha = 0.2) +
  xlab("Week of treatment") + ylab("Feed consumption (g)") + theme_classic()
```

```
## `geom_smooth()` using formula 'y ~ x'
## `geom_smooth()` using formula 'y ~ x'
```

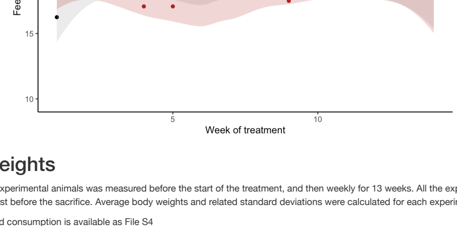

Body weights

Body weight of experimental animals was measured before the start of the treatment, and then weekly for 13 weeks. All the experimental animals were weighted just before the sacrifice. Average body weights and related standard deviations were calculated for each experimental group.

Raw data for food consumption is available as File S4

```
# Load the metadata file containing the rat identification numbers
raw_weights <- read.csv('https://raw.githubusercontent.com/mesnage/MixtureTox/main/mixture_S04.csv')

# Load the raw data for body weights
raw_weights <- data.frame(Group = mixture_metadata$Group, raw_weights[,-1])

# Calculate averages
averages <- vector()
for (i in c(2:15)){
  average <- tapply(raw_weights[,i], raw_weights$Group, FUN=mean)
  average <- as.data.frame(average)
  averages <- append(averages, average)}

# Add a raw for the number of weeks
weights_averages <- dplyr::bind_cols(list(averages))
colnames(weights_averages) <- colnames(raw_weights[,c(2:15)])

# Format for the figure
weights_averages <- rbind(weights_averages, c(1:14))
weights_averages <- as.data.frame(t(weights_averages))

# Plot the figure
ggplot() +
  geom_point(data = weights_averages, aes(V3, V1), color = "black") +
  geom_smooth(data = weights_averages, aes(V3, V1), color = "black",  size = 0.5, method = 'loess', se = TRUE, al
pha = 0.2) + ylim(180,350) +
  geom_point(data = weights_averages, aes(V3, V2), color = "firebrick") +
  geom_smooth(data = weights_averages, aes(V3, V2, size = 0.1), linetype = 1, color = "firebrick", fill = "firebr
ick", size = 0.5, method = 'loess', alpha = 0.2) +
  xlab("Week of treatment") + ylab("Mean weight (g)") + theme_classic()
```

```
## `geom_smooth()` using formula 'y ~ x'
## `geom_smooth()` using formula 'y ~ x'
```

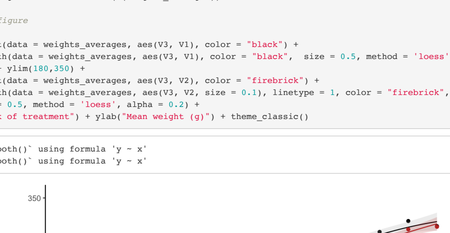

Serum biochemistry

Serum biochemistry was performed under contract by IDEXX BioAnalytics (Stuttgart, Germany), an ISO 17025 accredited laboratory. Briefly, sodium and potassium levels were measured by indirect potentiometry. Albumin was measured by a photometric bromocresol green test. ALP was measured by IFCC with AMP-buffer method, glucose by Enzymatic UV-Test (hexokinase method), cholesterol by Enzymatic color test (CHOD-PAP), blood urea nitrogen by enzymatic UV-Test, gamma-glutamyl-transferase by Kinetic color test International Federation of Clinical Chemistry (IFCC), aspartate and alanine aminotransferase by kinetic UV-test (IFCC+ pyridoxal-5-phosphate), creatinine by kinetic color test (Jaffe's method), lactate dehydrogenase by IFCC method, and triglycerides using an enzymatic color test (GPO-PAP) on a Beckman Coulter AU 480.

Raw data for food consumption is available as File S5

```
# Load the raw data for the serum biochemistry values
biochemistry <- read.csv('https://raw.githubusercontent.com/mesnage/MixtureTox/main/mixture_S05.csv')

# Add the metadata
biochemistry <- dplyr::inner_join(mixture_metadata, biochemistry, by = 'Biochemistry_ID')

# Correct the number format for GGT
biochemistry$GGT <- as.numeric(biochemistry$GGT)

# Make the statistical analysis
pairwise.wilcox.test(biochemistry$Albumin, biochemistry$Group,  paired = FALSE, p.adjust.method = "BH")
```

```
##
##  Pairwise comparisons using Wilcoxon rank sum test with continuity correction
##
## data:  biochemistry$Albumin and biochemistry$Group
##
##     CON
## MIX 0.074
##
## P value adjustment method: BH
```

```
pairwise.wilcox.test(biochemistry$Alkaline.phosphatase, biochemistry$Group,  paired = FALSE, p.adjust.method = "B
H")
```

```
##
##  Pairwise comparisons using Wilcoxon rank sum test with continuity correction
##
## data:  biochemistry$Alkaline.phosphatase and biochemistry$Group
##
##     CON
## MIX 0.088
##
## P value adjustment method: BH
```

```
pairwise.wilcox.test(biochemistry$ALT..GPT., biochemistry$Group,  paired = FALSE, p.adjust.method = "BH")
```

```
##
##  Pairwise comparisons using Wilcoxon rank sum test with continuity correction
##
## data:  biochemistry$ALT..GPT. and biochemistry$Group
##
##     CON
## MIX 0.053
##
## P value adjustment method: BH
```

```
pairwise.wilcox.test(biochemistry$AST..GOT., biochemistry$Group,  paired = FALSE, p.adjust.method = "BH")
```

```
##
##  Pairwise comparisons using Wilcoxon rank sum test with continuity correction
##
## data:  biochemistry$AST..GOT. and biochemistry$Group
##
##     CON
## MIX 0.17
##
## P value adjustment method: BH
```

```
pairwise.wilcox.test(biochemistry$Cholesterol, biochemistry$Group,  paired = FALSE, p.adjust.method = "BH")
```

```
##
##  Pairwise comparisons using Wilcoxon rank sum test with continuity correction
##
## data:  biochemistry$Cholesterol and biochemistry$Group
##
##     CON
## MIX 0.73
##
## P value adjustment method: BH
```

```
pairwise.wilcox.test(biochemistry$Creatinine, biochemistry$Group,  paired = FALSE, p.adjust.method = "BH")
```

```
##
##  Pairwise comparisons using Wilcoxon rank sum test with continuity correction
##
## data:  biochemistry$Creatinine and biochemistry$Group
##
##     CON
## MIX 0.013
##
## P value adjustment method: BH
```

```
pairwise.wilcox.test(biochemistry$GGT, biochemistry$Group,  paired = FALSE, p.adjust.method = "BH")
```

```
##
##  Pairwise comparisons using Wilcoxon rank sum test with continuity correction
##
## data:  biochemistry$GGT and biochemistry$Group
##
##     CON
## MIX 0.4
##
## P value adjustment method: BH
```

```
pairwise.wilcox.test(biochemistry$LDH, biochemistry$Group,  paired = FALSE, p.adjust.method = "BH")
```

```
##
##  Pairwise comparisons using Wilcoxon rank sum test with continuity correction
##
## data:  biochemistry$LDH and biochemistry$Group
##
##     CON
## MIX 0.26
##
## P value adjustment method: BH
```

```
pairwise.wilcox.test(biochemistry$Potassium, biochemistry$Group,  paired = FALSE, p.adjust.method = "BH")
```

```
##
##  Pairwise comparisons using Wilcoxon rank sum test with continuity correction
##
## data:  biochemistry$Potassium and biochemistry$Group
##
##     CON
## MIX 0.099
##
## P value adjustment method: BH
```

```
pairwise.wilcox.test(biochemistry$Sodium, biochemistry$Group,  paired = FALSE, p.adjust.method = "BH")
```

```
##
##  Pairwise comparisons using Wilcoxon rank sum test with continuity correction
##
## data:  biochemistry$Sodium and biochemistry$Group
##
##     CON
## MIX 0.2
##
## P value adjustment method: BH
```

```
pairwise.wilcox.test(biochemistry$Urea..BUN., biochemistry$Group,  paired = FALSE, p.adjust.method = "BH")
```

```
##
##  Pairwise comparisons using Wilcoxon rank sum test with continuity correction
##
## data:  biochemistry$Urea..BUN. and biochemistry$Group
##
##     CON
## MIX 0.099
##
## P value adjustment method: BH
```

```
pairwise.wilcox.test(biochemistry$Triglycerides, biochemistry$Group,  paired = FALSE, p.adjust.method = "BH")
```

```
##
##  Pairwise comparisons using Wilcoxon rank sum test with continuity correction
##
## data:  biochemistry$Triglycerides and biochemistry$Group
##
##     CON
## MIX 1
##
## P value adjustment method: BH
```

```
# Make the plots, one for each marker
ggplot(biochemistry, aes(x=Group, y=Albumin, fill =Group, color=Group)) + geom_boxplot(outlier.shape = NA, alpha
= 0.3) +
  geom_jitter(shape=16, position=position_jitter(0.2)) + ggtitle('p = 0.07') + ylab("Albumin (g/l)") +
  scale_fill_manual(values=c("grey", "firebrick")) + scale_color_manual(values=c("black", "firebrick")) +
  theme_classic() + theme(axis.text.x = element_text(angle = 90), legend.position = "none",axis.title.x = element
_blank())
```

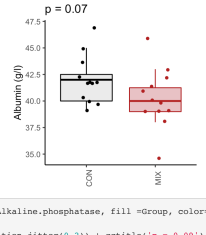


```
ggplot(biochemistry, aes(x=Group, y=Alkaline.phosphatase, fill =Group, color=Group)) + geom_boxplot(outlier.shape
= NA, alpha = 0.3) +
  geom_jitter(shape=16, position=position_jitter(0.2)) + ggtitle('p = 0.09') + ylab("ALP (U/l)") +
  scale_fill_manual(values=c("grey", "firebrick")) + scale_color_manual(values=c("black", "firebrick")) +
  theme_classic() + theme(axis.text.x = element_text(angle = 90), legend.position = "none",axis.title.x = element
_blank())
```

p = 0.09

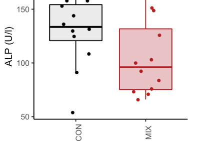


```r
ggplot(biochemistry, aes(x=Group, y=ALT..GPT., fill =Group, color=Group)) + geom_boxplot(outlier.shape = NA, alph
a = 0.3) +
  geom_jitter(shape=16, position=position_jitter(0.2)) + ggtitle('p = 0.05') + ylab('ALT (U/L)') +
  scale_fill_manual(values=c("grey", "firebrick")) + scale_color_manual(values=c("black", "firebrick")) +
  theme_classic() + theme(axis.text.x = element_text(angle = 90), legend.position = "none",axis.title.x = element
_blank())
```

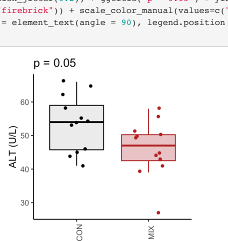


```r
ggplot(biochemistry, aes(x=Group, y=AST..GOT., fill =Group, color=Group)) + geom_boxplot(outlier.shape = NA, alph
a = 0.3) +
  geom_jitter(shape=16, position=position_jitter(0.2)) + ggtitle('p = 0.17') + ylab('AST (U/l)') +
  scale_fill_manual(values=c("grey", "firebrick")) + scale_color_manual(values=c("black", "firebrick")) +
  theme_classic() + theme(axis.text.x = element_text(angle = 90), legend.position = "none",axis.title.x = element
_blank())
```

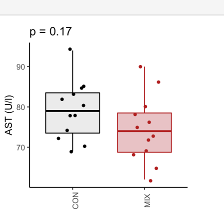


```r
ggplot(biochemistry, aes(x=Group, y=Cholesterol, fill =Group, color=Group)) + geom_boxplot(outlier.shape = NA, al
pha = 0.3) +
  geom_jitter(shape=16, position=position_jitter(0.2)) + ggtitle('p = 0.73') + ylab("Cholesterol (mmol/l)") +
  scale_fill_manual(values=c("grey", "firebrick")) + scale_color_manual(values=c("black", "firebrick")) +
  theme_classic() + theme(axis.text.x = element_text(angle = 90), legend.position = "none",axis.title.x = element
_blank())
```

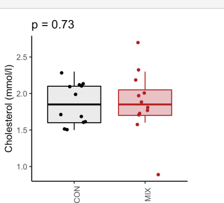


```r
ggplot(biochemistry, aes(x=Group, y=Creatinine, fill =Group, color=Group)) + geom_boxplot(outlier.shape = NA, alp
ha = 0.3) +
  geom_jitter(shape=16, position=position_jitter(0.2)) + ggtitle('p = 0.01') + ylab("Creatinine (µmol/l)")  +
  scale_fill_manual(values=c("grey", "firebrick")) + scale_color_manual(values=c("black", "firebrick")) +
  theme_classic() + theme(axis.text.x = element_text(angle = 90), legend.position = "none",axis.title.x = element
_blank())
```

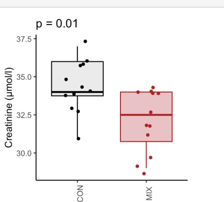


```r
ggplot(biochemistry, aes(x=Group, y=GGT, fill =Group, color=Group)) + geom_boxplot(outlier.shape = NA, alpha = 0.
3) +
  geom_jitter(shape=16, position=position_jitter(0.2)) + ggtitle('p = 0.4') + ylab("GGT (U/l)") +
  scale_fill_manual(values=c("grey", "firebrick")) + scale_color_manual(values=c("black", "firebrick")) +
  theme_classic() + theme(axis.text.x = element_text(angle = 90), legend.position = "none",axis.title.x = element
_blank())
```

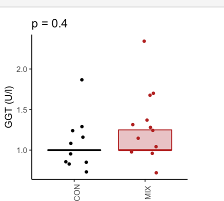


```r
ggplot(biochemistry, aes(x=Group, y=LDH, fill =Group, color=Group))+ geom_boxplot(outlier.shape = NA, alpha = 0.3
) +
  geom_jitter(shape=16, position=position_jitter(0.2)) + ggtitle('p = 0.26') + ylab("LDH (U/l)") +
  scale_fill_manual(values=c("grey", "firebrick")) + scale_color_manual(values=c("black", "firebrick")) +
  theme_classic() + theme(axis.text.x = element_text(angle = 90), legend.position = "none",axis.title.x = element
_blank())
```

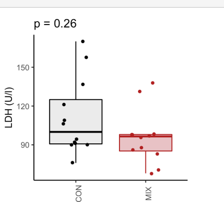


```r
ggplot(biochemistry, aes(x=Group, y=Potassium, fill =Group, color=Group)) + geom_boxplot(outlier.shape = NA, alph
a = 0.3) +
  geom_jitter(shape=16, position=position_jitter(0.2)) + ggtitle('p = 0.09') + ylab("Potassium (mmol/l)") +
  scale_fill_manual(values=c("grey", "firebrick")) + scale_color_manual(values=c("black", "firebrick")) +
  theme_classic() + theme(axis.text.x = element_text(angle = 90), legend.position = "none",axis.title.x = element
_blank())
```

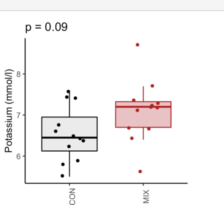


```r
ggplot(biochemistry, aes(x=Group, y=Sodium, fill =Group, color=Group)) + geom_boxplot(outlier.shape = NA, alpha =
0.3) +
  geom_jitter(shape=16, position=position_jitter(0.2)) + ggtitle('p = 0.1') + ylab("Sodium (mmol/l)") +
  scale_fill_manual(values=c("grey", "firebrick")) + scale_color_manual(values=c("black", "firebrick")) +
  theme_classic() + theme(axis.text.x = element_text(angle = 90), legend.position = "none",axis.title.x = element
_blank())
```

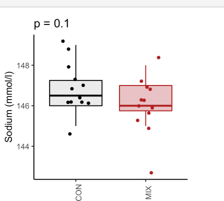


```r
ggplot(biochemistry, aes(x=Group, y=Urea..BUN., fill =Group, color=Group)) + geom_boxplot(outlier.shape = NA, alp
ha = 0.3) +
  geom_jitter(shape=16, position=position_jitter(0.2)) + ggtitle('p = 0.1') + ylab('BUN (mmol/l)') +
  scale_fill_manual(values=c("grey", "firebrick")) + scale_color_manual(values=c("black", "firebrick")) +
  theme_classic() + theme(axis.text.x = element_text(angle = 90), legend.position = "none",axis.title.x = element
_blank())
```

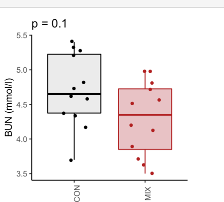


```r
ggplot(biochemistry, aes(x=Group, y=Triglycerides, fill =Group, color=Group)) + geom_boxplot(outlier.shape = NA,
alpha = 0.3) +
  geom_jitter(shape=16, position=position_jitter(0.2)) + ggtitle('p = 1') + ylab("Triglycerides (mmol/l)") +
  scale_fill_manual(values=c("grey", "firebrick")) + scale_color_manual(values=c("black", "firebrick")) +
  theme_classic() + theme(axis.text.x = element_text(angle = 90), legend.position = "none",axis.title.x = element
_blank())
```

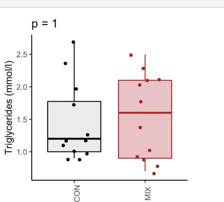

Serum metabolomics

Serum Metabolomics analysis was conducted under contract with Metabolon Inc. (Durham, NC, USA) on four independent instrument platforms as previously described: two different separate reverse phase ultrahigh performance liquid chromatography-tandem mass spectroscopy analysis (RP/UPLC-MS/MS) with positive ion mode electrospray ionization (ESI), a RP/UPLC-MS/MS with negative ion mode ESI, as well as by hydrophilic-interaction chromatography (HILIC)/UPLC-MS/MS with negative ion mode ESI.

The raw data for the peak intensity is available as File S10 The metadata describing annotation, mass and RI is available as File S9 The scaled and imputed data is available as File S11.

The results are available as File S12

Scaled and imputed peak area values were log transformed, and statistical significance determined using a Welch's two-sample t-test adjusted for multiple comparisons with FDR methods using the R package 'qvalue'.

The hypergeometric P-values were computed using the R package Hypergeo

The raw data is available in Metabolights, with the accession number MTBLS138 (https://www.ebi.ac.uk/metabolights/MTBLS138).

We also used orthogonal partial least squares discriminant analysis (OPLS-DA) to evaluate the predictive ability of each omics approach. The R package ropls version 1.20.0 was used. This algorithm uses the nonlinear iterative partial least squares algorithm (NIPALS). Since PLS-DA methods are prone to overfitting, we assessed the significance of our classification using permutation tests (permuted 1,000 times).

```r
# Load the metadata file containing the rat identification numbers
mixture_metadata  <- read.csv('https://raw.githubusercontent.com/mesnage/MixtureTox/main/mixture_S01.csv')

# Load the scaled and imputed dataset, raw values are also available as indicated above
mixture_norm_serum <- read.csv('https://raw.githubusercontent.com/mesnage/MixtureTox/main/mixture_S11.csv', row.n
ames = 1)
mixture_norm_serum <- t(mixture_norm_serum)

# Calculate ratios before log 10 transformation
fold_change <- data.frame(BIOCHEMICAL = colnames(mixture_norm_serum),
                     ratio = abs(colMeans(mixture_norm_serum[11:20,])/colMeans(mixture_norm_serum[1:10,])))
fold_change$fold_change <- ifelse(fold_change$ratio < 1, -1/fold_change$ratio * 2, fold_change$ratio)

# Transformation
mixture_norm_serum <- log10(mixture_norm_serum)

# OPLS-DA
labels <- as.factor(mixture_metadata[-c(11,12,23,24), 2])
serum_mixture.oplsda <- opls(mixture_norm_serum, labels, predI = 1, orthoI = NA, permI = 1000)
```

```
## OPLS-DA
## 20 samples x 748 variables and 1 response
## standard scaling of predictors and response(s)
##       R2X(cum) R2Y(cum) Q2(cum)  RMSEE pre ort  pR2Y   pQ2
## Total     0.36    0.995   0.801 0.0375   1   2 0.001 0.001
```

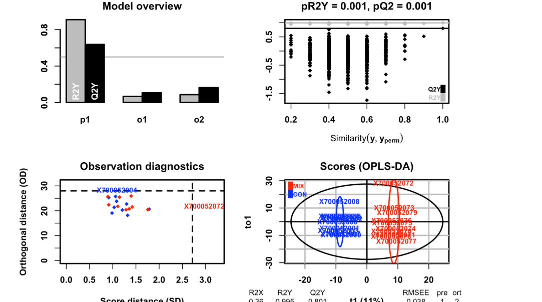


```r
vipVn_serum <- data.frame(BIOCHEMICAL = colnames(mixture_norm_serum), VIP = getVipVn(serum_mixture.oplsda))

# Loop to perform pairwise statistics and populate a table with the p-values
P_value <- vector() ; outcome_list <- vector()
for (i in c(1:ncol(mixture_norm_serum)))
{
  outcome <- colnames(mixture_norm_serum)[i]
  res.aov <- t.test(get(outcome) ~ labels, data = mixture_norm_serum)$p.value
  P_value <- rbind(P_value, res.aov)
  outcome_list <- append(outcome_list, outcome)
}
P_value <- as.numeric(P_value)
anova_table_serum <- data.frame(BIOCHEMICAL = outcome_list, P_value)

# P-value adjustment using the FDR method
anova_table_serum$qvalues <- qvalue::qvalue(anova_table_serum$P_value)$qvalues

# Add the metadata
table_metadata_metabolites_serum <- read.csv('https://raw.githubusercontent.com/mesnage/MixtureTox/main/mixture_S
09.csv')
anova_table_serum <- dplyr::left_join(table_metadata_metabolites_serum, anova_table_serum, by = 'BIOCHEMICAL')

# Add the fold changes
anova_table_serum <- dplyr::left_join(anova_table_serum, fold_change, by = 'BIOCHEMICAL')
anova_table_serum <- dplyr::left_join(anova_table_serum, vipVn_serum, by = 'BIOCHEMICAL')

# Filter the results
Table2_serum <- anova_table_serum[anova_table_serum$VIP > 2,]
Table2_serum <- dplyr::select(Table2_serum[,-1], BIOCHEMICAL, SUB_PATHWAY, fold_change, P_value, qvalues, VIP)

Table2_serum %>%
  kbl(caption = "Table 2. Serum metabolomics of host-gut microbiota interactions in rats exposed to a pesticide m
ixture reveals alterations in multiple metabolic pathways. We presented fold changes (FC) for the metabolites whi
ch were found to have their variable importance in projection (VIP) scores > 2 in the OPLS-DA analyses ") %>%
  kable_classic(c("striped", "hover"), full_width = F, html_font = "Cambria")
```

Table 2. Serum metabolomics of host-gut microbiota interactions in rats exposed to a pesticide mixture reveals alterations in multiple metabolic pathways. We presented fold changes (FC) for the metabolites which were found to have their variable importance in projection (VIP) scores > 2 in the OPLS-DA analyses

	BIOCHEMICAL	SUB_PATHWAY	fold_change	P_value	qvalues	VIP
52	1-methylnicotinamide	Nicotinate and Nicotinamide Metabolism	1.868543	0.0002136	0.0167217	2.293515
171	3-dehydrocholate	Secondary Bile Acid Metabolism	3.873537	0.0010117	0.0371296	2.121620
176	3-hydroxyadipate*	Fatty Acid, Dicarboxylate	-4.170352	0.0002598	0.0167217	2.268392
200	3-methylglutaconate	Leucine, Isoleucine and Valine Metabolism	-4.074065	0.0000112	0.0026713	2.639215
220	4-hydroxyphenylacetate	Phenylalanine Metabolism	-4.075437	0.0000174	0.0027742	2.468102
239	5-methyluridine (ribothymidine)	Pyrimidine Metabolism, Uracil containing	-2.542779	0.0014883	0.0389837	2.050238
268	alpha-ketoglutarate	TCA Cycle	-3.001711	0.0002901	0.0167217	2.238602
366	eicosanedioate (C20-DC)	Fatty Acid, Dicarboxylate	-3.236836	0.0006037	0.0281218	2.126283
411	glutarate (C5-DC)	Fatty Acid, Dicarboxylate	-3.344105	0.0002443	0.0167217	2.291506
440	hexadecenedioate (C16:1-DC)*	Fatty Acid, Dicarboxylate	-4.178458	0.0025273	0.0524245	2.005435
461	indoleacetate	Tryptophan Metabolism	-3.238343	0.0013549	0.0389837	2.088645
500	methionine	Methionine, Cysteine, SAM and Taurine Metabolism	-2.291664	0.0015525	0.0389837	2.003505
507	mevalonate	Mevalonate Metabolism	-2.776503	0.0003154	0.0167217	2.242842
560	N-methyl-GABA	Glutamate Metabolism	-2.477869	0.0006484	0.0281218	2.169258
587	nicotinamide	Nicotinate and Nicotinamide Metabolism	1.459955	0.0012127	0.0385720	2.119168
588	nicotinamide N-oxide	Nicotinate and Nicotinamide Metabolism	3.056255	0.0001882	0.0167217	2.277586
620	perfluorooctanesulfonate (PFOS)	Chemical	-2.492960	0.0009817	0.0371296	2.065826
637	pipecolate	Lysine Metabolism	-6.482148	0.0000004	0.0001671	2.791353
645	pyridoxal	Vitamin B6 Metabolism	-3.191828	0.0013966	0.0389837	2.114407
708	taurine	Methionine, Cysteine, SAM and Taurine Metabolism	1.098289	0.0012017	0.0385720	2.062309
734	tryptophan	Tryptophan Metabolism	-2.268885	0.0016970	0.0404817	2.020466

```r
# Enrichment analysis
# m = total number of metabolites measured for the given pathway
pathways <- as.data.frame(table(anova_table_serum$SUB_PATHWAY))
colnames(pathways)[2] <- 'm'

# x = number of metabolites disturbed for the given pathway
significant <- subset(anova_table_serum, VIP > 2)
significant <- as.data.frame(table(significant$SUB_PATHWAY))
enrichement <- dplyr::left_join(pathways, significant, by = 'Var1')
colnames(enrichement)[3] <- 'x'

# k = total number of metabolites having their levels altered for all pathways
enrichement$k <- sum(significant$Freq)

# n = Number of "non-marked" elements, i.e. metabolites not associated to this pathway
enrichement$n <- length(anova_table_serum$SUB_PATHWAY) - enrichement$m

# N = total number of metabolites measured
enrichement$N <- length(anova_table_serum$SUB_PATHWAY)

# exp.x = number of significant metabolites expected by chance
enrichement <-  dplyr::mutate(enrichement, exp.x =  k * (m/N))

# fold.enrichment = (x / k ) / (m / N)
enrichement <-  dplyr::mutate(enrichement, fold.enrichment = (x / k ) / (m/N))

# https://www.pathwaycommons.org/guide/primers/statistics/fishers_exact_test/
# http://pedagogix-tagc.univ-mrs.fr/courses/ASG1/practicals/go_statistics_td/go_statistics_td_2015.html

# Computing the hypergeometric P-value
# The P-value is the probability to observe at least "x" marked balls in the selection.
# Compute the P-value for the observed number of marked elements in the selection
enrichement <- dplyr::mutate(enrichement, p.value = phyper(q=x -1, m=m, n=n, k=k, lower.tail=FALSE))

enrichement <- enrichement[enrichement$p.value < 0.05,]
enrichement <- enrichement[!is.na(enrichement$Var1),]


enrichement %>%
  kbl(caption = "Pathway enrichement in serum metanbolome") %>%
  kable_classic(c("striped", "hover"), full_width = F, html_font = "Cambria")
```

Pathway enrichement in serum metanbolome

	Var1	m	x	k	n	N	exp.x	fold.enrichment	p.value
36	Fatty Acid, Dicarboxylate	27	4	21	721	748	0.7580214	5.276896	0.0053032
64	Nicotinate and Nicotinamide Metabolism	7	3	21	741	748	0.1965241	15.265306	0.0006228

Caecum metabolomics

Caecum Metabolomics analysis was conducted under contract with Metabolon Inc. (Durham, NC, USA) on four independent instrument platforms as previously described: two different separate reverse phase ultrahigh performance liquid chromatography-tandem mass spectroscopy analysis (RP/UPLC-MS/MS) with positive ion mode electrospray ionization (ESI), a RP/UPLC-MS/MS with negative ion mode ESI, as well as by hydrophilic-interaction chromatography (HILIC)/UPLC-MS/MS with negative ion mode ESI.

The raw data for the peak intensity is available as File S7 The metadata describing annotation, mass and RI is available as File S6 The scaled and imputed data is available as File S8.

Results are available as file S13

Scaled and imputed peak area values were log transformed, and statistical significance determined using a Welch's two-sample t-test adjusted for multiple comparisons with FDR methods using the R package 'qvalue'.

The hypergeometric P-values were computed using the R package Hypergeo.

The raw data is available in Metabolights, with the accession number MTBLS138 (https://www.ebi.ac.uk/metabolights/MTBLS138).

We also used orthogonal partial least squares discriminant analysis (OPLS-DA) to evaluate the predictive ability of each omics approach. The R package ropls version 1.20.0 was used. This algorithm uses the nonlinear iterative partial least squares algorithm (NIPALS). Since PLS-DA methods are prone to overfitting, we assessed the significance of our classification using permutation tests (permuted 1,000 times).

```r
# Load the metadata file containing the rat identification numbers
mixture_metadata  <- read.csv('https://raw.githubusercontent.com/mesnage/MixtureTox/main/mixture_S01.csv')

# Load the scaled and imputed dataset, raw values are also available as indicated above
mixture_norm_caecum <- read.csv('https://raw.githubusercontent.com/mesnage/MixtureTox/main/mixture_S08.csv', row.
names = 1)
mixture_norm_caecum <- t(mixture_norm_caecum)

# Calculate ratios before log 10 transformation
fold_change <- data.frame(BIOCHEMICAL = colnames(mixture_norm_caecum),
                     ratio = abs(colMeans(mixture_norm_caecum[11:20,])/colMeans(mixture_norm_caecum[1:10,])))
fold_change$fold_change <- ifelse(fold_change$ratio < 1, -1/fold_change$ratio * 2, fold_change$ratio)

# Transformation
mixture_norm_caecum <- log10(mixture_norm_caecum)

# OPLS-DA
labels <- as.factor(mixture_metadata[-c(11,12,23,24), 2])
caecum_mixture.oplsda <- opls(mixture_norm_caecum, labels, predI = 1, orthoI = NA, permI = 1000)
```

```
## OPLS-DA
## 20 samples x 744 variables and 1 response
## standard scaling of predictors and response(s)
##       R2X(cum) R2Y(cum) Q2(cum) RMSEE pre ort  pR2Y   pQ2
## Total    0.361    0.862   0.447 0.201   1   1 0.138 0.012
```

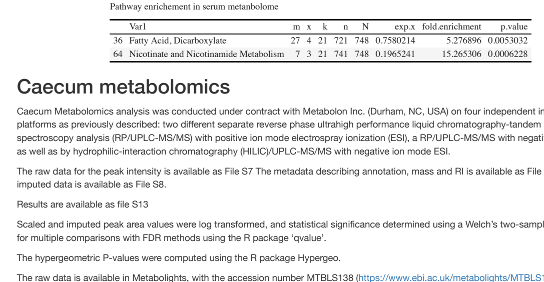


```r
vipVn_caecum <- data.frame(BIOCHEMICAL = colnames(mixture_norm_caecum), VIP = getVipVn(caecum_mixture.oplsda))

# Loop to perform pairwise statistics and populate a table with the p-values
P_value <- vector() ; outcome_list <- vector()
for (i in c(1:ncol(mixture_norm_caecum)))
{
  outcome <- colnames(mixture_norm_caecum)[i]
  res.aov <- t.test(get(outcome) ~ labels, data = mixture_norm_caecum)$p.value
  P_value <- rbind(P_value, res.aov)
  outcome_list <- append(outcome_list, outcome)
}
P_value <- as.numeric(P_value)
anova_table_caecum <- data.frame(BIOCHEMICAL = outcome_list, P_value)

# P-value adjustment using the FDR method
anova_table_caecum$qvalues <- qvalue::qvalue(anova_table_caecum$P_value)$qvalues

# Add the metadata
table_metadata_metabolites_caecum <- read.csv('https://raw.githubusercontent.com/mesnage/MixtureTox/main/mixture_
S06.csv')
anova_table_caecum <- dplyr::left_join(table_metadata_metabolites_caecum, anova_table_caecum, by = 'BIOCHEMICAL')

# Add the fold changes
anova_table_caecum <- dplyr::left_join(anova_table_caecum, fold_change, by = 'BIOCHEMICAL')
anova_table_caecum <- dplyr::left_join(anova_table_caecum, vipVn_caecum, by = 'BIOCHEMICAL')

# Filter the results
Table2_caecum <- anova_table_caecum[anova_table_caecum$VIP > 2,]
Table2_caecum <- dplyr::select(Table2_caecum[,-1], BIOCHEMICAL, SUB_PATHWAY, fold_change, P_value, qvalues, VIP)

Table2_caecum %>%
  kbl(caption = "Table 2. Caecum metabolomics of host-gut microbiota interactions in rats exposed to a pesticide
mixture reveals alterations in multiple metabolic pathways. We presented fold changes (FC) for the metabolites wh
ich were found to have their variable importance in projection (VIP) scores > 2 in the OPLS-DA analyses ") %>%
  kable_classic(c("striped", "hover"), full_width = F, html_font = "Cambria")
```

Table 2. Caecum metabolomics of host-gut microbiota interactions in rats exposed to a pesticide mixture reveals alterations in multiple metabolic pathways. We presented fold changes (FC) for the metabolites which were found to have their variable importance in projection (VIP) scores > 2 in the OPLS-DA analyses

	BIOCHEMICAL	SUB_PATHWAY	fold_change	P_value	qvalues	VIP
20	1,2-dioleoyl-GPE (18:1/18:1)	Phosphatidylethanolamine (PE)	2.820274	0.0005026	0.2470719	2.284360
26	1-(1-enyl-palmitoyl)-2-arachidonoyl-GPE (P-16:0/20:4)*	Plasmalogen	2.518222	0.0354419	0.5469095	2.163798
29	1-(1-enyl-stearoyl)-2-arachidonoyl-GPE (P-18:0/20:4)*	Plasmalogen	3.544276	0.0148949	0.4689886	2.395544
62	1-palmitoyl-2-oleoyl-GPC (16:0/18:1)	Phosphatidylcholine (PC)	2.294500	0.0308946	0.5401360	2.263955
63	1-palmitoyl-2-oleoyl-GPE (16:0/18:1)	Phosphatidylethanolamine (PE)	2.184670	0.0013659	0.2470719	2.412127
64	1-palmitoyl-2-oleoyl-GPG (16:0/18:1)	Phosphatidylglycerol (PG)	2.157077	0.0013174	0.2470719	2.429532
66	1-palmitoyl-2-palmitoleoyl-GPC (16:0/16:1)*	Phosphatidylcholine (PC)	2.210035	0.0383205	0.5469095	2.150178
78	1-stearoyl-2-arachidonoyl-GPE (18:0/20:4)	Phosphatidylethanolamine (PE)	2.582135	0.0307961	0.5401360	2.059660
82	1-stearoyl-2-oleoyl-GPC (18:0/18:1)	Phosphatidylcholine (PC)	2.467556	0.0499279	0.6225844	2.125135
83	1-stearoyl-2-oleoyl-GPE (18:0/18:1)	Phosphatidylethanolamine (PE)	1.767620	0.0526008	0.6331753	2.018441
301	citrulline	Urea cycle; Arginine and Proline Metabolism	1.622133	0.0017691	0.2470719	2.464893
386	glutamate	Glutamate Metabolism	1.275797	0.0722507	0.6477801	2.026861
395	glycerophosphoglycerol	Glycerolipid Metabolism	-3.653287	0.0415881	0.5479441	2.118474
417	heptadecanedioate (C17-DC)	Fatty Acid, Dicarboxylate	1.344488	0.0874674	0.6629660	2.069220
419	heptanoate (7:0)	Medium Chain Fatty Acid	1.561109	0.0081178	0.4689886	2.058543
421	hexadecanedioate (C16-DC)	Fatty Acid, Dicarboxylate	1.897052	0.0027731	0.2766321	2.435610
517	N-acetylarginine	Urea cycle; Arginine and Proline Metabolism	1.298041	0.0237419	0.5013286	2.158447
547	N-carbamoylaspartate	Pyrimidine Metabolism, Orotate containing	1.711664	0.0205077	0.4938133	2.098495
590	nicotinamide riboside	Nicotinate and Nicotinamide Metabolism	1.368962	0.0139993	0.4689886	2.346398
611	palmitoyl dihydrosphingomyelin (d18:0/16:0)*	Dihydrosphingomyelins	2.702084	0.0224838	0.5013286	2.057530
613	palmitoyl sphingomyelin (d18:1/16:0)	Sphingomyelins	2.154016	0.0163066	0.4689886	2.153065
618	pantothenate	Pantothenate and CoA Metabolism	1.391333	0.0173696	0.4689886	2.311754
647	pyridoxal	Vitamin B6 Metabolism	1.303546	0.0067361	0.4669494	2.238478
669	serotonin	Tryptophan Metabolism	1.614914	0.0026051	0.2766321	2.504380
683	stearoyl sphingomyelin (d18:1/18:0)	Sphingomyelins	3.874657	0.0073556	0.4669494	2.459658

```r
# Enrichment analysis
# m = total number of metabolites measured for the given pathway
pathways <- as.data.frame(table(anova_table_caecum$SUB_PATHWAY))
colnames(pathways)[2] <- 'm'

# x = number of metabolites disturbed for the given pathway
significant <- subset(anova_table_caecum, VIP > 2)
significant <- as.data.frame(table(significant$SUB_PATHWAY))
enrichement <- dplyr::left_join(pathways, significant, by = 'Var1')
colnames(enrichement)[3] <- 'x'

# k = total number of metabolites having their levels altered for all pathways
enrichement$k <- sum(significant$Freq)

# n = Number of "non-marked" elements, i.e. metabolites not associated to this pathway
enrichement$n <- length(anova_table_caecum$SUB_PATHWAY) - enrichement$m

# N = total number of metabolites measured
enrichement$N <- length(anova_table_caecum$SUB_PATHWAY)

# exp.x = number of significant metabolites expected by chance
enrichement <-  dplyr::mutate(enrichement, exp.x =  k * (m/N))

# fold.enrichment = (x / k ) / (m / N)
enrichement <-  dplyr::mutate(enrichement, fold.enrichment = (x / k ) / (m/N))

# https://www.pathwaycommons.org/guide/primers/statistics/fishers_exact_test/
# http://pedagogix-tagc.univ-mrs.fr/courses/ASG1/practicals/go_statistics_td/go_statistics_td_2015.html

# Computing the hypergeometric P-value
# The P-value is the probability to observe at least "x" marked balls in the selection.
# Compute the P-value for the observed number of marked elements in the selection
enrichement <- dplyr::mutate(enrichement, p.value = phyper(q=x -1, m=m, n=n, k=k, lower.tail=FALSE))

enrichement <- enrichement[enrichement$p.value < 0.05,]
enrichement <- enrichement[!is.na(enrichement$Var1),]


enrichement %>%
  kbl(caption = "Pathway enrichement in caecum metabolome") %>%
  kable_classic(c("striped", "hover"), full_width = F, html_font = "Cambria")
```

Pathway enrichement in caecum metabolome

	Var1	m	x	k	n	N	exp.x	fold.enrichment	p.value
14	Dihydrosphingomyelins	1	1	25	743	744	0.0336022	29.760000	0.0336022
70	Phosphatidylcholine (PC)	17	3	25	727	744	0.5712366	5.251765	0.0167092
71	Phosphatidylethanolamine (PE)	10	4	25	734	744	0.3360215	11.904000	0.0001828
72	Phosphatidylglycerol (PG)	1	1	25	743	744	0.0336022	29.760000	0.0336022
75	Plasmalogen	5	2	25	739	744	0.1680108	11.904000	0.0101960
91	Sphingomyelins	2	2	25	742	744	0.0672043	29.760000	0.0010854

Caecum shotgun metagenomics

Shotgun metagenomics was performed by GenomeScan (Leiden, The Netherlands). The NEBNext® Ultra II FS DNA module (cat# NEB #E7810S/L) and the NEBNext® Ultra II Ligation module (cat# NEB #E7595S/L) were used to process the samples.

The shotgun metagenomics data was pre-processed using the pre-processing package v0.2.2 (https://anaconda.org/fasnicar/preprocessing). In brief, this package concatenates reads, to remove Illumina adapters, discard low-quality (quality <20 or >2 Ns) or too short reads (< 75bp), remove phiX and rat genome sequences, and finally sorts and splits the reads into R1, R2, and UN sets of reads.

The raw data are available from the National Center for Biotechnology Information (NCBI), with BioProject accession no. PRJNA609596 (https://www.ncbi.nlm. nih.gov/bioproject/PRJNA609596).

Cleaned shotgun metagenomics reads were then processed for taxonomic and pathway profiling. Since there is no gold standard for computational analyses of shotgun metagenomics, we used a combination of approaches. We inferred the taxonomy with the RefSeq database on the metagenomics RAST server, IGGsearch (iggdb_v1.0.0_gut database), MetaPhlAn version 3.0 and Kaiju 1.0.1.

IGG species count data table: File S14 Phylum RefSeq: File S16 Species RefSeq: File S17 Kaiju species table: File S19 Metaphlan3 species table: File S21 KO level 3 function table: File S23 KO pathway table: File S25

We used ALDEx version 2 (ALDEx2) for differential (relative) abundance analysis of proportional data 56. Statistical analysis for taxa abundance was performed on a dataset corrected for asymmetry (uneven sequencing depths) using the inter-quartile log-ratio method, which identifies features with reproducible variance.

IGG statistical analysis with Adlex2: File S15 Species RefSeq statistical analysis with Adlex2: File S18 Kaiju statistical analysis with Adlex2: File S20 KO level 3 statistical analysis with Adlex2: File S24 KO pathway statistical analysis with Adlex2: File S22

Metaphlan3 statistical analysis was done using a kruskall-wallis test because the data was not counts but relative abundances values.

A multivariate analysis consisting in a non-metric multidimensional scaling (NMDS) plot of Bray-Curtis distances between samples. Statistical significance of the sample clustering was evaluated with a permutational ANOVA (PERMANOVA) analysis on the Bray-Curtis distances with adonis() from vegan v2.4-2.

```r
# Load the phylum profiles determining according to the RefSeq database
plotdata <- read.csv('https://raw.githubusercontent.com/mesnage/MixtureTox/main/mixture_S16.csv')

# Load the metadata file containing the rat identification numbers
mixture_metadata  <- read.csv('https://raw.githubusercontent.com/mesnage/MixtureTox/main/mixture_S01.csv')

# Calculate the total abundance
plotdata[,c(2:ncol(plotdata))] = apply(plotdata[,c(2:ncol(plotdata))], 2, function(x){x/sum(x)*100})
plotdata <- t(data.frame(plotdata[,-1], row.names = plotdata$phylum))
plotdata <- data.frame(condition = mixture_metadata$Group, plotdata)

# Calculate the total abundance for the rare taxa
plotdata$others <- rowSums(plotdata[,c(10:ncol(plotdata))])
plotdata <- plotdata[,-c(10:63)]

# Add a column for the rat identification numbers
plotdata$RID <- row.names(plotdata)

# Format the table in a long format for the use in ggplot2
plotdata <- reshape2:::melt(plotdata, id = c("RID", "condition"))
colnames(plotdata) <- c("sample", "var", "variable", "value")

# Plot the figure
ggplot(data=plotdata, aes(x=sample, y=value, colour=variable, fill = variable)) +
  geom_bar(stat="identity", position="stack") + facet_wrap(~var, scales = "free", ncol = 2) +
  scale_colour_brewer(palette = "Set1")  +
  scale_fill_brewer(palette = "Set1") + theme_classic() +
  ylab("total abundance (%)") +
  theme(axis.text.x=element_blank(), axis.title.x=element_blank(), legend.position = "right",
        axis.ticks.x=element_blank())
```

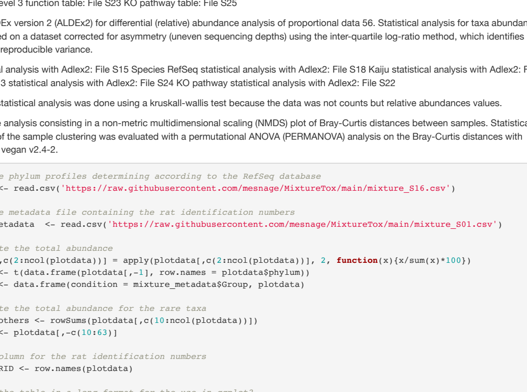


```r
# Load the metadata file containing the rat identification numbers
mixture_metadata  <- read.csv('https://raw.githubusercontent.com/mesnage/MixtureTox/main/mixture_S01.csv')

# Load the taxa profiles determined with IGGsearch
raw_metagenome_igg <- read.csv('https://raw.githubusercontent.com/mesnage/MixtureTox/main/mixture_S14.csv')

# Format the tables to fit Phyloseq objects requirements
row.names(raw_metagenome_igg) <- raw_metagenome_igg$X
taxa <- data.frame(taxa = row.names(raw_metagenome_igg), row.names = row.names(raw_metagenome_igg))
raw_metagenome_igg <- raw_metagenome_igg[,-1]
TAX = tax_table(taxa)
row.names(raw_metagenome_igg) <- taxa_names(TAX)
OTU = otu_table(as.matrix(raw_metagenome_igg), taxa_are_rows = TRUE)
samples = sample_data(data.frame(condition = mixture_metadata$Group, id = colnames(raw_metagenome_igg), row.names
= colnames(raw_metagenome_igg)))
physeq <- phyloseq(OTU, TAX, samples)

# Calculate the total abundance
physeq_metabotype_ra = transform_sample_counts(physeq, function(x){x / sum(x)})
bray_dist = phyloseq::distance(physeq_metabotype_ra, method="bray", weighted=F)
vegan::adonis(bray_dist ~ sample_data(physeq_metabotype_ra)$condition, permutations = 10000)
```

```
##
## Call:
## vegan::adonis(formula = bray_dist ~ sample_data(physeq_metabotype_ra)$condition,      permutations = 10000)
##
## Permutation: free
## Number of permutations: 10000
##
## Terms added sequentially (first to last)
##
##                                             Df SumsOfSqs MeanSqs F.Model
## sample_data(physeq_metabotype_ra)$condition  1    0.1782 0.17824  1.2807
## Residuals                                   22    3.0620 0.13918
## Total                                       23    3.2403
##                                                  R2 Pr(>F)
## sample_data(physeq_metabotype_ra)$condition 0.05501 0.2072
## Residuals                                   0.94499
## Total                                       1.00000
```

```r
# Transform samples in log
physeq_metabotype_log <- transform_sample_counts(physeq, function(x) log(1 + x))
physeq.ord <- ordinate(physeq_metabotype_log, "NMDS", "bray")
```

```
## Wisconsin double standardization
## Run 0 stress 0.1584666
## Run 1 stress 0.158466
## ... New best solution
## ... Procrustes: rmse 0.0002648669  max resid 0.0009427755
## ... Similar to previous best
## Run 2 stress 0.1584674
## ... Procrustes: rmse 0.001421653  max resid 0.002748633
## ... Similar to previous best
## Run 3 stress 0.1372819
## ... New best solution
## ... Procrustes: rmse 0.1355382  max resid 0.4640767
## Run 4 stress 0.1599374
## Run 5 stress 0.1372825
## [illegible]
```

```
## ... Procrustes: rmse 0.0003085198  max resid 0.001093138
## ... Similar to previous best
## Run 6 stress 0.1753461
## Run 7 stress 0.159625
## Run 8 stress 0.159625
## Run 9 stress 0.137282
## ... Procrustes: rmse 0.000109892  max resid 0.0004311715
## ... Similar to previous best
## Run 10 stress 0.1372836
## ... Procrustes: rmse 0.0008501085  max resid 0.002807379
## ... Similar to previous best
## Run 11 stress 0.1856262
## Run 12 stress 0.1584691
## Run 13 stress 0.137283
## ... Procrustes: rmse 0.0007894959  max resid 0.002595491
## ... Similar to previous best
## Run 14 stress 0.1860145
## Run 15 stress 0.159625
## Run 16 stress 0.137282
## ... Procrustes: rmse 0.000117563  max resid 0.0004617672
## ... Similar to previous best
## Run 17 stress 0.1372827
## ... Procrustes: rmse 0.0003565052  max resid 0.001249671
## ... Similar to previous best
## Run 18 stress 0.172211
## Run 19 stress 0.1372825
## ... Procrustes: rmse 0.0003448359  max resid 0.001347966
## ... Similar to previous best
## Run 20 stress 0.1733707
## *** Solution reached
```

```r
# Plot beta diversity by extracting the sample coordinates and plotting them with ggplot2
plot_beta_diversity <- plot_ordination(physeq_metabotype_log, physeq.ord, color="condition")
plot_beta_diversity$layers <- plot_beta_diversity$layers[-c(1,2)]
plot_beta_diversity +
  theme(aspect.ratio=1) + theme_bw() +  geom_point(size = 1.5, alpha = 0.8, position = "jitter") +
  scale_colour_manual(values = c("black", "firebrick")) + scale_fill_manual(values = c("black", "firebrick"))
```

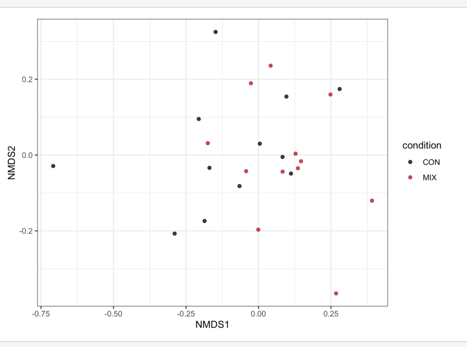


```r
# Load the metadata file containing the rat identification numbers
mixture_metadata  <- read.csv('https://raw.githubusercontent.com/mesnage/MixtureTox/main/mixture_S01.csv')

# Load the taxa profiles determined with IGGsearch as a table with OTU in columns and samples as rows
raw_metagenome_igg <- read.csv('https://raw.githubusercontent.com/mesnage/MixtureTox/main/mixture_S14.csv')
raw_metagenome_igg <- data.frame(raw_metagenome_igg[,-1], row.names = raw_metagenome_igg$X)
raw_metagenome_igg <- as.data.frame(t(raw_metagenome_igg))

# Filter rare taxa
raw_metagenome_igg[raw_metagenome_igg == 0] <- NA
raw_metagenome_igg <- raw_metagenome_igg %>% dplyr::select(which(colMeans(is.na(.)) < 0.2))
raw_metagenome_igg[is.na(raw_metagenome_igg)] <- 0
raw_metagenome_igg <- t(raw_metagenome_igg)

# Perform Aldex search
aldex_iggsearch <- ALDEx2::aldex(raw_metagenome_igg, mixture_metadata$Group, mc.samples=128, test="t", effect=TRU
E, include.sample.summary=FALSE, denom="iqlr", verbose=FALSE)
```

```
## aldex.clr: generating Monte-Carlo instances and clr values
```

```
## operating in serial mode
```

```
## computing iqlr centering
```

```
## aldex.ttest: doing t-test
```

```
## aldex.effect: calculating effect sizes
```

```r
aldex_iggsearch <- aldex_iggsearch[order(aldex_iggsearch$wi.eBH),]
aldex_iggsearch <- round(aldex_iggsearch, digits = 2)

head(aldex_iggsearch, 10)  %>%
  kbl(caption = "IGGsearch OTUs which are the most different between the two groups") %>%
  kable_classic(c("striped", "hover"), full_width = F, html_font = "Cambria")
```

IGGsearch OTUs which are the most different between the two groups

	rab.all	rab.win.CON	rab.win.MIX	diff.btw	diff.win	effect	overlap	we.ep	we.eBH	wi.ep	wi.eBH
OTU-05234	4.02	4.90	3.28	-1.79	2.51	-0.76	0.16	0.01	0.46	0.00	0.31
OTU-16780	-0.84	0.78	-1.48	-2.51	2.73	-0.80	0.17	0.07	0.55	0.01	0.31
OTU-05508	-1.63	-2.17	-0.43	2.02	2.70	0.73	0.21	0.01	0.46	0.02	0.37
OTU-16795	-1.55	-0.07	-2.82	-2.77	3.53	-0.61	0.21	0.12	0.63	0.02	0.40
OTU-04055	4.21	2.90	4.89	1.52	2.13	0.69	0.22	0.01	0.48	0.02	0.42
OTU-13483	-0.41	-0.84	0.48	1.17	1.62	0.64	0.23	0.05	0.55	0.03	0.43
OTU-05180	3.15	2.76	3.69	1.61	3.10	0.49	0.24	0.03	0.52	0.03	0.45
OTU-04057	5.59	4.55	6.15	1.38	1.86	0.67	0.25	0.02	0.50	0.03	0.45
OTU-05246	6.35	6.76	5.70	-1.12	1.76	-0.60	0.24	0.04	0.53	0.04	0.46
OTU-12008	-1.79	0.18	-2.26	-2.15	3.02	-0.56	0.25	0.16	0.70	0.05	0.48

```r
# Load the taxa profiles determined with RefSeq species  as a table with OTU in columns and samples as rows
raw_metagenome_refseq_species <- read.csv('https://raw.githubusercontent.com/mesnage/MixtureTox/main/mixture_S17.
csv')
raw_metagenome_refseq_species <- data.frame(raw_metagenome_refseq_species[,-1], row.names = raw_metagenome_refseq
_species$species)
raw_metagenome_refseq_species <- as.data.frame(t(raw_metagenome_refseq_species))

# Filter rare taxa
raw_metagenome_refseq_species[raw_metagenome_refseq_species == 0] <- NA
raw_metagenome_refseq_species <- raw_metagenome_refseq_species %>% dplyr::select(which(colMeans(is.na(.)) < 0.2))
raw_metagenome_refseq_species[is.na(raw_metagenome_refseq_species)] <- 0
raw_metagenome_refseq_species <- t(raw_metagenome_refseq_species)

# Perform Aldex search
aldex_refseq_species <- ALDEx2::aldex(raw_metagenome_refseq_species, mixture_metadata$Group, mc.samples=128, test
="t", effect=TRUE, include.sample.summary=FALSE, denom="iqlr", verbose=FALSE)
```

```
## aldex.clr: generating Monte-Carlo instances and clr values
```

```
## operating in serial mode
```

```
## computing iqlr centering
```

```
## aldex.ttest: doing t-test
```

```
## aldex.effect: calculating effect sizes
```

```r
aldex_refseq_species <- aldex_refseq_species[order(aldex_refseq_species$wi.eBH),]
aldex_refseq_species <- round(aldex_refseq_species, digits = 2)

head(aldex_refseq_species, 10) %>%
  kbl(caption = "RefSeq species which are the most different between the two groups") %>%
  kable_classic(c("striped", "hover"), full_width = F, html_font = "Cambria")
```

RefSeq species which are the most different between the two groups

	rab.all	rab.win.CON	rab.win.MIX	diff.btw	diff.win	effect	overlap	we.ep	we.eBH	wi.ep	wi.eBH
Bifidobacterium catenulatum	-2.29	-2.03	-2.49	-0.45	0.49	-0.88	0.11	0.01	0.49	0	0.23
Acidovorax citrulli	-1.11	-1.21	-1.00	0.23	0.23	0.74	0.14	0.15	0.69	0	0.26
Parascardovia denticolens	-1.85	-1.73	-1.96	-0.25	0.26	-0.97	0.15	0.00	0.49	0	0.26
Epulopiscium sp. 'N.t. morphotype B'	0.20	0.32	0.11	-0.25	0.25	-0.82	0.15	0.03	0.50	0	0.26
Methylococcus capsulatus	-0.35	-0.48	-0.25	0.26	0.25	0.78	0.14	0.08	0.56	0	0.26
Ralstonia solanacearum	-0.93	-1.08	-0.76	0.35	0.38	0.83	0.16	0.01	0.49	0	0.27
Bifidobacterium longum	2.91	3.28	2.67	-0.53	0.64	-0.81	0.15	0.00	0.49	0	0.27
Rattus norvegicus	-2.88	-2.19	-3.36	-1.05	1.36	-0.68	0.16	0.01	0.49	0	0.27
Pantoea sp. At-9b	-1.51	-1.77	-1.31	0.42	0.46	0.87	0.16	0.01	0.49	0	0.27
Sebaldella termitidis	1.65	1.73	1.59	-0.14	0.14	-0.76	0.16	0.37	0.84	0	0.27

```r
# Load the taxa profiles determined with Kaiju as a table with OTU in columns and samples as rows
raw_metagenome_kaiju <- read.csv('https://raw.githubusercontent.com/mesnage/MixtureTox/main/mixture_S19.csv')
raw_metagenome_kaiju <- data.frame(raw_metagenome_kaiju[,-1], row.names = raw_metagenome_kaiju$unique)
raw_metagenome_kaiju <- as.data.frame(t(raw_metagenome_kaiju))

# Filter rare taxa
raw_metagenome_kaiju[raw_metagenome_kaiju == 0] <- NA
raw_metagenome_kaiju <- raw_metagenome_kaiju %>% dplyr::select(which(colMeans(is.na(.)) < 0.2))
raw_metagenome_kaiju[is.na(raw_metagenome_kaiju)] <- 0
raw_metagenome_kaiju <- t(raw_metagenome_kaiju)

# Perform Aldex search
aldex_kaiju <- ALDEx2::aldex(raw_metagenome_kaiju, mixture_metadata$Group, mc.samples=128, test="t", effect=TRUE,
include.sample.summary=FALSE, denom="iqlr", verbose=FALSE)
```

```
## aldex.clr: generating Monte-Carlo instances and clr values
```

```
## operating in serial mode
```

```
## computing iqlr centering
```

```
## aldex.ttest: doing t-test
```

```
## aldex.effect: calculating effect sizes
```

```r
aldex_kaiju <- aldex_kaiju[order(aldex_kaiju$wi.eBH),]
aldex_kaiju <- round(aldex_kaiju, digits = 2)

head(aldex_kaiju, 10) %>%
  kbl(caption = "Kaiju taxa which are the most different between the two groups") %>%
  kable_classic(c("striped", "hover"), full_width = F, html_font = "Cambria")
```

Kaiju taxa which are the most different between the two groups

	rab.all	rab.win.CON	rab.win.MIX	diff.btw	diff.win	effect	overlap	we.ep	we.eBH	wi.ep	wi.eBH
cellular organisms_Eukaryota_Fornicata_Diplomonadida_Hexamitidae_Giardiinae_Giardia_Giardia intestinalis_Giardia lamblia P15_NA_NA_NA_NA_NA_NA_NA_NA_NA_NA_NA_NA	-1.94	3.03	-3.45	-6.38	3.16	-1.59	0.04	0	0.15	0	0.13
cellular organisms_Bacteria_Terrabacteria group_Firmicutes_Bacilli_Bacillales_Planococcaceae_Paenisporosarcina_Paenisporosarcina quisquiliarum_NA_NA_NA_NA_NA_NA_NA_NA_NA_NA_NA_NA	-0.01	0.70	-0.63	-1.44	1.03	-1.30	0.06	0	0.31	0	0.14
cellular organisms_Bacteria_Proteobacteria_Gammaproteobacteria_unclassified Gammaproteobacteria_Competibacteraceae_Candidatus Competibacter_Candidatus Competibacter denitrificans_Candidatus Competibacter denitrificans Run_A_D11_NA_NA_NA_NA_NA_NA_NA_NA_NA_NA_NA_NA	0.75	0.39	1.10	0.79	0.62	1.23	0.07	0	0.29	0	0.15
cellular organisms_Bacteria_Terrabacteria group_Actinobacteria_Actinobacteria_Micrococcales_Microbacteriaceae_Leucobacter_Leucobacter sp. 7(1)_NA_NA_NA_NA_NA_NA_NA_NA_NA_NA_NA_NA	0.35	0.74	-0.10	-0.87	0.74	-1.13	0.07	0	0.33	0	0.16
cellular organisms_Bacteria_Proteobacteria_Alphaproteobacteria_Rickettsiales_Rickettsiaceae_Rickettsieae_Rickettsia_spotted fever group_Rickettsia felis_NA_NA_NA_NA_NA_NA_NA_NA_NA_NA_NA	-2.91	-1.11	-4.46	-3.32	2.73	-1.15	0.07	0	0.33	0	0.17
cellular organisms_Bacteria_Proteobacteria_Alphaproteobacteria_Rhizobiales_Hyphomicrobiaceae_Hyphomicrobium_Hyphomicrobium sp. CS1GBMeth3_NA_NA_NA_NA_NA_NA_NA_NA_NA_NA_NA_NA	-0.18	-0.85	0.75	1.52	0.83	1.65	0.09	0	0.25	0	0.18
cellular organisms_Bacteria_Proteobacteria_Gammaproteobacteria_Pseudomonadales_Ventosimonadaceae_Ventosimonas_Ventosimonas gracilis_NA_NA_NA_NA_NA_NA_NA_NA_NA_NA_NA_NA	0.76	0.30	1.21	0.98	0.85	1.13	0.09	0	0.33	0	0.19
cellular organisms_Bacteria_Proteobacteria_Gammaproteobacteria_Thiotrichales_Piscirickettsiaceae_Cycloclasticus_Cycloclasticus sp. symbiont of Bathymodiolus heckerae_NA_NA_NA_NA_NA_NA_NA_NA_NA_NA_NA_NA	1.11	0.37	1.69	1.30	0.97	1.29	0.08	0	0.29	0	0.19
cellular organisms_Bacteria_Terrabacteria group_Firmicutes_Clostridia_Clostridiales_Peptostreptococcaceae_Peptostreptococcus_environmental samples_Peptostreptococcus anaerobius CAG:621_NA_NA_NA_NA_NA_NA_NA_NA_NA_NA	-0.34	0.35	-1.02	-1.61	1.54	-1.02	0.10	0	0.37	0	0.19
cellular organisms_Bacteria_Proteobacteria_Gammaproteobacteria_Oceanospirillales_Alcanivoracaceae_Marinicella_Marinicella sp. F2_NA_NA_NA_NA_NA_NA_NA_NA_NA_NA_NA_NA	1.93	1.45	2.51	1.06	0.88	1.18	0.10	0	0.31	0	0.20

```r
# Load the taxa profiles determined with Metaphlan3 as a table with OTU in columns and samples as rows
raw_metagenome_Metaphlan3 <- read.csv('https://raw.githubusercontent.com/mesnage/MixtureTox/main/mixture_S21.csv'
)
raw_metagenome_Metaphlan3 <- data.frame(raw_metagenome_Metaphlan3[,-c(1,2)], row.names = raw_metagenome_Metaphlan
3$X)
raw_metagenome_Metaphlan3 <- as.data.frame(t(raw_metagenome_Metaphlan3))

# Filter rare taxa
raw_metagenome_Metaphlan3[raw_metagenome_Metaphlan3 == 0] <- NA
raw_metagenome_Metaphlan3 <- raw_metagenome_Metaphlan3 %>% dplyr::select(which(colMeans(is.na(.)) < 0.2))
raw_metagenome_Metaphlan3[is.na(raw_metagenome_Metaphlan3)] <- 0

# Make Kruskall Wallis tests because the data is not provided as counts
prov_metagenome <- cbind(metadata_group = mixture_metadata$Group, raw_metagenome_Metaphlan3)

P_value_prov <- vector() ; outcome_list <- vector() ; P_value <- data.frame()
for (i in c(2:ncol(prov_metagenome)))
{
  outcome <- colnames(prov_metagenome)[i]
  P_value_prov <- kruskal.test(get(outcome) ~ prov_metagenome$metadata_group, data = prov_metagenome)$p.value
  P_value <- rbind(P_value, P_value_prov)
  outcome_list <- append(outcome_list, outcome)
}
results <- data.frame(outcome_list, P_value)

colnames(results)[2] <- "P_value"
library(qvalue)
results$adjp_anova_r <- p.adjust(results$P_value, method = 'BH')

head(results, 10) %>%
  kbl(caption = "MetaPhlan3 taxa which are the most different between the two groups") %>%
  kable_classic(c("striped", "hover"), full_width = F, html_font = "Cambria")
```

MetaPhlan3 taxa which are the most different between the two groups

outcome_list	P_value	adjp_anova_r
s__Adlercreutzia_equolifaciens	0.2040239	0.5610656
s__Anaerotruncus_sp_G3_2012	0.5633634	0.6033318
s__Asaccharobacter_celatus	0.3556111	0.6033318
s__Bifidobacterium_animalis	0.5637029	0.6033318
s__Bifidobacterium_pseudolongum	0.1329893	0.5610656
s__Collinsella_intestinalis	0.4179096	0.6033318
s__Collinsella_stercoris	0.6033318	0.6033318
s__Enterorhabdus_caecimuris	0.5066281	0.6033318
s__Lactobacillus_johnsonii	0.6033318	0.6033318
s__Lactobacillus_murinus	0.2040239	0.5610656

```r
# Using KO_level3
raw_KO_level3 <- read.csv('https://raw.githubusercontent.com/mesnage/MixtureTox/main/mixture_S23.csv')
raw_KO_level3 <- data.frame(raw_KO_level3[,-1], row.names = raw_KO_level3$level3)
raw_KO_level3 <- as.data.frame(t(raw_KO_level3))

# Filter rare pathways
raw_KO_level3[raw_KO_level3 == 0] <- NA
raw_KO_level3 <- raw_KO_level3 %>% dplyr::select(which(colMeans(is.na(.)) < 0.2))
raw_KO_level3[is.na(raw_KO_level3)] <- 0
raw_KO_level3 <- t(raw_KO_level3)

# Perform Aldex search
aldex_KO_level3 <- aldex(raw_KO_level3, mixture_metadata$Group, mc.samples=128, test="t", effect=TRUE, include.sa
mple.summary=FALSE, denom="iqlr", verbose=FALSE)
```

```
## aldex.clr: generating Monte-Carlo instances and clr values
```

```
## operating in serial mode
```

```
## computing iqlr centering
```

```
## aldex.ttest: doing t-test
```

```
## aldex.effect: calculating effect sizes
```

```r
aldex_KO_level3 <- aldex_KO_level3[order(aldex_KO_level3$wi.eBH),]
aldex_KO_level3 <- round(aldex_KO_level3, digits = 2)

head(aldex_KO_level3, 10)  %>%
  kbl(caption = "The 10 KO annotations which are the most different between the two groups") %>%
  kable_classic(c("striped", "hover"), full_width = F, html_font = "Cambria")
```

The 10 KO annotations which are the most different between the two groups

	rab.all	rab.win.CON	rab.win.MIX	diff.btw	diff.win	effect	overlap	we.ep	we.eBH	wi.ep	wi.eBH
05014 Amyotrophic lateral sclerosis (ALS) [PATH:ko05014]	-7.68	-5.63	-8.83	-3.70	3.30	-1.08	0.09	0.00	0.08	0.00	0.08
03050 Proteasome [PATH:ko03050]	-3.11	-2.58	-3.73	-1.16	1.08	-0.98	0.14	0.00	0.14	0.00	0.17
04540 Gap junction [PATH:ko04540]	-5.71	-4.19	-6.87	-2.41	2.41	-0.89	0.16	0.00	0.18	0.00	0.21
00362 Benzoate degradation [PATH:ko00362]	0.08	0.00	0.24	0.24	0.30	0.82	0.20	0.01	0.26	0.01	0.27
04144 Endocytosis [PATH:ko04144]	-3.95	-3.05	-4.70	-1.37	1.99	-0.74	0.21	0.02	0.36	0.01	0.31
04113 Meiosis - yeast [PATH:ko04113]	0.47	0.18	0.96	0.83	1.12	0.71	0.22	0.01	0.37	0.02	0.33
03060 Protein export [PATH:ko03060]	2.12	2.00	2.17	0.17	0.28	0.57	0.23	0.04	0.42	0.02	0.34
00550 Peptidoglycan biosynthesis [PATH:ko00550]	5.47	5.40	5.50	0.11	0.20	0.46	0.23	0.17	0.51	0.03	0.34
04111 Cell cycle - yeast [PATH:ko04111]	-7.04	-5.99	-7.89	-1.81	2.47	-0.63	0.24	0.05	0.41	0.04	0.34
00260 Glycine, serine and threonine metabolism [PATH:ko00260]	6.41	6.36	6.44	0.09	0.16	0.61	0.23	0.03	0.41	0.03	0.34

```r
# Using KO_function
raw_KO_function <- read.csv('https://raw.githubusercontent.com/mesnage/MixtureTox/main/mixture_S25.csv')
raw_KO_function <- data.frame(raw_KO_function[,-1], row.names = raw_KO_function$ko_function)
raw_KO_function <- as.data.frame(t(raw_KO_function))

# Filter rare pathways
raw_KO_function[raw_KO_function == 0] <- NA
raw_KO_function <- raw_KO_function %>% dplyr::select(which(colMeans(is.na(.)) < 0.2))
raw_KO_function[is.na(raw_KO_function)] <- 0
raw_KO_function <- t(raw_KO_function)

# Perform Aldex search
aldex_KO_function <- aldex(raw_KO_function, mixture_metadata$Group, mc.samples=128, test="t", effect=TRUE, includ
e.sample.summary=FALSE, denom="iqlr", verbose=FALSE)
```

```
## aldex.clr: generating Monte-Carlo instances and clr values
```

```
## operating in serial mode
```

```
## computing iqlr centering
```

```
## aldex.ttest: doing t-test
```

```
## aldex.effect: calculating effect sizes
```

```r
aldex_KO_function <- aldex_KO_function[order(aldex_KO_function$wi.eBH),]
aldex_KO_function <- round(aldex_KO_function, digits = 2)

head(aldex_KO_function, 10)  %>%
  kbl(caption = "The 10 pathways which are the most different between the two groups") %>%
  kable_classic(c("striped", "hover"), full_width = F, html_font = "Cambria")
```

The 10 pathways which are the most different between the two groups

	rab.all	rab.win.CON	rab.win.MIX	diff.btw	diff.win	effect	overlap	we.ep	we.eBH	wi.ep	wi.eBH
PTS-Glc-EIIA, crr; PTS system, glucose-specific IIA component [EC:2.7.1.69]	0.20	0.52	-0.26	-0.72	0.63	-0.99	0.14	0.02	0.75	0.00	0.66
RP-L35, rpmI; large subunit ribosomal protein L35	2.24	2.15	2.37	0.25	0.30	0.78	0.17	0.01	0.74	0.01	0.66
rbsB; ribose transport system substrate-binding protein	2.70	2.56	3.00	0.46	0.47	0.92	0.15	0.00	0.74	0.00	0.66
EEF1A; elongation factor 1-alpha	-4.02	-2.57	-4.88	-2.31	2.51	-0.90	0.16	0.01	0.74	0.01	0.67
PTS-Bgl-EIIB, bglF; PTS system, beta-glucosides-specific IIB component [EC:2.7.1.69]	1.75	2.10	1.56	-0.53	0.51	-0.85	0.16	0.03	0.78	0.00	0.67
PTS-Bgl-EIIA, bglF; PTS system, beta-glucosides-specific IIA component [EC:2.7.1.69]	1.92	2.13	1.73	-0.47	0.68	-0.61	0.17	0.06	0.88	0.00	0.67
mrcB; penicillin-binding protein 1B [EC:2.4.1.129 3.4.-.-]	0.16	-0.31	0.42	0.82	1.11	0.75	0.17	0.01	0.74	0.01	0.67
E3.5.4.13, dcd; dCTP deaminase [EC:3.5.4.13]	-0.11	0.04	-0.41	-0.52	0.60	-0.79	0.18	0.01	0.74	0.01	0.68
cobN; cobaltochelatase CobN [EC:6.6.1.2]	0.06	0.36	-0.30	-0.63	0.65	-0.86	0.17	0.01	0.74	0.01	0.68
HSPA1_8; heat shock 70kDa protein 1/8	-2.30	-1.15	-3.21	-1.89	2.31	-0.83	0.18	0.01	0.74	0.01	0.68

```r
# Plot the pathways tryptophan and nicotinamide
plotdata <- raw_KO_level3
plotdata[is.na(plotdata)] <- 0
plotdata <- t(plotdata)
plotdata <- data.frame(condition = mixture_metadata$Group, plotdata)

# Plot the pathway tryptophan
ggplot(plotdata, aes(x= condition, y=`X00380.Tryptophan.metabolism..PATH.ko00380.`, color= condition, fill=condit
ion)) + ylab('Tryptophan.metabolism PATH.ko00380') +
  geom_boxplot(outlier.shape = NA, alpha = 0.3)  + theme_bw() +  scale_fill_manual(values=c("grey", "firebrick"))
+ geom_jitter(size = 0.5, position=position_jitter(0.2)) +
  theme(text = element_text(size=8), axis.title.x=element_blank(), axis.text.x = element_blank(), legend.position
= "none") +
  scale_color_manual(values=c("black", "firebrick2"))
```

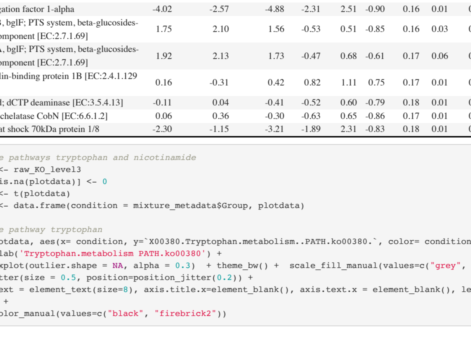


```r
# Plot the pathway nicotinamide
ggplot(plotdata, aes(x= condition, y=`X00760.Nicotinate.and.nicotinamide.metabolism..PATH.ko00760.`, color= condi
tion, fill=condition)) + ylab('Nicotinamide metabolism PATH.ko00760.') +
  geom_boxplot(outlier.shape = NA, alpha = 0.3)  + theme_bw() +  scale_fill_manual(values=c("grey", "firebrick"))
+
geom_jitter(size = 0.5, position=position_jitter(0.2)) +
  theme(text = element_text(size=8), axis.title.x=element_blank(), axis.text.x = element_blank(), legend.position
= "none") +
  scale_color_manual(values=c("black", "firebrick2"))
```

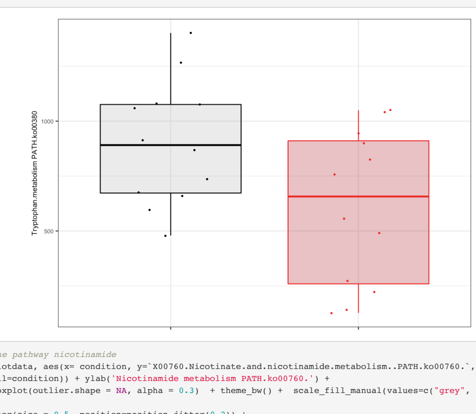

Liver transcriptomics

RNA-seq data was analysed with Salmon. This tool was used to quantify transcript abundance by mapping the reads against a reference transcriptome (Ensembl Release Rattus Norvegicus 6.0 cDNA fasta). Mapping rate was 82.0 ± 4.4% on a rat transcriptome index containing 31,196 targets. The Salmon output was then imported in R as described in this Markdown using the Bioconductor package tximport.

We created a transcript database containing transcript counts, which was used to perform a differential gene expression analysis using DESeq2. We finally used goseq to perform a gene ontology analysis accounting for transcript length biases.

In this markdown, the files generated using Salmon, counting the number of reads mapping to each gene of the rat transcriptome, are downloaded from my github repository.

The following markdown describes the different steps for this analysis. A Transcriptome file is created with all the raw and processed data. It is composed of:

Transcriptome$abundance : Available as File S27 Transcriptome$counts: Available as File S28 Transcriptome
$length : Available as File S29 Transcriptome$gtf_file: Available as File S30 Transcriptome
$sampleinfo : Available as Excel S31 Transcriptome$DESeq_normalized_counts : Available as File S32 Transcriptome
$res_mixtureOrdered : Available as File S33 Transcriptome$KEGG: Available as File S34 Transcriptome$GO.wall: Available as File S35

The raw data from the transcriptomics analysis is available at GEO accession number GSE157426.

```r
# Create a directory to store the output of salmon
dir.create("sf", showWarnings = TRUE, recursive = FALSE)

# Download the Salmon quantification files for the control group
download.file('https://raw.githubusercontent.com/mesnage/MixtureTox/main/transcriptome/GC-RM-8672-BT5011-1_quant.
sf', 'sf/GC-RM-8672-BT5011-1_quant.sf')
download.file('https://raw.githubusercontent.com/mesnage/MixtureTox/main/transcriptome/GC-RM-8672-BT5011-2_quant.
sf', 'sf/GC-RM-8672-BT5011-2_quant.sf')
download.file('https://raw.githubusercontent.com/mesnage/MixtureTox/main/transcriptome/GC-RM-8672-BT5011-3_quant.
sf', 'sf/GC-RM-8672-BT5011-3_quant.sf')
download.file('https://raw.githubusercontent.com/mesnage/MixtureTox/main/transcriptome/GC-RM-8672-BT5011-4_quant.
sf', 'sf/GC-RM-8672-BT5011-4_quant.sf')
download.file('https://raw.githubusercontent.com/mesnage/MixtureTox/main/transcriptome/GC-RM-8672-BT5011-5_quant.
sf', 'sf/GC-RM-8672-BT5011-5_quant.sf')
download.file('https://raw.githubusercontent.com/mesnage/MixtureTox/main/transcriptome/GC-RM-8672-BT5011-6_quant.
sf', 'sf/GC-RM-8672-BT5011-6_quant.sf')
download.file('https://raw.githubusercontent.com/mesnage/MixtureTox/main/transcriptome/GC-RM-8672-BT5011-7_quant.
sf', 'sf/GC-RM-8672-BT5011-7_quant.sf')
download.file('https://raw.githubusercontent.com/mesnage/MixtureTox/main/transcriptome/GC-RM-8672-BT5011-8_quant.
sf', 'sf/GC-RM-8672-BT5011-8_quant.sf')
download.file('https://raw.githubusercontent.com/mesnage/MixtureTox/main/transcriptome/GC-RM-8672-BT5011-9_quant.
sf', 'sf/GC-RM-8672-BT5011-9_quant.sf')
download.file('https://raw.githubusercontent.com/mesnage/MixtureTox/main/transcriptome/GC-RM-8672-BT5011-10_quant
.sf', 'sf/GC-RM-8672-BT5011-10_quant.sf')
download.file('https://raw.githubusercontent.com/mesnage/MixtureTox/main/transcriptome/GC-RM-8672-BT5011-11_quant
.sf', 'sf/GC-RM-8672-BT5011-11_quant.sf')
download.file('https://raw.githubusercontent.com/mesnage/MixtureTox/main/transcriptome/GC-RM-8672-BT5011-12_quant
.sf', 'sf/GC-RM-8672-BT5011-12_quant.sf')

# Download the Salmon quantification files for the mixture group
download.file('https://raw.githubusercontent.com/mesnage/MixtureTox/main/transcriptome/GC-RM-8672-BT5011-141_quan
t.sf', 'sf/GC-RM-8672-BT5011-141_quant.sf')
download.file('https://raw.githubusercontent.com/mesnage/MixtureTox/main/transcriptome/GC-RM-8672-BT5011-142_quan
t.sf', 'sf/GC-RM-8672-BT5011-142_quant.sf')
download.file('https://raw.githubusercontent.com/mesnage/MixtureTox/main/transcriptome/GC-RM-8672-BT5011-143_quan
t.sf', 'sf/GC-RM-8672-BT5011-143_quant.sf')
download.file('https://raw.githubusercontent.com/mesnage/MixtureTox/main/transcriptome/GC-RM-8672-BT5011-144_quan
t.sf', 'sf/GC-RM-8672-BT5011-144_quant.sf')
download.file('https://raw.githubusercontent.com/mesnage/MixtureTox/main/transcriptome/GC-RM-8672-BT5011-145_quan
t.sf', 'sf/GC-RM-8672-BT5011-145_quant.sf')
download.file('https://raw.githubusercontent.com/mesnage/MixtureTox/main/transcriptome/GC-RM-8672-BT5011-146_quan
t.sf', 'sf/GC-RM-8672-BT5011-146_quant.sf')
download.file('https://raw.githubusercontent.com/mesnage/MixtureTox/main/transcriptome/GC-RM-8672-BT5011-147_quan
t.sf', 'sf/GC-RM-8672-BT5011-147_quant.sf')
download.file('https://raw.githubusercontent.com/mesnage/MixtureTox/main/transcriptome/GC-RM-8672-BT5011-148_quan
t.sf', 'sf/GC-RM-8672-BT5011-148_quant.sf')
download.file('https://raw.githubusercontent.com/mesnage/MixtureTox/main/transcriptome/GC-RM-8672-BT5011-149_quan
t.sf', 'sf/GC-RM-8672-BT5011-149_quant.sf')
download.file('https://raw.githubusercontent.com/mesnage/MixtureTox/main/transcriptome/GC-RM-8672-BT5011-150_quan
t.sf', 'sf/GC-RM-8672-BT5011-150_quant.sf')
download.file('https://raw.githubusercontent.com/mesnage/MixtureTox/main/transcriptome/GC-RM-8672-BT5011-151_quan
t.sf', 'sf/GC-RM-8672-BT5011-151_quant.sf')
download.file('https://raw.githubusercontent.com/mesnage/MixtureTox/main/transcriptome/GC-RM-8672-BT5011-152_quan
t.sf', 'sf/GC-RM-8672-BT5011-152_quant.sf')

dirs <- list.files("sf/")
quant_files <- list.files("sf/",pattern="quant.sf",recursive = TRUE,full.names = TRUE)
names(quant_files) <- dirs
quant_files
```

```
##         GC-RM-8672-BT5011-1_quant.sf        GC-RM-8672-BT5011-10_quant.sf
##   "sf//GC-RM-8672-BT5011-1_quant.sf"  "sf//GC-RM-8672-BT5011-10_quant.sf"
##        GC-RM-8672-BT5011-11_quant.sf        GC-RM-8672-BT5011-12_quant.sf
##  "sf//GC-RM-8672-BT5011-11_quant.sf"  "sf//GC-RM-8672-BT5011-12_quant.sf"
##       GC-RM-8672-BT5011-141_quant.sf       GC-RM-8672-BT5011-142_quant.sf
## "sf//GC-RM-8672-BT5011-141_quant.sf" "sf//GC-RM-8672-BT5011-142_quant.sf"
##       GC-RM-8672-BT5011-143_quant.sf       GC-RM-8672-BT5011-144_quant.sf
## "sf//GC-RM-8672-BT5011-143_quant.sf" "sf//GC-RM-8672-BT5011-144_quant.sf"
##       GC-RM-8672-BT5011-145_quant.sf       GC-RM-8672-BT5011-146_quant.sf
## "sf//GC-RM-8672-BT5011-145_quant.sf" "sf//GC-RM-8672-BT5011-146_quant.sf"
##       GC-RM-8672-BT5011-147_quant.sf       GC-RM-8672-BT5011-148_quant.sf
## "sf//GC-RM-8672-BT5011-147_quant.sf" "sf//GC-RM-8672-BT5011-148_quant.sf"
##       GC-RM-8672-BT5011-149_quant.sf       GC-RM-8672-BT5011-150_quant.sf
## "sf//GC-RM-8672-BT5011-149_quant.sf" "sf//GC-RM-8672-BT5011-150_quant.sf"
##       GC-RM-8672-BT5011-151_quant.sf       GC-RM-8672-BT5011-152_quant.sf
## "sf//GC-RM-8672-BT5011-151_quant.sf" "sf//GC-RM-8672-BT5011-152_quant.sf"
##         GC-RM-8672-BT5011-2_quant.sf         GC-RM-8672-BT5011-3_quant.sf
##   "sf//GC-RM-8672-BT5011-2_quant.sf"   "sf//GC-RM-8672-BT5011-3_quant.sf"
##         GC-RM-8672-BT5011-4_quant.sf         GC-RM-8672-BT5011-5_quant.sf
##   "sf//GC-RM-8672-BT5011-4_quant.sf"   "sf//GC-RM-8672-BT5011-5_quant.sf"
##         GC-RM-8672-BT5011-6_quant.sf         GC-RM-8672-BT5011-7_quant.sf
##   "sf//GC-RM-8672-BT5011-6_quant.sf"   "sf//GC-RM-8672-BT5011-7_quant.sf"
##         GC-RM-8672-BT5011-8_quant.sf         GC-RM-8672-BT5011-9_quant.sf
##   "sf//GC-RM-8672-BT5011-8_quant.sf"   "sf//GC-RM-8672-BT5011-9_quant.sf"
```

```r
# Inspecting the salmon output
quants <- read_tsv(quant_files[1])
```

```
## Parsed with column specification:
## cols(
##   Name = col_character(),
##   Length = col_double(),
##   EffectiveLength = col_double(),
##   TPM = col_double(),
##   NumReads = col_double()
## )
```

```r
head(quants)
```

```
## # A tibble: 6 x 5
##   Name                 Length EffectiveLength    TPM NumReads
##   <chr>                 <dbl>           <dbl>  <dbl>    <dbl>
## 1 ENSRNOT00000047550.4    955           752.   4929.    36503
## 2 ENSRNOT00000040993.4   1039           836.   4346.    35780
## 3 ENSRNOT00000050156.3   1545          1342.  22256.   294175
## 4 ENSRNOT00000043693.3    684           481.  12494.    59184
## 5 ENSRNOT00000046201.3    204            37.1 17643.     6449
## 6 ENSRNOT00000046108.3    681           478.  12954.    60985
```

```r
# Download the sample information allocating the Salmon files a rat ID
sampleinfo <- read.csv('https://raw.githubusercontent.com/mesnage/MixtureTox/main/mixture_S31.csv')
rownames(sampleinfo) <- sampleinfo$run

# Download the GTF files
download.file('ftp://ftp.ensembl.org/pub/release-99/gtf/rattus_norvegicus/Rattus_norvegicus.Rnor_6.0.99.gtf.gz',
'Rattus_norvegicus.Rnor_6.0.99.gtf.gz')
R.utils::gunzip('Rattus_norvegicus.Rnor_6.0.99.gtf.gz')
gtf_file <- "Rattus_norvegicus.Rnor_6.0.99.gtf"
file.exists(gtf_file)
```

```
## [1] TRUE
```

```r
# Create the Tx database
txdb <- makeTxDbFromGFF(gtf_file)
```

```
## Import genomic features from the file as a GRanges object ...
```

```
## OK
```

```
## Prepare the 'metadata' data frame ... OK
## Make the TxDb object ... OK
```

```r
k <- keys(txdb, keytype="TXNAME")
tx_map <- AnnotationDbi::select(txdb, keys = k, columns="GENEID", keytype = "TXNAME")
```

```
## 'select()' returned 1:1 mapping between keys and columns
```

```r
head(tx_map)
```

```
##               TXNAME             GENEID
## 1 ENSRNOT00000044187 ENSRNOG00000046319
## 2 ENSRNOT00000072186 ENSRNOG00000046319
## 3 ENSRNOT00000093216 ENSRNOG00000046319
## 4 ENSRNOT00000072360 ENSRNOG00000047964
## 5 ENSRNOT00000077244 ENSRNOG00000058808
## 6 ENSRNOT00000087443 ENSRNOG00000061316
```

```r
# Fixing the problem with transcript names - the hard way as recommended from TX tutorial
quants <- separate(quants, Name, c("TXNAME","Number"),remove = FALSE)
head(quants)
```

```
## # A tibble: 6 x 7
##   Name               TXNAME         Number Length EffectiveLength    TPM NumReads
##   <chr>              <chr>          <chr>   <dbl>           <dbl>  <dbl>    <dbl>
## 1 ENSRNOT000000475… ENSRNOT000000… 4         955            752.   4929.    36503
## 2 ENSRNOT000000409… ENSRNOT000000… 4        1039            836.   4346.    35780
## 3 ENSRNOT000000501… ENSRNOT000000… 3        1545           1342.  22256.   294175
## 4 ENSRNOT000000436… ENSRNOT000000… 3         684            481.  12494.    59184
## 5 ENSRNOT000000462… ENSRNOT000000… 3         204             37.1 17643.     6449
## 6 ENSRNOT000000461… ENSRNOT000000… 3         681            478.  12954.    60985
```

```r
quants <- dplyr:::left_join(quants, tx_map, by="TXNAME")
head(quants)
```

```
## # A tibble: 6 x 8
##   Name       TXNAME     Number Length EffectiveLength    TPM NumReads GENEID
##   <chr>      <chr>      <chr>   <dbl>           <dbl>  <dbl>    <dbl> <chr>
## 1 ENSRNOT00… ENSRNOT00… 4         955            752.   4929.    36503 ENSRNOG00…
## 2 ENSRNOT00… ENSRNOT00… 4        1039            836.   4346.    35780 ENSRNOG00…
## 3 ENSRNOT00… ENSRNOT00… 3        1545           1342.  22256.   294175 ENSRNOG00…
## 4 ENSRNOT00… ENSRNOT00… 3         684            481.  12494.    59184 ENSRNOG00…
## 5 ENSRNOT00… ENSRNOT00… 3         204             37.1 17643.     6449 ENSRNOG00…
## 6 ENSRNOT00… ENSRNOT00… 3         681            478.  12954.    60985 ENSRNOG00…
```

```r
tx2gene <- dplyr:::select(quants, Name, GENEID)
head(tx2gene)
```

```
## # A tibble: 6 x 2
##   Name                 GENEID
##   <chr>                <chr>
## 1 ENSRNOT00000047550.4 ENSRNOG00000030644
## 2 ENSRNOT00000040993.4 ENSRNOG00000031033
## 3 ENSRNOT00000050156.3 ENSRNOG00000034234
## 4 ENSRNOT00000043693.3 ENSRNOG00000030371
## 5 ENSRNOT00000046201.3 ENSRNOG00000033299
## 6 ENSRNOT00000046108.3 ENSRNOG00000031979
```

```r
any(is.na(tx2gene$GENEID))
```

```
## [1] FALSE
```

```r
tx2gene <- dplyr:::filter(tx2gene, !is.na(GENEID))
transcriptome <- tximport(quant_files,type="salmon",tx2gene = tx2gene)
```

```
## reading in files with read_tsv
## 1 2 3 4 5 6 7 8 9 10 11 12 13 14 15 16 17 18 19 20 21 22 23 24
## summarizing abundance
## summarizing counts
## summarizing length
```

```r
# Double check that the samples are actually what we call them
all(rownames(sampleinfo) == colnames(transcriptome$counts))
```

```
## [1] TRUE
```

```r
# Create the DESeq object
dds_mixture <- DESeqDataSetFromTximport(transcriptome,
                                        colData = sampleinfo,
                                        design <- ~group)
```

```
## using counts and average transcript lengths from tximport
```

```r
# Filter genes with less than 5 counts in 5 samples
keep <- rowSums(assay(dds_mixture) >= 5) >= 5
dds_mixture <- dds_mixture[keep,]

# DESeq
dds_mixture <- DESeq(dds_mixture)
```

```
## estimating size factors
## using 'avgTxLength' from assays(dds), correcting for library size
## estimating dispersions
## gene-wise dispersion estimates
## mean-dispersion relationship
## final dispersion estimates
## fitting model and testing
## -- replacing outliers and refitting for 549 genes
## -- DESeq argument 'minReplicatesForReplace' = 7
## -- original counts are preserved in counts(dds)
## estimating dispersions
## fitting model and testing
```

```r
DESeq_normalized_counts <- counts(dds_mixture, normalized=TRUE)
resultsNames(dds_mixture)
```

```
## [1] "Intercept"                "group_Mixture_vs_Control"
```

```r
# Extract the DESeq results
res_mixture <- results(dds_mixture)
res_mixtureOrdered <- res_mixture[order(res_mixture$pvalue),]
res_mixtureOrdered <- as.data.frame(res_mixtureOrdered)
res_mixtureOrdered <- res_mixtureOrdered[!is.na(res_mixtureOrdered$padj),]
res_mixtureOrdered$gene_id <- row.names(res_mixtureOrdered)

# Use a parsed GTF file to found the correspondance between gene ID and their annotations
gtf_file <- read.table('https://raw.githubusercontent.com/mesnage/MixtureTox/main/transcriptome/Rattus_norvegicus
.Rnor_6.0.99_parsed.txt', header = TRUE, sep = '\t')
res_mixtureOrdered <- dplyr:::right_join(gtf_file, res_mixtureOrdered, by = 'gene_id')

# Calculate the fold change
res_mixtureOrdered$FC <- ifelse(res_mixtureOrdered$log2FoldChange > 0, 2 ^ res_mixtureOrdered$log2FoldChange, -1
/ (2 ^ res_mixtureOrdered$log2FoldChange))

# Create the transcriptome object storing the results
transcriptome[["gtf_file"]] <- gtf_file
transcriptome[["sampleinfo"]] <- sampleinfo
transcriptome[["dds_mixture"]] <- dds_mixture
transcriptome[["DESeq_normalized_counts"]] <- DESeq_normalized_counts
transcriptome[["res_mixtureOrdered"]] <- res_mixtureOrdered

head(transcriptome$res_mixtureOrdered, 10)  %>%
  kbl(caption = "Liver transcriptome of Sprague-Dawley rats exposed for 90 days to the mixture of 6 pesticides. G
enes were considered as differentially expressed if their count were found to be statistically significant after
```

```
an analysis with DESeq2") %>%
  kable_classic(c("striped", "hover"), full_width = F, html_font = "Cambria")
```

Liver transcriptome of Sprague-Dawley rats exposed for 90 days to the mixture of 6 pesticides. Genes were considered as differentially expressed if their count were found to be statistically significant after an analysis with DESeq2

gene_id	gene_name	gene_source	gene_biotype	baseMean	log2FoldChange	lfcSE	stat	pvalue	padj	FC
ENSRNOG00000046912	Nr1d2	ensembl	protein_coding	348.59172	1.9987836	0.2048919	9.755310	0	0e+00	3.996629
ENSRNOG00000009329	Nr1d1	ensembl	protein_coding	334.06115	3.5117556	0.3737693	9.395517	0	0e+00	11.406273
ENSRNOG00000000521	Cdkn1a	ensembl	protein_coding	217.91660	-1.9491025	0.2406261	-8.100128	0	0e+00	-3.861343
ENSRNOG00000013982	Hsd17b2	ensembl	protein_coding	1935.11617	1.7525842	0.2449805	7.153974	0	0e+00	3.369616
ENSRNOG00000020836	Rorc	ensembl	protein_coding	1330.82404	-0.8863935	0.1254991	-7.062948	0	0e+00	-1.848549
ENSRNOG00000051819	Sdr42e1	ensembl	protein_coding	694.31982	0.7951325	0.1128193	7.047842	0	0e+00	1.735237
ENSRNOG00000056135	Tsc22d3	ensembl	protein_coding	399.07871	-1.0417687	0.1503882	-6.927195	0	0e+00	-2.058750
ENSRNOG00000007387	Per1	ensembl	protein_coding	39.79224	-1.6010386	0.2435943	-6.572562	0	1e-07	-3.033616
ENSRNOG00000045553	Proser2	ensembl_havana	protein_coding	114.08685	-0.8024729	0.1230456	-6.521752	0	1e-07	-1.744088
ENSRNOG00000010165	Tnfaip2	ensembl	protein_coding	238.13862	0.9479086	0.1492952	6.349225	0	2e-07	1.929074

```
# Classify the genes as statistially significant or not
res_mixtureOrdered <- transcriptome$res_mixtureOrdered
res_mixtureOrdered$Significant <- ifelse(res_mixtureOrdered$FC > 1.5 & res_mixtureOrdered$padj < 0.05 | res_mixtu
reOrdered$FC < -1.5 & res_mixtureOrdered$padj < 0.05, "FDR < 0.05", "Not Sig")
res_mixtureOrdered$Very_Significant <- ifelse(res_mixtureOrdered$FC > 1.5 & res_mixtureOrdered$padj < 0.01 | res_
mixtureOrdered$FC < -1.5 & res_mixtureOrdered$padj < 0.01, "FDR < 0.01", "Not Sig")

# Create a volcano plot using the DESeq results
ggplot(res_mixtureOrdered, aes(x = log2FoldChange, y = -log10(pvalue))) +
  geom_point(aes(color = Significant)) +
  labs(xlab("Log2 fold change"), ylab("-log10 p-value"))  +
  scale_color_manual(values = c("red", "grey")) +
  theme_bw(base_size = 12) + theme(legend.position = "bottom") +
  ggrepel::geom_text_repel(
    data = subset(res_mixtureOrdered, Very_Significant == "FDR < 0.01"),
    aes(label = gene_name),
    size = 3,
    box.padding = unit(0.35, "lines"),
    point.padding = unit(0.3, "lines"))
```

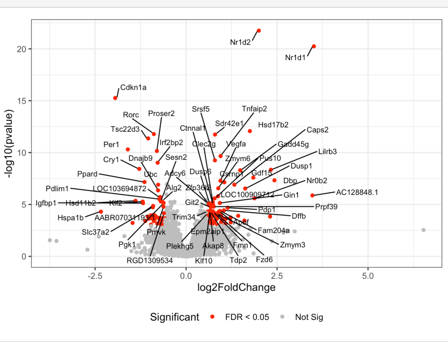


```
res_mixtureOrdered$Significant <- ifelse(res_mixtureOrdered$FC > 1.5 & res_mixtureOrdered$padj < 0.05 | res_mixtu
reOrdered$FC < -1.5 & res_mixtureOrdered$padj < 0.05, "FDR < 0.05", "Not Sig")

# Goseq enrichment
library(Biobase) ; library(goseq) ; library("org.Hs.eg.db")

# Remove duplicates
res_mixtureOrdered <- res_mixtureOrdered[!duplicated(res_mixtureOrdered$gene_name), ]

# Classify the genes as statistially significant or not
genes = as.integer(ifelse(res_mixtureOrdered$FC > 1.5 & res_mixtureOrdered$padj < 0.05 | res_mixtureOrdered$FC <
-1.5 & res_mixtureOrdered$padj < 0.05, 1, 0))

# GO BP analysis
names(genes)= res_mixtureOrdered$gene_name
pwf=nullp(genes,"rn4","geneSymbol")
```

```
## Loading rn4 length data...
```

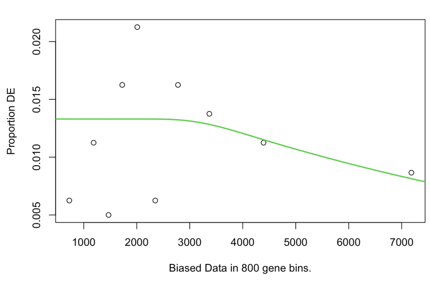


```
GO.wall=goseq(pwf,"rn4","geneSymbol", test.cats=c("GO:BP"), use_genes_without_cat=TRUE)
```

```
## Fetching GO annotations...
```

```
## Calculating the p-values...
```

```
## 'select()' returned 1:1 mapping between keys and columns
```

```
GO.wall$padjust = p.adjust(GO.wall$over_represented_pvalue,method="BH")

head(GO.wall, 10)  %>%
  kbl(caption = "Gene Ontology (GO) terms enriched among the differentially expressed genes") %>%
  kable_classic(c("striped", "hover"), full_width = F, html_font = "Cambria")
```

Gene Ontology (GO) terms enriched among the differentially expressed genes

	category	over_represented_pvalue	under_represented_pvalue	numDEInCat	numInCat	term	ontology	padjust
12034	GO:1901360	0e+00	1.0000000	70	3556	organic cyclic compound metabolic process	BP	0.0001186
1238	GO:0006139	0e+00	1.0000000	65	3294	nucleobase-containing compound metabolic process	BP	0.0001979
3502	GO:0019219	1e-07	1.0000000	50	2208	regulation of nucleobase-containing compound metabolic process	BP	0.0001979
7209	GO:0045935	1e-07	1.0000000	35	1237	positive regulation of nucleobase-containing compound metabolic process	BP	0.0001979
1630	GO:0006725	1e-07	1.0000000	66	3415	cellular aromatic compound metabolic process	BP	0.0001979
2418	GO:0009725	1e-07	1.0000000	28	846	response to hormone	BP	0.0001979
4820	GO:0032870	1e-07	1.0000000	21	512	cellular response to hormone stimulus	BP	0.0002640
7429	GO:0046483	2e-07	0.9999999	65	3390	heterocycle metabolic process	BP	0.0002860
7784	GO:0048545	2e-07	1.0000000	17	344	response to steroid hormone	BP	0.0003208
8306	GO:0051254	6e-07	0.9999998	31	1111	positive regulation of RNA metabolic process	BP	0.0008687

```
# KEGG pathway analysis
KEGG=goseq(pwf,'rn4','geneSymbol',test.cats="KEGG", use_genes_without_cat=TRUE)
```

```
## Fetching GO annotations...
```

```
## Calculating the p-values...
```

```
head(KEGG)
```

```
##     category over_represented_pvalue under_represented_pvalue numDEInCat
## 158    04710            4.592284e-05                0.9999985          4
## 114    04115            1.747315e-03                0.9998401          4
## 163    04742            4.933361e-03                0.9998598          2
## 30     00360            7.704010e-03                0.9997157          2
## 112    04110            1.670121e-02                0.9970269          4
## 29     00350            2.080881e-02                0.9986314          2
##     numInCat
## 158       19
## 114       47
## 163       10
## 30        12
## 112       90
## 29        20
```

```
KEGG$padjust = p.adjust(KEGG$over_represented_pvalue,method="BH")

head(KEGG, 10)  %>%
  kbl(caption = "KEGG terms enriched among the differentially expressed genes") %>%
  kable_classic(c("striped", "hover"), full_width = F, html_font = "Cambria")
```

KEGG terms enriched among the differentially expressed genes

	category	over_represented_pvalue	under_represented_pvalue	numDEInCat	numInCat	padjust
158	04710	0.0000459	0.9999985	4	19	0.0103326
114	04115	0.0017473	0.9998401	4	47	0.1965729
163	04742	0.0049334	0.9998598	2	10	0.3700021
30	00360	0.0077040	0.9997157	2	12	0.4333505
112	04110	0.0167012	0.9970269	4	90	0.7515542
29	00350	0.0208088	0.9986314	2	20	0.7803305
99	03320	0.0319707	0.9949679	3	62	0.8360025
15	00140	0.0320868	0.9972973	2	25	0.8360025
174	04960	0.0350712	0.9968803	2	28	0.8360025
210	05219	0.0397799	0.9962042	2	29	0.8360025

We also used orthogonal partial least squares discriminant analysis (OPLS-DA) to evaluate the predictive ability of each omics approach. The R package ropls version 1.20.0 was used. This algorithm uses the nonlinear iterative partial least squares algorithm (NIPALS). Since PLS-DA methods are prone to overfitting, we assessed the significance of our classification using permutation tests (permuted 1,000 times).

```
# Make the OPLS-DA for the liver transcriptome profiles
transcriptome_counts <- t(transcriptome$abundance)
labellm <- transcriptome$sampleinfo$group
transcriptome_counts <- data.frame(labellm, transcriptome_counts)
labellm <- transcriptome_counts$labellm
labellm <- factor(labellm)
transcriptome_counts <- transcriptome_counts[,-1]
transcriptome.oplsda <- opls(transcriptome_counts, labellm, orthoI = NA, predI = 1, permI = 1000)
```

```
## OPLS-DA
## 24 samples x 18170 variables and 1 response
## standard scaling of predictors and response(s)
## 5317 excluded variables (near zero variance)
##       R2X(cum) R2Y(cum) Q2(cum)  RMSEE pre ort  pR2Y   pQ2
## Total    0.522    0.999   0.583 0.0198   1   3 0.044 0.001
```

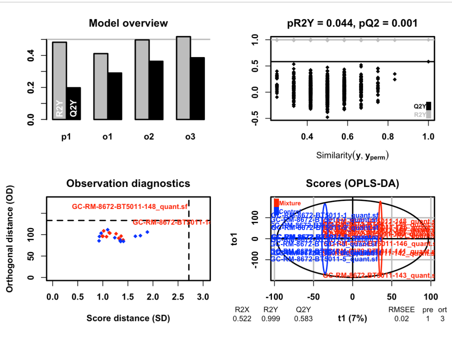

Liver methylation

DNA methylation calls from RRBS data were extracted with Bismark. The output from Bismark was then imported in R and analysed with Methylkit. DNA methylation calls were annotated using RefSeq gene predictions for rats (rn6 release) with the package genomation. Other annotations were retrieved using the genome wide annotation for rat tool org.Rn.eg.dbR package version 3.8.2. Statistical analysis was performed with logistic regression models fitted per CpG using Methylkit functions. P-values were adjusted to Q-values using SLIM method.

The raw data from the RRBS analysis is available at GEO accession number GSE157551.

The output from Bismark is downloaded from GitHub. Note that 5 files were over the 25Mb size limit for GitHub. In order to allow their use, they have been broken down for their storage on GitHub. A bashscript is included in this Markdown to concatenate back the outputs of the 5 files.

```
# Download the files fron GitHub, and unzip them

download.file('https://raw.githubusercontent.com/mesnage/MixtureTox/main/methylation/GC-RM-8674-BT5011-1-F-215254
.bismark.cov.gz', 'CON_01.cov.gz')
R.utils::gunzip('CON_01.cov.gz')

download.file('https://raw.githubusercontent.com/mesnage/MixtureTox/main/methylation/GC-RM-8674-BT5011-2-F-215271
.bismark.cov.gz', 'CON_02.cov.gz')
R.utils::gunzip('CON_02.cov.gz')

download.file('https://raw.githubusercontent.com/mesnage/MixtureTox/main/methylation/GC-RM-8674-BT5011-3-F-215286
.bismark.cov.gz', 'CON_03.cov.gz')
R.utils::gunzip('CON_03.cov.gz')

download.file('https://raw.githubusercontent.com/mesnage/MixtureTox/main/methylation/GC-RM-8674-BT5011-4-F-215301
.bismark.cov.gz', 'CON_04.cov.gz')
R.utils::gunzip('CON_04.cov.gz')

download.file('https://raw.githubusercontent.com/mesnage/MixtureTox/main/methylation/GC-RM-8674-BT5011-5-F-215328
.bismark.cov.gz', 'CON_05.cov.gz')
R.utils::gunzip('CON_05.cov.gz')

download.file('https://raw.githubusercontent.com/mesnage/MixtureTox/main/methylation/GC-RM-8674-BT5011-6-F-215347
.bismark.cov.gz', 'CON_06.cov.gz')
R.utils::gunzip('CON_06.cov.gz')

download.file('https://raw.githubusercontent.com/mesnage/MixtureTox/main/methylation/GC-RM-8674-BT5011-7-F-215373
.bismark.cov.gz', 'CON_07.cov.gz')
R.utils::gunzip('CON_07.cov.gz')

download.file('https://raw.githubusercontent.com/mesnage/MixtureTox/main/methylation/GC-RM-8674-BT5011-8-F-215392
.bismark.cov.gz', 'CON_08.cov.gz')
R.utils::gunzip('CON_08.cov.gz')

download.file('https://raw.githubusercontent.com/mesnage/MixtureTox/main/methylation/GC-RM-8674-BT5011-9-F-215417
.bismark.cov.gz', 'CON_09.cov.gz')
R.utils::gunzip('CON_09.cov.gz')

download.file('https://raw.githubusercontent.com/mesnage/MixtureTox/main/methylation/GC-RM-8674-BT5011-10-F-21543
4_head.bismark.cov.gz', 'CON_10_head.cov.gz')
R.utils::gunzip('CON_10_head.cov.gz')

download.file('https://raw.githubusercontent.com/mesnage/MixtureTox/main/methylation/GC-RM-8674-BT5011-10-F-21543
4_tail.bismark.cov.gz', 'CON_10_tail.cov.gz')
R.utils::gunzip('CON_10_tail.cov.gz')

download.file('https://raw.githubusercontent.com/mesnage/MixtureTox/main/methylation/GC-RM-8674-BT5011-11-F-21545
9_head.bismark.cov.gz', 'CON_11_head.cov.gz')
R.utils::gunzip('CON_11_head.cov.gz')

download.file('https://raw.githubusercontent.com/mesnage/MixtureTox/main/methylation/GC-RM-8674-BT5011-11-F-21545
9_tail.bismark.cov.gz', 'CON_11_tail.cov.gz')
R.utils::gunzip('CON_11_tail.cov.gz')

download.file('https://raw.githubusercontent.com/mesnage/MixtureTox/main/methylation/GC-RM-8674-BT5011-12F-215474
.bismark.cov.gz', 'CON_12.cov.gz')
R.utils::gunzip('CON_12.cov.gz')

download.file('https://raw.githubusercontent.com/mesnage/MixtureTox/main/methylation/GC-RM-8674-BT5011-141-F-2152
61_head.bismark.cov.gz', 'MIX_01_head.cov.gz')
R.utils::gunzip('MIX_01_head.cov.gz')

download.file('https://raw.githubusercontent.com/mesnage/MixtureTox/main/methylation/GC-RM-8674-BT5011-141-F-2152
61_tail.bismark.cov.gz', 'MIX_01_tail.cov.gz')
R.utils::gunzip('MIX_01_tail.cov.gz')

download.file('https://raw.githubusercontent.com/mesnage/MixtureTox/main/methylation/GC-RM-8674-BT5011-142-F-2152
78.bismark.cov.gz', 'MIX_02.cov.gz')
R.utils::gunzip('MIX_02.cov.gz')

download.file('https://raw.githubusercontent.com/mesnage/MixtureTox/main/methylation/GC-RM-8674-BT5011-143-F-2153
00.bismark.cov.gz', 'MIX_03.cov.gz')
R.utils::gunzip('MIX_03.cov.gz')

download.file('https://raw.githubusercontent.com/mesnage/MixtureTox/main/methylation/GC-RM-8674-BT5011-144-F-2153
27.bismark.cov.gz', 'MIX_04.cov.gz')
R.utils::gunzip('MIX_04.cov.gz')

download.file('https://raw.githubusercontent.com/mesnage/MixtureTox/main/methylation/GC-RM-8674-BT5011-145-F-2153
46_head.bismark.cov.gz', 'MIX_05_head.cov.gz')
R.utils::gunzip('MIX_05_head.cov.gz')

download.file('https://raw.githubusercontent.com/mesnage/MixtureTox/main/methylation/GC-RM-8674-BT5011-145-F-2153
46_tail.bismark.cov.gz', 'MIX_05_tail.cov.gz')
R.utils::gunzip('MIX_05_tail.cov.gz')

download.file('https://raw.githubusercontent.com/mesnage/MixtureTox/main/methylation/GC-RM-8674-BT5011-146-F-2153
66.bismark.cov.gz', 'MIX_06.cov.gz')
R.utils::gunzip('MIX_06.cov.gz')

download.file('https://raw.githubusercontent.com/mesnage/MixtureTox/main/methylation/GC-RM-8674-BT5011-147-F-2153
91.bismark.cov.gz', 'MIX_07.cov.gz')
R.utils::gunzip('MIX_07.cov.gz')

download.file('https://raw.githubusercontent.com/mesnage/MixtureTox/main/methylation/GC-RM-8674-BT5011-148-F-2154
16.bismark.cov.gz', 'MIX_08.cov.gz')
R.utils::gunzip('MIX_08.cov.gz')

download.file('https://raw.githubusercontent.com/mesnage/MixtureTox/main/methylation/GC-RM-8674-BT5011-149-F-2154
33_tail.bismark.cov.gz', 'MIX_09_tail.cov.gz')
R.utils::gunzip('MIX_09_tail.cov.gz')

download.file('https://raw.githubusercontent.com/mesnage/MixtureTox/main/methylation/GC-RM-8674-BT5011-149-F-2154
33_head.bismark.cov.gz', 'MIX_09_head.cov.gz')
R.utils::gunzip('MIX_09_head.cov.gz')

download.file('https://raw.githubusercontent.com/mesnage/MixtureTox/main/methylation/GC-RM-8674-BT5011-150-F-2154
58.bismark.cov.gz', 'MIX_10.cov.gz')
R.utils::gunzip('MIX_10.cov.gz')

download.file('https://raw.githubusercontent.com/mesnage/MixtureTox/main/methylation/GC-RM-8674-BT5011-151-F-2154
73.bismark.cov.gz', 'MIX_11.cov.gz')
R.utils::gunzip('MIX_11.cov.gz')

download.file('https://raw.githubusercontent.com/mesnage/MixtureTox/main/methylation/GC-RM-8674-BT5011-152-F-2154
98.bismark.cov.gz', 'MIX_12.cov.gz')
R.utils::gunzip('MIX_12.cov.gz')
```

```
cat CON_11_head.cov CON_11_tail.cov > CON_11.cov
cat CON_10_head.cov CON_10_tail.cov > CON_10.cov
cat MIX_01_head.cov MIX_01_tail.cov > MIX_01.cov
cat MIX_05_head.cov MIX_05_tail.cov > MIX_05.cov
cat MIX_09_head.cov MIX_09_tail.cov > MIX_09.cov
```

```
# Load the methylkit dataset
metadata <- read.csv('https://raw.githubusercontent.com/mesnage/MixtureTox/main/mixture_S01.csv')

gene.obj=readTranscriptFeatures('https://raw.githubusercontent.com/mesnage/MixtureTox/main/methylation/rn6.ncbiRe
fSeq.bed')
```

```
## Reading the table...
```

```
## Calculating intron coordinates...
```

```
## Calculating exon coordinates...
```

```
## Calculating TSS coordinates...
```

```
## Calculating promoter coordinates...
```

```
## Outputting the final GRangesList...
```

```
Sample <- metadata$Methylation
files <- paste0(Sample,".cov")


metadata$Group[metadata$Group == 'CON'] <- 0
metadata$Group[metadata$Group == 'MIX'] <- 1

# Create the methylkit object
myobj=methRead(as.list(files),
               as.list(metadata$Methylation),
               assembly="rn6",
               treatment=as.integer(metadata$Group),
               context="CpG",
               mincov = 10, pipeline = "bismarkCoverage")
```

```
## Received list of locations.
```

```
## Reading file.
## Reading file.
## Reading file.
## Reading file.
## Reading file.
## Reading file.
## Reading file.
## Reading file.
## Reading file.
## Reading file.
## Reading file.
## Reading file.
## Reading file.
## Reading file.
## Reading file.
## Reading file.
## Reading file.
## Reading file.
## Reading file.
## Reading file.
## Reading file.
## Reading file.
## Reading file.
## Reading file.
```

```
# Plot the histogram for percent methylation distribution. Typically, percent methylation histogram should have t
wo peaks on both ends
getMethylationStats(myobj[[1]],plot=TRUE,both.strands=FALSE)
```

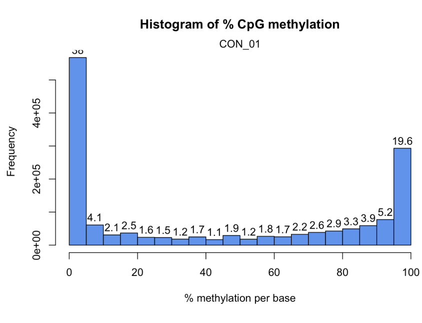


```
# Plot read coverage per base information
getCoverageStats(myobj[[1]],plot=TRUE,both.strands=FALSE)
```

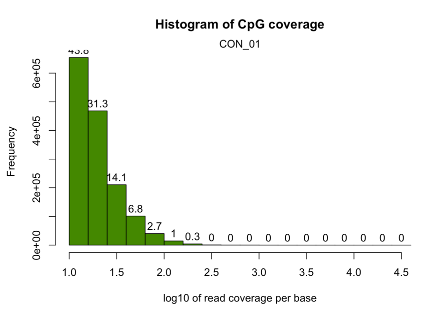


```
# Filtering samples based on read coverage
myobj=filterByCoverage(myobj,lo.count=15,lo.perc=NULL, hi.count=NULL,hi.perc=99.9)

# Merging samples
meth=unite(myobj, destrand=FALSE)
```

```
## uniting...
```

```
#Finding differentially methylated bases or regions
myDiff=calculateDiffMeth(meth)
```

```
## two groups detected:
##  will calculate methylation difference as the difference of
## treatment (group: 1) - control (group: 0)
```

```
myDiffp.hyper=getMethylDiff(myDiff,difference=10,qvalue=0.05,type="hyper")
myDiffp.hypo=getMethylDiff(myDiff,difference=10,qvalue=0.05,type="hypo")
myDiffp =getMethylDiff(myDiff,difference=10,qvalue=0.05)

circos.initializeWithIdeogram(species = "rn6")
bed_list = list(data.frame(myDiffp.hyper)[,c(1:3)], data.frame(myDiffp.hypo)[,c(1:3)])
circos.genomicRainfall(bed_list[1], pch = 16, cex = 0.6, track.height = 0.1, col = c("#FF000080"))
circos.genomicRainfall(bed_list[2], pch = 16, cex = 0.6, track.height = 0.1, col = c("#0000FF80"))
```

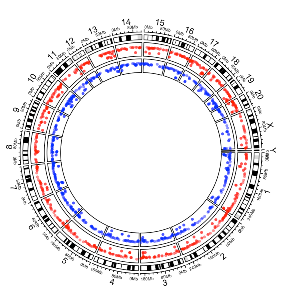

```
# Summary of target set annotation with genic parts
annotateWithGeneParts(as(myDiffp,"GRanges"),gene.obj)
```

```
## Summary of target set annotation with genic parts
```

```
## Rows in target set: 1532
```

```
## -----------------------
```

```
## percentage of target features overlapping with annotation:
```

```
##   promoter       exon     intron intergenic
##       6.01       7.96      38.90      53.13
```

```
##
## percentage of target features overlapping with annotation:
```

```
## (with promoter > exon > intron precedence):
```

```
##   promoter       exon     intron intergenic
##       6.01       5.74      35.12      53.13
```

```
##
```

```
## percentage of annotation boundaries with feature overlap:
```

```
## promoter     exon   intron
##     0.18     0.04     0.26
```

```
##
```

```
## summary of distances to the nearest TSS:
```

```
##    Min. 1st Qu.  Median    Mean 3rd Qu.    Max.
##       6    7317   23726   59430   59642 1527395
```

```
##
```

```
diffpAnn=annotateWithGeneParts(as(myDiffp,"GRanges"),gene.obj)
diffpAnn_table <- getAssociationWithTSS(diffpAnn)
```

```
# Percentage DNA methylation profile shows a bimodal distribution of methylation calls for each sample
perc.meth <- data.frame(percMethylation(meth))
perc.meth <- reshape2::melt(perc.meth)
```

```
## No id variables; using all as measure variables
```

```
ggplot(perc.meth, aes(x = value, y = variable, fill = variable)) +
  geom_density_ridges(scale = 4, alpha = .3) + xlab("% methylation per base") + ylab("sample") +
  scale_fill_cyclical(values = c("black", "black", "black", "black", "black", "black",
                                 "black", "black","black", "black","black", "black",
                                 "firebrick","firebrick","firebrick","firebrick","firebrick","firebrick",
                                 "firebrick","firebrick","firebrick","firebrick","firebrick","firebrick")) +
  theme(legend.position = NULL) + theme_bw()
```

```
## Picking joint bandwidth of 3.84
```

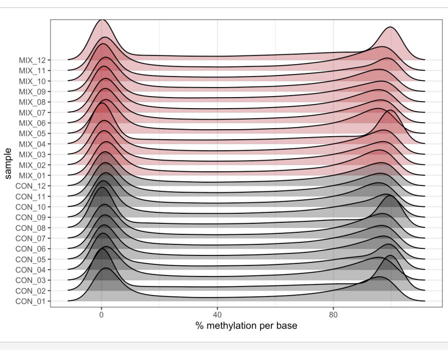


```
# Does CpG methylation decreases around transcription start sites?
annotateWithGeneParts(as(myDiff,"GRanges"),gene.obj)
```

```
## Summary of target set annotation with genic parts
```

```
## Rows in target set: 77008
```

```
## -----------------------
```

```
## percentage of target features overlapping with annotation:
```

```
##   promoter       exon     intron intergenic
##      12.65       9.02      40.05      49.30
```

```
##
## percentage of target features overlapping with annotation:
```

```
## (with promoter > exon > intron precedence):
```

```
##   promoter       exon     intron intergenic
##      12.65       5.08      32.96      49.30
```

```
##
```

```
## percentage of annotation boundaries with feature overlap:
```

```
## promoter     exon   intron
##     9.03     0.83     3.92
```

```
##
```

```
## summary of distances to the nearest TSS:
```

```
##    Min. 1st Qu.  Median    Mean 3rd Qu.    Max.
##       0    5112   21372   63780   64529 1665135
```

```
##
```

```
diffAnn=annotateWithGeneParts(as(myDiff,"GRanges"),gene.obj)
diffAnn_table <- getAssociationWithTSS(diffAnn)

perc.meth <- data.frame(getAssociationWithTSS(diffAnn), percMethylation(meth))
perc.meth <- perc.meth %>% group_by(dist.to.feature) %>% summarise_at(vars(CON_01:MIX_12), mean, na.rm = TRUE)

i <- abs(perc.meth$dist.to.feature) < 10000
cpg_tss <- perc.meth[i,]
cpg_tss <- reshape2::melt(cpg_tss, id.vars = c("dist.to.feature"))
ggplot(data = cpg_tss, aes(dist.to.feature, value, group = variable, colour = variable)) + geom_smooth(se = FALSE
, size = 0.3) +
  theme_bw() + ylab("Percentage CpG methylation") + xlab("distance to TSS") +
  scale_color_manual(values=c("black", "black", "black", "black", "black", "black",
                              "black", "black","black", "black","black", "black",
                              "firebrick","firebrick","firebrick","firebrick","firebrick","firebrick",
                              "firebrick","firebrick","firebrick","firebrick","firebrick","firebrick")) +
  theme(legend.position = "none")
```

```
## `geom_smooth()` using method = 'gam' and formula 'y ~ s(x, bs = "cs")'
```

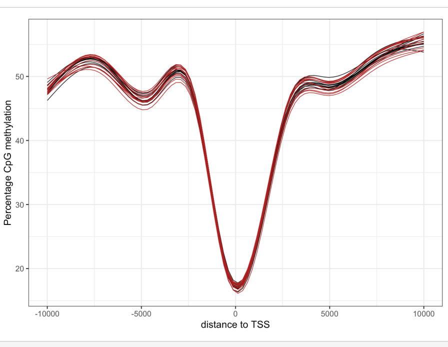


```
# CpG methylation sample clustering
clusterSamples(meth, dist="correlation", method="ward", plot=TRUE)
```

```
## The "ward" method has been renamed to "ward.D"; note new "ward.D2"
```

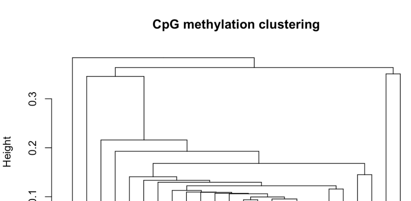

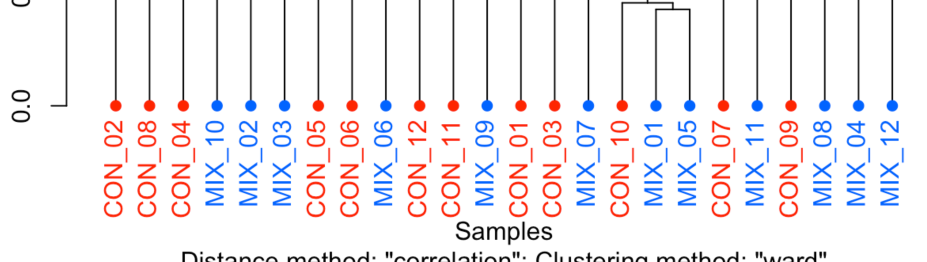

Distance method: "correlation"; Clustering method: "ward"

```
##
## Call:
## hclust(d = d, method = HCLUST.METHODS[hclust.method])
##
## Cluster method   : ward.D
## Distance         : pearson
## Number of objects: 24
```

```
# Volcano plots of differentially methylated CpG sites
annotateWithGeneParts(as(myDiff,"GRanges"),gene.obj)
```

```
## Summary of target set annotation with genic parts
```

```
## Rows in target set: 77008
```

```
## -----------------------
```

```
## percentage of target features overlapping with annotation:
```

```
##   promoter       exon     intron intergenic
##      12.65       9.02      40.05      49.30
```

```
##
## percentage of target features overlapping with annotation:
```

```
## (with promoter > exon > intron precedence):
```

```
##   promoter       exon     intron intergenic
##      12.65       5.08      32.96      49.30
```

```
##
```

```
## percentage of annotation boundaries with feature overlap:
```

```
## promoter     exon   intron
##     9.03     0.83     3.92
```

```
##
```

```
## summary of distances to the nearest TSS:
```

```
##    Min. 1st Qu.  Median    Mean 3rd Qu.    Max.
##       0    5112   21372   63780   64529 1665135
```

```
##
```

```
diffAnn=annotateWithGeneParts(as(myDiff,"GRanges"),gene.obj)
diffAnn_table <- getAssociationWithTSS(diffAnn)

# Match annotations
result_methylation_table <- data.frame(myDiff, diffAnn_table)
result_methylation_table$feature.name<- sub("\\..*","\\",result_methylation_table$feature.name)
result_methylation_table$gene.name <- mapIds(org.Rn.eg.db, result_methylation_table$feature.name, keytype="REFSEQ
", column="GENENAME")
```

```
## 'select()' returned 1:1 mapping between keys and columns
```

```
result_methylation_table$go.name <- mapIds(org.Rn.eg.db, result_methylation_table$feature.name, keytype="REFSEQ",
column="GO")
```

```
## 'select()' returned 1:many mapping between keys and columns
```

```
result_methylation_table$Significant <- ifelse(abs(result_methylation_table$meth.diff) > 10 & result_methylation_
table$qvalue < 0.05, "FDR < 0.05", "Not Sig")

ggplot(result_methylation_table, aes(x = meth.diff, y = -log10(pvalue))) +
  geom_point(aes(color = Significant)) +
  xlab("Percentage methylation change") + ylab("-log10 p-value") +
  scale_color_manual(values = c("red", "grey")) +
  theme_bw(base_size = 12) + theme(legend.position = "bottom") + theme_bw()
```

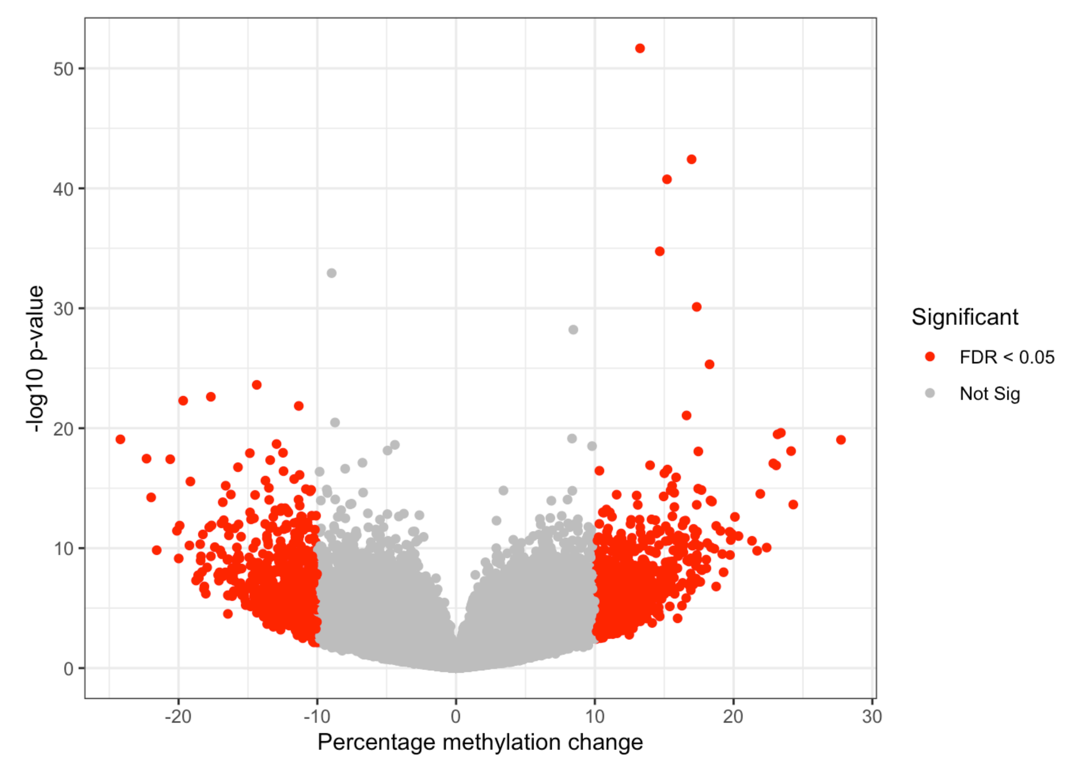


```
# Promoter specific analysis
promoters=regionCounts(myobj,gene.obj$promoters)
meth_promoters=unite(promoters, destrand=FALSE)
```

```
## uniting...
```

```
# Finding differentially methylated bases or regions
myDiff_meth_promoters=calculateDiffMeth(meth_promoters)
```

```
## two groups detected:
##  will calculate methylation difference as the difference of
## treatment (group: 1) - control (group: 0)
```

```
# Summary of target set annotation with genic parts
annotateWithGeneParts(as(myDiff_meth_promoters,"GRanges"),gene.obj)
```

```
## Summary of target set annotation with genic parts
```

```
## Rows in target set: 11228
```

```
## -----------------------
```

```
## percentage of target features overlapping with annotation:
```

```
##   promoter       exon     intron intergenic
##     100.00     100.00      95.54       0.00
```

```
##
## percentage of target features overlapping with annotation:
```

```
## (with promoter > exon > intron precedence):
```

```
##   promoter       exon     intron intergenic
##        100          0          0          0
```

```
##
```

```
## percentage of annotation boundaries with feature overlap:
```

```
## promoter     exon   intron
##    22.89     3.18     3.36
```

```
##
```

```
## summary of distances to the nearest TSS:
```

```
##    Min. 1st Qu.  Median    Mean 3rd Qu.    Max.
##       0       0       0       0       0       0
```

```
##
```

```
diffAnn_promoters=annotateWithGeneParts(as(myDiff_meth_promoters,"GRanges"),gene.obj)
diffAnn_table_promoters <- getAssociationWithTSS(diffAnn_promoters)

# Match annotations
result_methylation_table_promoters <- data.frame(myDiff_meth_promoters, diffAnn_table_promoters)
result_methylation_table_promoters$feature.name<- sub("\\..*","\\",result_methylation_table_promoters$feature.nam
e)
result_methylation_table_promoters$gene.name <- mapIds(org.Rn.eg.db, result_methylation_table_promoters$feature.n
ame, keytype="REFSEQ", column="GENENAME")
```

```
## 'select()' returned 1:1 mapping between keys and columns
```

```
result_methylation_table_promoters$go.name <- mapIds(org.Rn.eg.db, result_methylation_table_promoters$feature.nam
e, keytype="REFSEQ", column="GO")
```

```
## 'select()' returned 1:many mapping between keys and columns
```

```
result_methylation_table_promoters <- result_methylation_table_promoters %>% distinct(feature.name, meth.diff, .k
eep_all = TRUE)

i <- abs(result_methylation_table_promoters$meth.diff) > 10
result_methylation_table_promoters <- result_methylation_table_promoters[i,]
i <- result_methylation_table_promoters$qvalue < 0.05
result_methylation_table_promoters <- result_methylation_table_promoters[i,]
```

```
# Match annotations
result_methylation_table_promoters <- data.frame(myDiff_meth_promoters, diffAnn_table_promoters)
result_methylation_table_promoters$feature.name<- sub("\\..*","\\",result_methylation_table_promoters$feature.nam
e)
result_methylation_table_promoters$gene.name <- mapIds(org.Rn.eg.db, result_methylation_table_promoters$feature.n
ame, keytype="REFSEQ", column="GENENAME")
```

```
## 'select()' returned 1:1 mapping between keys and columns
```

```
result_methylation_table_promoters$go.name <- mapIds(org.Rn.eg.db, result_methylation_table_promoters$feature.nam
e, keytype="REFSEQ", column="GO")
```

```
## 'select()' returned 1:many mapping between keys and columns
```

```
# Fetch transcriptome results
rna_table <-data.frame(transcriptome$res_mixtureOrdered)
rna_table$feature.name <- mapIds(org.Rn.eg.db, rna_table$gene_id, keytype="ENSEMBL", column="REFSEQ")
```

```
## 'select()' returned 1:many mapping between keys and columns
```

```
# Make correlation tables with gene methylation and gene expression
correlation_table <- left_join(result_methylation_table_promoters, rna_table, by = 'feature.name')
correlation_table <- correlation_table[!is.na(correlation_table$gene_id),]
correlation_table <- correlation_table[correlation_table$padj < 0.05,]
correlation_table$log10p_met <- -log10(correlation_table$pvalue.x)

# Plot the correlation between gene expression and their methylation
ggplot(correlation_table, aes(log2FoldChange, meth.diff)) + geom_point(aes(colour = log10p_met)) +
  xlab("fold change in gene expression") + ylab("percentage change in methylation") +
  geom_hline(yintercept=0, linetype="dashed") + geom_vline(xintercept=0, linetype="dashed") + theme_bw() +
  scale_colour_gradient(low = "grey", high = "red", na.value = NA)
```

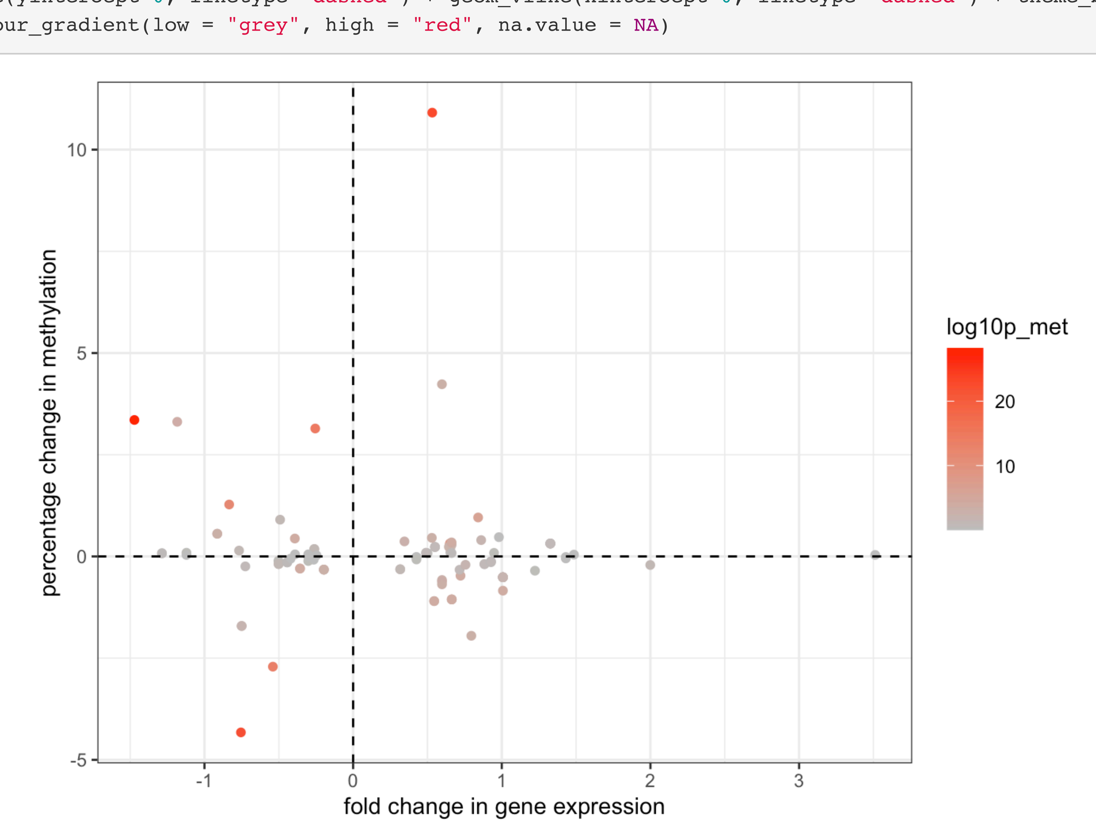


```
# Calculate and test the correlation
cor.test(correlation_table$meth.diff, correlation_table$FC)
```

```
##
##  Pearson's product-moment correlation
##
## data:  correlation_table$meth.diff and correlation_table$FC
## t = -0.12895, df = 101, p-value = 0.8977
## alternative hypothesis: true correlation is not equal to 0
## 95 percent confidence interval:
##  -0.2058439  0.1811441
## sample estimates:
##         cor
## -0.01283034
```
